# The integrated brain network that controls respiration

**DOI:** 10.7554/eLife.83654

**Published:** 2023-03-08

**Authors:** Friedrich Krohn, Manuele Novello, Ruben S van der Giessen, Chris I De Zeeuw, Johan JM Pel, Laurens WJ Bosman

**Affiliations:** 1 Department of Neuroscience, Erasmus MC Rotterdam Netherlands; 2 Department of Neurology, Erasmus MC Rotterdam Netherlands; 3 Netherlands Institute for Neuroscience, Royal Academy of Arts and Sciences Amsterdam Netherlands; Baylor College of Medicine United States; Baylor College of Medicine United States

**Keywords:** Respiration, Central pattern generator, nucleus of the solitary tract, Cerebellum, Pre-Bötzinger complex, network

## Abstract

Respiration is a brain function on which our lives essentially depend. Control of respiration ensures that the frequency and depth of breathing adapt continuously to metabolic needs. In addition, the respiratory control network of the brain has to organize muscular synergies that integrate ventilation with posture and body movement. Finally, respiration is coupled to cardiovascular function and emotion. Here, we argue that the brain can handle this all by integrating a brainstem central pattern generator circuit in a larger network that also comprises the cerebellum. Although currently not generally recognized as a respiratory control center, the cerebellum is well known for its coordinating and modulating role in motor behavior, as well as for its role in the autonomic nervous system. In this review, we discuss the role of brain regions involved in the control of respiration, and their anatomical and functional interactions. We discuss how sensory feedback can result in adaptation of respiration, and how these mechanisms can be compromised by various neurological and psychological disorders. Finally, we demonstrate how the respiratory pattern generators are part of a larger and integrated network of respiratory brain regions.

## Introduction

From the first cry to the last gasp, the respiratory system should never fail to supply sufficient oxygen to meet metabolic demands during every possible event throughout life (Del Negro et al., 2018). The respiratory pattern is not only determined by physical activity, but also reflects the emotional state, and volitional control of respiration can be used to alter affection and reduce stress (Suess et al., 1980; Philippot et al., 2002; Arch and Craske, 2006; Seppälä et al., 2014; Szulczewski, 2019). Indeed, multiple behaviors, such as swimming, playing musical instruments, parturition, or meditation depend on precise respiratory control (Brown and Gerbarg, 2009; Jakovljevic and McConnell, 2009; Bartlett and Leiter, 2012; Holstege, 2014; Sakaguchi and Aiba, 2016), and for many sports and arts, it is often the control of respiration that separates mediocre from top performance (Mahler et al., 1991; Phillips and Aitchison, 1997; Laczika et al., 2013; Salomoni et al., 2016). Thus, indeed, respiratory control affects all aspects of life.

Although control over respiration can be voluntary, most of it is subconscious, even during voluntary respiration. In this review, we discuss the integrated network of brain regions most involved in the control of respiration, their connections, and possible clinical consequences of their pathology. Throughout, we discuss how respiratory control and other motor and non-motor systems interact.

## Respiratory muscles and their motor neurons

**Figure 1.**
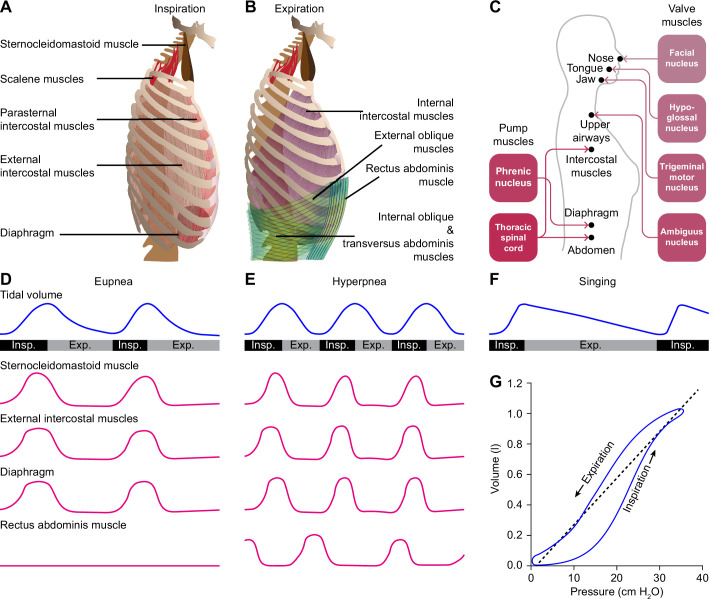
Respiratory muscles and their innervation. (**A**) The main driving force for inspiration is delivered by the diaphragm in conjunction with the external intercostal muscles. Other muscles that can enlarge the chest, such as the parasternal intercostal, sternocleidomastoid and scalene muscles, may also contribute. (**B**) Active expiration involves contraction of the internal intercostal muscles together with abdominal muscles. (**C**) The pump muscles are innervated from the spinal cord, with the phrenic nucleus housing the motor neurons of the diaphragm, and the thoracic spinal cord those of the intercostal and abdominal muscles. (**D**) During regular breathing at rest (eupnea), inspiration is followed by a largely passive form of expiration termed post-inspiration or early expiration. During post-inspiration, the abdominal muscles are not (strongly) involved. (**E**) When the metabolic demand is higher, hyperpnea entails the activation of expiratory pump muscles during active expiration. (**F**) Prolonged post-inspiration, when required assisted by active expiration, ensures a longer period with constant outflow of air as exploited by professional singers. (**G**) Intrapleural pressure-volume curve during normal respiration in which the lung compliance is defined as the slope of the dotted line. Schematized data based on Bellani et al., 2018 and Pitts et al., 2015 (**D–E**), Salomoni et al., 2016 (**F**), and Albaiceta et al., 2005 (**G**). Exp.=expiration, Insp.=inspiration.

Despite their vast differences in body size and ecological niches, mammals possess similar basic mechanics of ventilation, with some variations between species or sexes (Carvalho and Gonçalves, 2011; Torres-Tamayo et al., 2018). Both lung and tidal volumes scale linearly with body weight, and the higher metabolic rate of smaller mammals is accounted for by a faster respiratory rate (Stahl, 1967; Boggs and Tenney, 1984). The force required for inspiration is delivered by so-called pump muscles that expand the rib cage. The diaphragm and external intercostal muscles are the most prominent inspiratory pump muscles, but also parasternal intercostal, sternocleidomastoid and scalene muscles can act as such (De Troyer and Estenne, 1984; De Troyer et al., 1998; De Troyer et al., 2005; Torres-Tamayo et al., 2018; Welch et al., 2019; LoMauro and Aliverti, 2021). During active expiration, expiratory pump muscles, in particular the internal intercostal and abdominal muscles, are active (De Troyer et al., 2005; Mortola, 2013; Welch et al., 2019). The diaphragm is under control of motor neurons in the phrenic nucleus located in the anterior ramus of the third to sixth cervical vertebrae (Wertheimer, 1886; Hollinshead and Keswani, 1956; Wu et al., 2017). The intercostal and abdominal muscles are innervated from the thoracic spinal cord (Figure 1A–C).

Valve muscles regulate the air flow by adjusting the resistance of the upper airways. Activity of the facial nucleus can lead to opening of the nasal valve, via the dilator naris anterior muscle and the alar part of the nasal muscle (van Dishoeck, 1937; Strohl, 1985; Vaiman et al., 2003). Contractions of these muscles do not only facilitate inspiration, but can also relate to sniffing (Welker, 1964). Just before the start of inspiration, motor neurons of the hypoglossal nucleus activate tongue muscles, reducing collapsibility of the pharynx (Fuller et al., 1999; Gestreau et al., 2005). Indeed, tongue deformation, for example as a consequence of excessive fat depositions in obesity, can be related to obstructive sleep apnea (Lowe et al., 1986; Kim et al., 2014; Yu et al., 2021). Activation of the trigeminal motor nucleus can contribute to jaw movements (Mong et al., 1988). Finally, contractions of the larynx muscles show a bimodal pattern; initially, they are dilated to allow airflow into the lungs, while at the end of inspiration these muscles contract to reduce the outflow of air, prolonging the period of gas exchange (Gesell and White, 1938; Gautier et al., 1973; Insalaco et al., 1991; Hutchison et al., 1993; Amis et al., 1995; Dutschmann et al., 2014). Laryngeal constriction is controlled by the nucleus ambiguus via the vagus nerve (Dutschmann et al., 2014). The nucleus ambiguus houses also motor neurons controlling swallowing, the control of which is strongly coupled to that of respiration (McFarland and Lund, 1993; Moore et al., 2014).

Respiratory muscles typically serve multiple functions. Rib cage muscles, for instance, control both respiration and arm movements, so that locomotion and respiration are tightly coupled during quadrupedal locomotion. Indeed, one could argue that the change from quadrupedal to bipedal locomotion during hominid evolution paved the way for the intricate breathing control required for human speech (Carrier, 1984; MacLarnon and Hewitt, 1999).

## Functional anatomy of respiratory control

**Figure 2.**
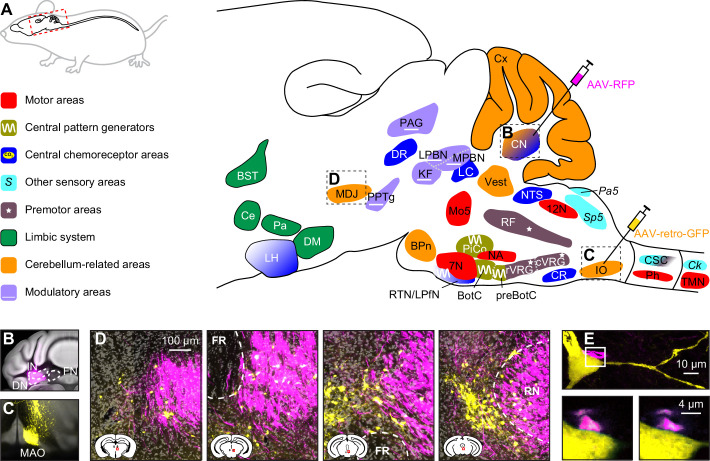
Brain areas involved in subconscious respiratory control. (**A**) The subcortical areas involved in control of respiration were classified according to their main function and plotted at their approximate location on a sagittal projection of the mouse brain. 7N = facial nucleus, 12N=hypoglossal nucleus, BotC = Bötzinger complex, BPn = basal pons, BST = bed nucleus of the stria terminalis, Ce = central amygdala, Ck = Clarke’s column, CN = cerebellar nuclei, CR = caudal raphe nucleus, CSC = cervical spinal cord, cVRG = caudal ventral respiratory group, Cx = cerebellar cortex, DM = dorsomedial hypothalamus, DR = dorsal raphe nucleus, IO = inferior olive, KF = Kölliker-Fuse nucleus, LC = locus coeruleus, LH = lateral hypothalamus, LPBN = lateral parabrachial nucleus, LPfN = lateral parafacial nucleus, MDJ = nuclei of the mesodiencephalic junction, Mo5=trigeminal motor nucleus, MPBN = medial parabrachial nucleus, NA = nucleus ambiguus, NTS = nucleus of the solitary tract, Pa5=paratrigeminal nucleus, PAG = periaqueductal gray, Pa = paraventricular hypothalamus, Ph = phrenic nucleus, PiCo = postinspiratory complex, PPTg = pedunculopontine tegmental area, preBotC = pre-Bötzinger complex, RF = reticular formation, RTN = retrotrapezoid nucleus, rVRG = rostral ventral respiratory group, Sp5 = spinal trigeminal nucleus, TMN = thoracic motor neurons, Vest = vestibular nuclei. Neural tracing can reveal monosynaptic connections between brain regions, as illustrated with an example using an anterograde tracer in the cerebellar nuclei (**B**; see injection needle in panel A, AAV-RFP, pseudocolored in magenta), and a retrograde tracer in the inferior olive (**C**; AAV-retro-GFP, pseudocolored in yellow). DN = dentate nucleus, FN = fastigial nucleus, IN = interposed nucleus, MAO = medial accessory olive. (**D**) Both tracers can be observed in the MDJ, indicating the presence of monosynaptic projections from the cerebellar nuclei to the MDJ and from there to the inferior olive. Sagittal sections from rostral to caudal (see schemes in the lower left corners with red rectangles indicating locations of images). FR = fasciculus retroflexus, RN = red nucleus. (**E**) A neuron (yellow) in the MDJ that projects to the inferior olive. Close to the soma of this neuron, a bouton (magenta) of a neuron originating from the cerebellar nuclei can be seen (insets below, imaged at two levels 0.7 µm apart). Panels B-E originate from a representative mouse and are modified from Figure 7 from Wang et al., 2022.

Below, we describe the brain areas most involved in subconscious control of respiration, and their main connections. Most of these pathways are bilateral, but ipsi- and contralateral projections can differ in strength or in their ratio between excitatory and inhibitory fibers, potentially introducing asymmetries in motor activity (Biancardi et al., 2021). As detailed studies on monosynaptic projections are sparse in humans, we base our summary on animal studies, with earlier descriptions mostly concerning cats, and more recent ones often performed in rats or mice (Figure 2, Supplementary file 1). Connections labeled as sparse in the original papers are not included in this overview.

Many brain regions lack clear borders. In particular when different species are compared, this may lead to some variations in the interpretation of anatomical projections. On top of this, one should also take into account that anatomy and physiology do not always match. For instance, the inspiratory neurons originally considered to be located in the pre-Bötzinger complex are actually distributed around the region of the pre-Bötzinger complex and are partially intermingled with expiratory neurons originally considered to be located in more caudal nuclei (Baertsch et al., 2019). When reading this review, please note that the use of anatomical names is to help orient oneself, but in reality, borders are often fuzzy. Genetic markers may help to define more homogeneous populations of neurons, and when this information was available, we mention that in the text and figures.

Given that the neuronal mechanisms of respiratory rhythm generation are evolutionary well conserved (Cinelli et al., 2013), inter-species differences are expected to be relatively minor (Kastner and Gauthier, 2008). Important exceptions, however, are the elongation of the pharyngeal region, and the development of complex muscle control of pharynx and mouth related to human speech (Duncker, 2001). Since neural control of speech is outside the scope of this review, this will not be further discussed.

## Rhythmic respiration

Quiet breathing, or eupnea, is a rhythmic alternation between inspiration and passive expiration or post-inspiration (Albaiceta et al., 2005; Figure 1G). During periods with higher metabolic demand, hyperpnea occurs, which entails also active expiration (Pitts et al., 2015; Bellani et al., 2018; Figure 1D–E). It must be noted that the different respiratory muscles cover different aspects of the respiratory cycle. As a result, a clear distinction between passive and active expiration cannot be made. For example, during passive expiration, some of the expiratory muscles can be active, and active expiration encompasses passive elastic contractions as well. In humans, post-inspiration can contribute to longer periods of relatively constant air flow as required for speech or singing (MacLarnon and Hewitt, 1999; Watson et al., 2012; Salomoni et al., 2016; Figure 1F). Here, additional muscles become active that are not active during solely post-inspiration. Thus, to what extent the respiratory cycle within the brainstem can indeed be divided into rhythmogenic phases or whether these phases are just seen at the motor output remains topic of further research. As stated, we discuss in this review multiple mechanisms that modulate the rhythmicity of respiration when confronted with respiratory challenges, such as a change in air pressure or increase in CO_2_ concentration (hypercapnia).

**Figure 3.**
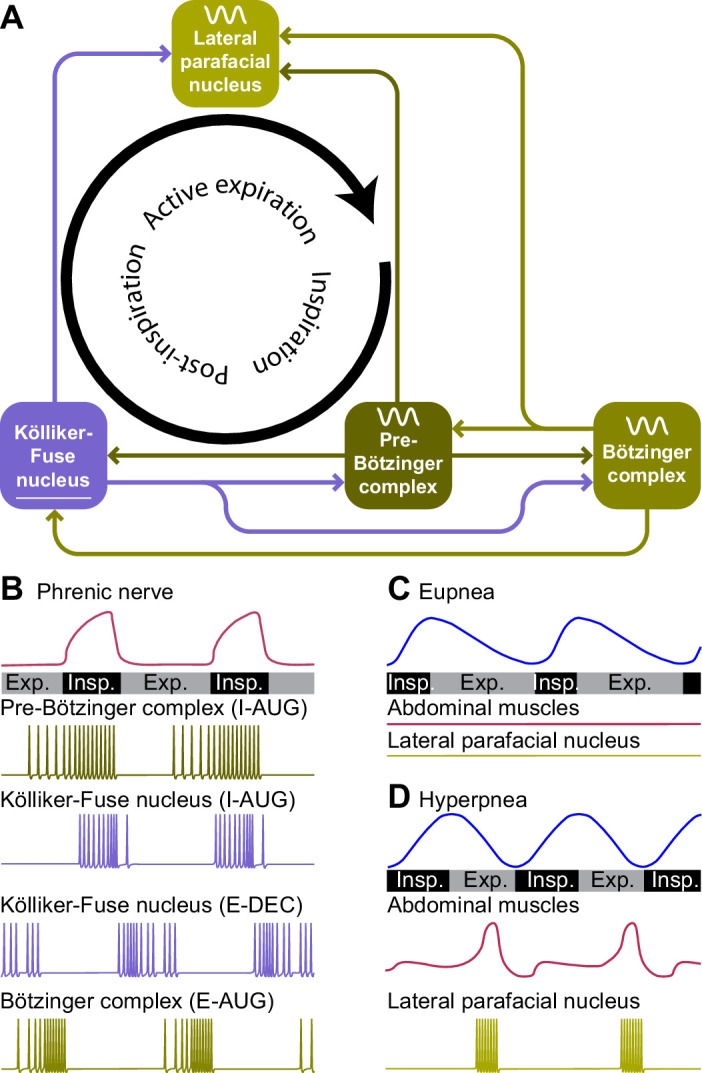
Central pattern generators encode the respiratory rhythm. (**A**) Connections between the pre-Bötzinger complex that organizes inspiration, the Kölliker-Fuse nucleus that relates to the inspiration/expiration switch, the lateral parafacial nucleus that triggers active expiration, and the Bötzinger complex related to expiration. (**B**) Neuronal activity in the central pattern generators varies during the respiratory cycle. The schematized traces represent action potential firing of selected neuronal cell types in relation to activity of the phrenic nerve that drives the main inspiratory pump muscle, the diaphragm. From top to bottom: augmenting inspiratory neurons (I-AUG) from the pre-Bötzinger complex and the Kölliker-Fuse nucleus, a decreasing expiratory neuron (E-DEC) from the Kölliker-Fuse nucleus, and an augmenting expiratory neuron (E-AUG) from the Bötzinger complex. These representations are based on in vitro studies of Marchenko et al., 2016 (pre-Bötzinger complex), Ezure and Tanaka, 2006 (Kölliker-Fuse nucleus), and Flor et al., 2020 (Bötzinger complex). (**C**) During eupnea, thus in the absence of active expiration, neither the expiratory pump muscles of the abdomen, nor the neurons of the lateral parafacial nucleus are active. (**D**) During hyperpnea, thus when active expiration takes place, abdominal expiratory pump muscles are active when the lateral parafacial nucleus neurons produce action potentials. Schematized based on in vivo recordings of anesthetized rats by Pagliardini et al., 2011. Insp.=inspiration, Exp.=expiration.

During regular breathing, the respiratory cycle is determined by brainstem central pattern generators. Inspiration is triggered by activity of neurons in the pre-Bötzinger complex, most of which fire in phase with inspiration and indirectly drive the inspiratory pump muscles (Smith et al., 1991; Guyenet and Wang, 2001; Moore et al., 2013; Del Negro et al., 2018; Yang and Feldman, 2018). Inspiration may be terminated by activation of the Kölliker-Fuse nucleus, and possibly also the postinspiratory complex can contribute to this (Dutschmann and Herbert, 2006; Anderson et al., 2016). Finally, the lateral parafacial nucleus that is silent during eupnea becomes active during active expiration (Pagliardini et al., 2011; Huckstepp et al., 2015; Del Negro et al., 2018; Figure 3). Without input from the brainstem, the spinal circuitry cannot organize respiration. It does contribute to sequential contraction of thoracic muscles to optimize the inflow of air by adjusting muscle control to body biomechanics (Marckwald, 1889; Porter, 1895; Ellenberger and Feldman, 1988; Butler et al., 2014; Shinozaki et al., 2019; Jensen et al., 2019).

### Pre-Bötzinger and Bötzinger complexes

The pre-Bötzinger complex contains a network of neurons generating rhythmic activity that is both essential and sufficient to drive inspiration (Smith et al., 1991; Ramirez et al., 1998; Gray et al., 2001; Tan et al., 2008; Schwarzacher et al., 2011; Ashhad and Feldman, 2020; Dhingra et al., 2020; Figure 3B). In rodents, the pre-Bötzinger complex consists of around 3,000 neurons on each side of the brain, with approximately equal numbers of excitatory and inhibitory neurons (Wallen-Mackenzie et al., 2006; Tan et al., 2008; Yackle et al., 2017). Inspiratory neurons with comparable dynamics may also be found in surrounding regions of the ventral respiratory column, pointing toward a more diffuse spatiotemporal network than originally described (Baertsch et al., 2019). The rhythmogenic kernel is formed by somatostatin-negative (SST^-^) excitatory interneurons (Cui et al., 2016; Ashhad and Feldman, 2020). Excitatory neurons in the pre-Bötzinger complex have a refractory period that prevents them from generating bursts at a high frequency, and this refractory period can be shortened by inhibitory input (Baertsch et al., 2018). Hence, although inhibitory interneurons are not essential for generating rhythmicity, they may modulate the breathing frequency and contribute to the termination of inspiration (Janczewski et al., 2013; Sherman et al., 2015; Hülsmann et al., 2021). The output of the pre-Bötzinger complex is composed of both inhibitory and SST^+^ excitatory neurons.

Although there are direct projections from the pre-Bötzinger complex to multiple respiratory motor nuclei, indirect projections appear to be more common. As such, the phrenic nucleus is predominantly targeted via the rostral ventral respiratory group (rVRG) (Wu et al., 2017), and the thoracic motor neurons that control abdominal muscles via the caudal ventral respiratory group (cVRG) (Gerrits and Holstege, 1996; Yang and Feldman, 2018). In addition, the hypoglossal nucleus is principally reached via the parahypoglossal region of the reticular formation (Chamberlin et al., 2007; Tan et al., 2010; Yang and Feldman, 2018), and the facial nucleus via the intermediate reticular formation (Moore et al., 2004; Koshiya et al., 2014; Yang and Feldman, 2018; Guo et al., 2020).

In addition, there are substantial projections to other respiratory control areas: the Bötzinger complex, Kölliker-Fuse nucleus, postinspiratory complex (PiCo), and lateral parafacial nucleus (Tan et al., 2010; Koshiya et al., 2014; Yang and Feldman, 2018; Biancardi et al., 2021). Furthermore, also the retrotrapezoid nucleus, nucleus tractus solitarii (NTS), lateral and dorsomedial hypothalamus, lateral and medial parabrachial nuclei, and periaqueductal gray are targeted (Tan et al., 2010; Koshiya et al., 2014; Yang and Feldman, 2018; Biancardi et al., 2021; Trevizan-Baú et al., 2021b). A specific subset of *Cdh9*-neurons projects to noradrenergic neurons in the locus coeruleus (Yackle et al., 2017). Finally, there are strong projections to the contralateral pre-Bötzinger complex to promote left/right synchrony during respiration (Wu et al., 2017).

The adjacent Bötzinger complex houses mostly inhibitory neurons that either show decrementing activity during post-inspiration or incrementing activity during expiration, and both groups contribute to the inhibition of inspiratory activity in the pre-Bötzinger complex during expiration (Marchenko et al., 2016; Ausborn et al., 2018; Flor et al., 2020). These activity patterns partially reflect sensory feedback: while augmenting neurons are inhibited by lung inflation, decrementing neurons are excited by it (Manabe and Ezure, 1988; Hayashi et al., 1996). Next to the adjacent pre-Bötzinger complex, also other respiratory control centers are innervated by the Bötzinger complex: the Kölliker-Fuse and lateral parafacial nuclei (Ezure et al., 2003; Yang et al., 2020; Biancardi et al., 2021).

The Bötzinger complex also inhibits premotor areas: rVRG and to a lesser extent also cVRG (Jiang and Lipski, 1990; Bryant et al., 1993; Ezure, 2004), and directly inhibits motor neurons in the phrenic nucleus (Merrill and Fedorko, 1984; Ellenberger et al., 1990b; Tian et al., 1998). Other targets are the NTS, lateral parabrachial nucleus and periaqueductal gray (Merrill et al., 1983; Fedorko and Merrill, 1984; Livingston and Berger, 1989; Smith et al., 1989; Ezure et al., 2003; Trevizan-Baú et al., 2021b).

As the respiratory pattern has to be coordinated with ongoing behavior, the pre-Bötzinger complex receives input from many brain regions, like the Kölliker-Fuse nucleus, PiCo, cVRG, NTS, retrotrapezoid nucleus, locus coeruleus, caudal raphe, lateral and paraventricular hypothalamus, central amygdala, lateral and medial parabrachial nuclei, periaqueductal gray, spinal trigeminal nuclei, and reticular formation (Panneton et al., 2006; Rosin et al., 2006; Jones et al., 2016; Hennessy et al., 2017; Yang et al., 2020; Liu et al., 2021b; Trevizan-Baú et al., 2021b). The pre-Bötzinger complex receives also direct input from the forebrain, including several regions of the neocortex presumably involved in voluntary control of respiration, but these connections are relatively sparse (Yang et al., 2020; Trevizan-Baú et al., 2021a). The inputs of the Bötzinger complex are similar to those of the pre-Bötzinger complex, although less widespread (Gang et al., 1995; Supplementary file 1).

### Kölliker-Fuse nucleus

The pre-Bötzinger complex is not the only area essential for rhythmic respiration. Selective damage to the brainstem at the level of the pons leads to impaired transition from inspiration to expiration, resulting in prolonged periods of inspiration, a condition called apneusis (Marckwald, 1890; Lumsden, 1923). This effect was later localized in parts of the parabrachial complex, initially referred to as pneumotaxic center, and later as the pontine respiratory group (Cohen and Wang, 1959; Caille et al., 1981; Ezure and Tanaka, 2006; Zuperku et al., 2017; Varga et al., 2021). The parabrachial complex is composed of the lateral and medial parabrachial nuclei, that have predominantly ascending projections carrying sensory information, and the Kölliker-Fuse nucleus that primarily targets subcortical structures (Fulwiler and Saper, 1984). Accordingly, of the parabrachial complex it is mainly the Kölliker-Fuse nucleus that influences the switch from inspiration to expiration (Damasceno et al., 2014; Dutschmann et al., 2021; Figure 3B). Neurons of the Kölliker-Fuse nucleus show activity related to specific phases of respiration, with most neurons being active during inspiration; these latter neurons abruptly stop firing at the end of inspiration, marking the inspiration-expiration transition (Dick et al., 1994; Ezure and Tanaka, 2006; Dutschmann et al., 2021). A bilateral block of activity in the Kölliker-Fuse nucleus prolonged the inspiratory activity of the phrenic nerve in an in situ preparation, but did not completely block the termination of inspiratory activity (Dutschmann et al., 2021), which would be in line with a prominent, but not exclusive role of the Kölliker-Fuse nucleus for the termination of inspiration.

The Kölliker-Fuse nucleus receives input from the pre-Bötzinger and Bötzinger complexes (Ezure et al., 2003; Tan et al., 2010; Yang and Feldman, 2018), and from several central chemoreceptor areas: the NTS (Loewy and Burton, 1978; Herbert et al., 1990; McGovern et al., 2015b), retrotrapezoid nucleus (Rosin et al., 2006; Bochorishvili et al., 2012; Silva et al., 2016b), and cerebellar fastigial nucleus (Fujita et al., 2020). In addition, the Kölliker-Fuse nucleus also receives input from the rVRG (Lipski et al., 1994; Yokota et al., 2016), cVRG (Holstege, 1989; Jones et al., 2016), periaqueductal gray (Trevizan-Baú et al., 2021b), spinal trigeminal nucleus (Panneton et al., 2006; Zhang et al., 2018), paratrigeminal nucleus (Saxon and Hopkins, 1998), pedunculopontine tegmental nucleus (PPTg) (Lima et al., 2019b), and vestibular nuclei (Shi et al., 2021). Finally, there are descending inputs from the lateral, dorsomedial and paraventricular hypothalamus (Yokota et al., 2016; Trevizan-Baú et al., 2021a).

Glutamatergic projections directly and indirectly (via the rVRG) target the phrenic nucleus (Ellenberger et al., 1990b; Yokota et al., 2004; Yokota et al., 2007; Song et al., 2012a; Geerling et al., 2017), as well as the ambiguus, hypoglossal and facial nuclei (Núñez-Abades et al., 1990; Yokota et al., 2007; Song et al., 2012a; Yokota et al., 2015; Geerling et al., 2017). The latter connections allow premotor neurons in the Kölliker-Fuse nucleus to constrict valve muscles, reducing outflow during post-inspiration (Dutschmann and Herbert, 2006).

Further excitatory projections target, in addition to the other nuclei of the parabrachial complex (Song et al., 2012a; Geerling et al., 2017), the pre-Bötzinger complex (Yang et al., 2020), PiCo (Oliveira et al., 2021), and lateral parafacial nucleus (Biancardi et al., 2021). Also the cVRG (Gerrits and Holstege, 1996; Song et al., 2012a), reticular formation (Geerling et al., 2017), retrotrapezoid nucleus, NTS, and periaqueductal gray (Fulwiler and Saper, 1984; Song et al., 2012a; Geerling et al., 2017; Trevizan-Baú et al., 2021b) receive glutamatergic input. The caudal part of the Kölliker-Fuse nucleus sends inhibitory projections mainly to the sensory trigeminal nucleus, but also to the dorsomedial hypothalamus (Geerling et al., 2017). Finally, there are projections to the raphe nuclei (Hermann et al., 1997; Peyron et al., 2018), vestibular nuclei (Shi et al., 2021), and cerebellar cortex (Fu et al., 2011).

### Post-inspiratory complex

Recently, a second brain region involved in controlling post-inspiration has been identified in the ventral part of the intermediate reticular formation: the postinspiratory complex, or PiCo, consisting of cholinergic neurons (Anderson et al., 2016; Toor et al., 2019; Oliveira et al., 2021). Isolated in a tissue slice, the PiCo can generate rhythmic activity that peaks during post-inspiration, and thus potentially contributes to a biphasic (pre-Bötzinger complex – PiCo) or triphasic (pre-Bötzinger complex – PiCo – parafacial nucleus) oscillator controlling respiration (Anderson et al., 2016; Anderson and Ramirez, 2017). Optogenetic stimulation in vivo confirmed that PiCo activity can prolong post-inspiration (Oliveira et al., 2021).

The role of the PiCo in controlling post-inspiration has not yet been fully investigated (Hülsmann, 2021; Ashhad et al., 2022). Although this is not a proof against a coordinating role of the PiCo for post-inspiration, systematic recordings in a whole-brainstem preparation found widespread activity during post-inspiration, but more in the pontine region than focused in the area of the PiCo (Dhingra et al., 2020). Furthermore, inhibition of the PiCo did not affect the duration of inspiration, while in the same study the PiCo was shown to be involved in gating swallowing motor patterns to the respiratory system (Toor et al., 2019). In conclusion, it seems likely that the PiCo is involved in the neural control of post-inspiration, but probably more as part on an integrated network than as primary pattern generator.

The PiCo receives strong input from the Kölliker-Fuse nucleus and periaqueductal gray, but there are also substantial connections from the caudal and intermediate NTS and the hypothalamic paraventricular nucleus (Oliveira et al., 2021). Also the pre-Bötzinger complex projects to the PiCo (Yang and Feldman, 2018). As far as we are aware, there are no systematic studies on PiCo efferents, but projections to the pre-Bötzinger complex and retrotrapezoid nucleus have been demonstrated (Lima et al., 2019b; Yang et al., 2020).

### Lateral parafacial nucleus

At the end of the respiratory cycle, the lateral parafacial nucleus can trigger active expiration by recruiting expiratory abdominal muscles via the cVRG (Janczewski and Feldman, 2006; Huckstepp et al., 2015; Silva et al., 2016a; Pisanski and Pagliardini, 2019). In the literature, the term parafacial respiratory group is sometimes used as synonym for the lateral parafacial nucleus, or for the combination of the lateral and the ventral parafacial nucleus, or even for the latter together with the retrotrapezoid nucleus (Onimaru and Homma, 2003; Huckstepp et al., 2015; Pisanski and Pagliardini, 2019; Biancardi et al., 2021).

In embryonic and newborn rodents, the lateral parafacial nucleus is rhythmically active at rest (Onimaru and Homma, 2003; Thoby-Brisson et al., 2009), but this activity wanes during early development (Oku et al., 2007; van der Heijden and Zoghbi, 2020), and in adults the lateral parafacial nucleus is generally silent during eupnea, but rhythmically active during hyperpnea (Pagliardini et al., 2011; Huckstepp et al., 2015; de Britto and Moraes, 2017; Figure 3C–D). The inactivity at rest could be due to tonic inhibition from the medial NTS (Silva et al., 2019). Excitatory input from the commissural NTS, conveying chemosensitive input from the carotid bodies, can activate neurons in the lateral parafacial nucleus during periods with elevated blood CO_2_ levels (Morris et al., 2018; Silva et al., 2019). In addition, the parafacial nucleus receives direct input from chemoreceptors in the adjacent retrotrapezoid nucleus (Zoccal et al., 2018; Biancardi et al., 2021). Furthermore, input comes also from the pre-Bötzinger and Bötzinger complexes, rVRG, Kölliker-Fuse nucleus, reticular formation, caudal raphe, lateral and medial parabrachial nuclei, periaqueductal gray, PPTg and vestibular nuclei (Biancardi et al., 2021).

## Sensory feedback

To adapt the depth of ventilation to the metabolic state, the blood gas balance is continuously monitored. At the same time, sensory systems of the airways and lungs, consisting of thermo-, mechano-, and chemoreceptors, survey ongoing respiration and detect environmental irritants and inflammatory mediators (Sant’Ambrogio et al., 1983; Lee and Yu, 2014). Muscle spindles give feedback on posture and respiratory muscle performance (Nakayama et al., 1998). Altogether, sensory feedback affects ventilatory control, and can trigger respiratory reflexes aimed at maintaining homeostasis upon adverse events. In addition, also hormones can modulate respiratory behavior.

### Chemoreception

Chemoreceptors monitor the partial pressures of O_2_ (pO_2_) and CO_2_ (pCO_2_) in blood and cerebrospinal fluid. Since CO_2_ reacts with water to form HCO_3_^-^ and H^+^, increased pCO_2_ leads to acidosis that can cause adverse effects on tissue structures, and may result in headaches, delirium and eventually coma (Cummins et al., 2020). Regulation of pCO_2_ is therefore, next to that of pO_2_, of great importance, and a major drive for the level of ventilation (Miescher-Rüsch, 1885; Haldane and Priestley, 1905). The concentrations of both gasses are continuously measured by peripheral chemoreceptors in the carotid bodies (Gonzalez et al., 1994; Milsom and Burleson, 2007; Cummins et al., 2020; Ortega-Sáenz and López-Barneo, 2020). The carotid bodies project mainly to the NTS (Claps and Torrealba, 1988; Finley and Katz, 1992; Mifflin, 1992; Zera et al., 2019), but also to the cVRG (Finley and Katz, 1992).

**Figure 4.**
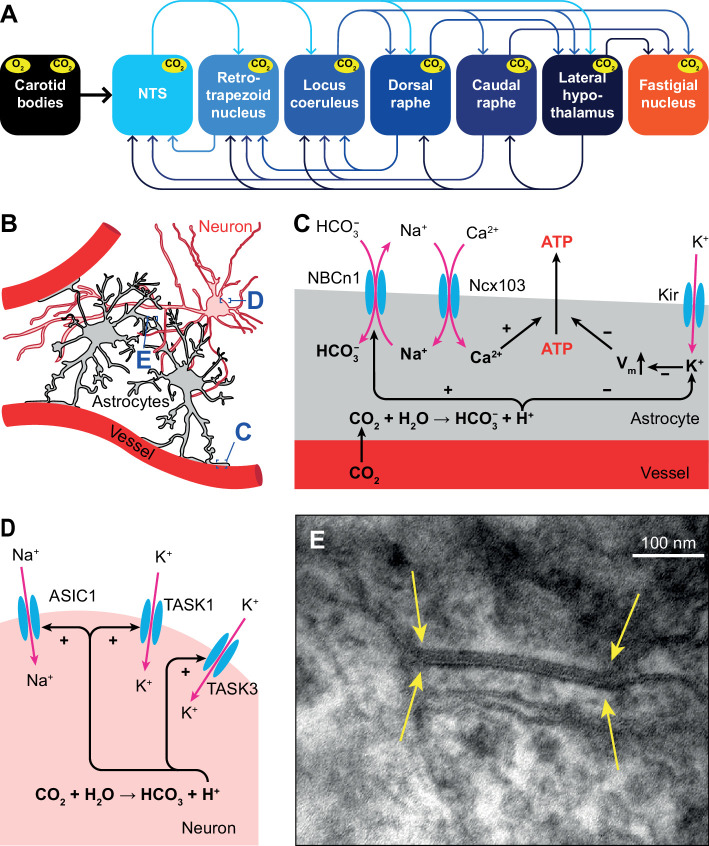
Respiratory chemoreception. (**A**) Connections between the carotid bodies and the central chemoreceptor areas. (**B**) Astrocytes are in direct contact with blood vessels and neurons. (**C**) Chemoreceptor pathways in astrocytes triggering ATP release that can activate nearby neurons. (**D**) Chemoreceptor pathways in neurons, based on activation of Na^+^ channels and inward-rectifier K^+^ channels. (**E**) Gap junctions between glial cells, as shown here in the cerebellar cortex of a mouse (yellow arrows), can contribute to central chemoreception. In particular, the conductivity of gap junctions composed of Cx26 depends on pH. Previously unpublished electron microscopic image from our lab.

Acidosis can also be caused by inflammation, ischemia or defective acid containment. Consequently, acid sensing is not restricted to respiratory control and has evolved as an important property of neurons with unmyelinated and thinly myelinated fibers (Canning and Spina, 2009). Only those areas that sense pH changes and directly affect ventilation are considered to be central chemoreceptor areas. In this respect, most attention has been given to the retrotrapezoid nucleus, but also the NTS, locus coeruleus, raphe nuclei, lateral hypothalamus, and cerebellar fastigial nucleus are central chemoreceptor areas (Coates et al., 1993; Nattie, 1999; Nattie and Li, 2002; Xu and Frazier, 2002; Putnam et al., 2004; Guyenet et al., 2008; Dean and Putnam, 2010; Li et al., 2013; Figure 4A).

### Central chemoreceptors

Several molecular mechanisms for central chemoreception have been proposed (Gourine and Dale, 2022). First, in astrocytes, increased levels of HCO_3_^-^ can activate the electroneutral Na^+^/HCO_3_^-^ co-transporter NBCn1 (*SLC4A7*), causing Na^+^ influx that in turn activates the astrocytic Na^+^/Ca^2+^ exchanger Ncx103 (*SLC8A1-3;* Turovsky et al., 2016). The resulting increase in intracellular Ca^2+^ triggers ATP release (Gourine et al., 2005; Gourine et al., 2010). Purinergic receptors on nearby neurons are activated by ATP, leading to neuronal excitation (Gourine et al., 2005; Figure 4B–C).

Second, inward-rectifier K^+^ (K_ir_) channels can be inhibited by a decrease in pH, and trigger depolarization and consequently ATP release by astrocytes (Wenker et al., 2010; Figure 4C).

A third mechanism, one that is independent of pH changes, involves the ability of CO_2_ to induce conformational changes in the connexin-hemichannel Cx26 located in gap junctions between glial cells, rendering Cx26 permeable to ATP (Bevans and Harris, 1999; Huckstepp et al., 2010; Meigh et al., 2013; van de Wiel et al., 2020; Figure 4E). Later studies revealed the interaction between CO_2_ and Cx26 to be more complicated, and probably context-dependent (Nijjar et al., 2021).

In addition to glial-mediated chemoreception, also neurons express acid-sensitive ion channels (ASICs) and receptors (Canning and Spina, 2009). Among these are some members of the family of inwardly rectifying K_2P_ channels (Lesage and Barhanin, 2011; Sepúlveda et al., 2015), such as TASK1 and TASK3 that are co-expressed with ASIC1 in the ventrolateral medulla, and contribute to central chemoreception in rats (Wang et al., 2018). Another family member is the TASK-2 K^+^ leak channel, whose absence leads to impaired ventilatory responses to hypercapnia (Gestreau et al., 2010; Bayliss et al., 2015; Figure 4D).

The *KCNA1* gene encodes the α subunit of K_v_1.1 voltage-gated potassium channels that show particularly strong expression in hippocampus, cerebellum, neocortex and peripheral nerves (D’Adamo et al., 2014; Ovsepian et al., 2016). Mutations in the *KCNA1* gene can lead to the development of episodic ataxia type 1 (EA1), an autosomal dominant disorder with multiple symptoms, most prominently episodes of cerebellar ataxia and myokymia (Browne et al., 1994; Paulhus et al., 2020). These episodes are often triggered by physical and emotional stress, which could be related to a defect in respiratory chemoreception (Kline et al., 2005). Furthermore, mutations in K_v_1.1 channels have been associated with epilepsy, and *Kcna1*-deficient mice are considered to be a model of sudden unexpected death in epilepsy (SUDEP), while also showing progressive respiratory dysfunction (Simeone et al., 2018; D’Adamo et al., 2020; Paulhus et al., 2020). As respiratory dysfunction is hypothesized to be a primary risk factor for susceptibility to the cardiorespiratory dysfunction in epilepsy, this could reveal a new role for *KCNA1* channelopathies in the regulation of basal respiratory physiology (Dhaibar et al., 2019).

### Vagal sensory input

The lungs and airways, in particular the larynx, are lined with sensory receptors associated with the nervus vagus (Mortola et al., 1975; Lee and Yu, 2014; Mazzone and Undem, 2016; Kupari et al., 2019). Flow receptors sense air temperature, which is typically colder for inhaled than for exhaled air (Sant’Ambrogio et al., 1983), and drive receptors measure the laryngeal wall pressure that correlates with its conductivity (Horner, 2012). Flow and drive receptors are active during each breath (Sant’Ambrogio et al., 1983), while other mechano- and chemoreceptors are only activated during adverse conditions, such as an obstruction, the presence of irritants, or increased CO_2_ concentrations (Lee and Yu, 2014). Sensory information from the distal airways is transferred via the nodose ganglion to the NTS, while that of the proximal airways goes predominantly via the jugular ganglion to the paratrigeminal nucleus (McGovern et al., 2015a; McGovern et al., 2015b; Kim et al., 2020).

Vagal stretch receptors, associated with Aβ fibers, can be subdivided into slowly and rapidly adapting receptors: SARs and RARs, respectively. SARs sense inflation and can stay activated for sustained periods, while RARs are more sensitive to acute changes in pressure (Davenport et al., 1981; Kaufman et al., 1982; Mazzone and Undem, 2016). Although all RARs rapidly adapt to a persistent mechanical stimulus, a subset of them, the so-called irritant receptors, can remain activated by specific chemicals for a prolonged period (Sant’Ambrogio, 1982). Environmental irritants and inflammatory mediators can trigger activity of unmyelinated C fibers, while localized mechanical stimuli and acid can activate thinly myelinated Aδ fibers that are also known as cough receptors, and that are located in the airway epithelium and mucosa (Coleridge and Coleridge, 1984; Canning et al., 2004; Mazzone et al., 2009; Grace et al., 2012; Canning et al., 2014; Mazzone and Undem, 2016).

### Cough and expiration reflexes

Coughing is a vital action to clear the airways. Coughing involves first inspiration, then obstruction of airways to build up pressure, and subsequent explosive expulsion of air. Ineffective cough reflexes, for example in patients with dementia, can lead to lethal aspiration pneumonia (Widdicombe, 1995; Mutolo, 2017; Sykes and Morice, 2021). Inversely, the relatively common condition of chronic cough, affecting more than 5% of the adult population, is thought to be caused by hypersensitivity to airway stimulation (Morice et al., 2020).

The cough reflex is typically triggered by stimulation of the cough receptors and possibly also by activation of C fibers in the airways (Ludlow, 2015; Mutolo, 2017). Mechanical stimulation of the vocal cords can trigger the expiration reflex, which resembles cough without the initial inspiration (Korpas and Jakus, 2000; Sant’Ambrogio and Widdicombe, 2001; Tatar et al., 2008; Ludlow, 2015).

**Figure 5.**
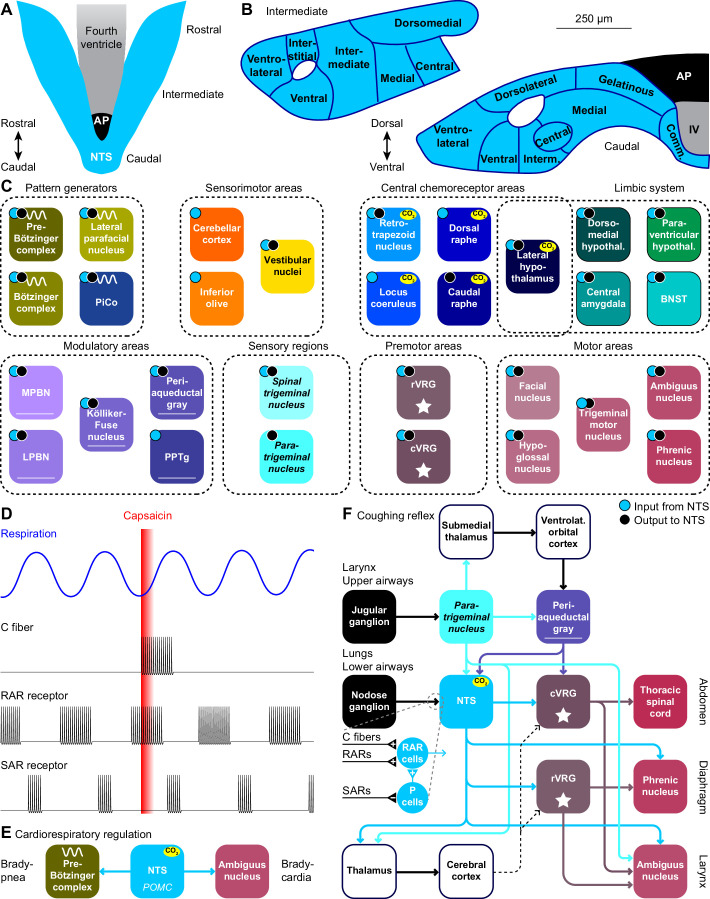
The nucleus of the solitary tract and vagal afferents. Schematic drawings of the nucleus of the solitary tract (NTS) in relation to the area postrema (AP) and the fourth ventricle (IV). Dorsal (**A**) and coronal views at intermediate and caudal levels (**B**). (**C**) Brain regions that project to, or get input from the NTS. With most areas, bidirectional connections exist (cyan dots: areas innervated by NTS neurons; black dots: areas with neurons innervating the NTS). (**D**) Heterogeneity in vagal afferents. Capsaicin can, as a pulmonary irritant, evoke activity of C fibers, but not of rapidly or slowly adapting receptors (RAR and SAR, respectively). The latter two show phasic activity during regular breathing. Schematic drawing of action potential firing based on data presented in Ho et al., 2001. (**E**) Pro-opiomelanocortin-expressing (POMC) neurons of the NTS project to the pre-Bötzinger complex and to cardiac vagal motor neurons in the ambiguus nucleus. Via these pathways, they can reduce inspiration and cardiac function, respectively. (**F**) Coughing can be triggered by sensing an irritant via vagal projections from the larynx or upper airways via the jugular ganglion to the paratrigeminal nucleus, or from the lungs or lower airways via the nodose ganglion to the NTS. Distinct types of vagal fibers differentially affect RAR relay neurons, with SARs inhibiting pump neurons (P cells). The motor neurons of expiratory muscles are directly and indirectly activated from the paratrigeminal nucleus and the NTS. A specific cortical circuit for inhibiting reflexive coughing involving the submedial thalamus and the ventrolateral orbital cortex has been described, next to more general thalamo-cortical pathways that can modulate coughing. The latter pathways can use different connections to premotor nuclei (indicated with dotted lines).

Stimulation of irritant receptors in the upper airways can evoke a cough reflex via activation of the paratrigeminal nucleus, and this reflex can be suppressed by the submedial thalamic nucleus and the upstream ventrolateral orbital cortex (Mazzone et al., 2020). In the periaqueductal gray, the excitatory drive from the paratrigeminal nucleus and the inhibitory input from the ventrolateral orbital cortex come together (McGovern et al., 2015b). The periaqueductal gray is hence an important intermediate between the forebrain and the cVRG (Holstege, 1989; Subramanian et al., 2008; Chen et al., 2022). The cVRG itself is essential for the cough reflex (Mutolo, 2017; Cinelli et al., 2020), as it can activate both expiratory motor neurons in the thoracic spinal cord, controlling abdominal muscles, and in the ambiguus nucleus, controlling laryngeal muscles (Figure 5F).

Stimulation of the lower airways or the lungs activates RAR relay neurons in the NTS. These neurons receive also input from various other sources that can contribute to suppression or facilitation of coughing (Mutolo, 2017). Thalamocortical loops can further modulate coughing and exert voluntary control (Ando et al., 2016). Like the paratrigeminal nucleus, the NTS innervates the cVRG (Gerrits and Holstege, 1996). In addition, the NTS affects also the phrenic nucleus, directly as well as indirectly via the rVRG (Ezure and Tanaka, 1996; Wu et al., 2017). These connections are complemented with direct output to the ambiguus nucleus from both the paratrigeminal nucleus and the NTS (Caous et al., 2001; de Sousa Buck et al., 2001; Kawai, 2018; Figure 5F).

### Sneeze reflex

In the nasal mucosa, irritant receptors expressing Trpv1, a capsaicin-sensitive cation ion channel, that can be activated by histamine H1R receptors (Shim et al., 2007), are present on thin sensory fibers of the ethmoidal nerve that terminates in the sneeze-evoking region (Lucier and Egizii, 1986; Nonaka et al., 1990; Seijo-Martínez et al., 2006; Li et al., 2021). The sneeze-evoking region is a dedicated part of the spinal trigeminal nucleus that projects to the cVRG (Li et al., 2021).

### Postural feedback

The external and internal intercostal muscles assist with, respectively, expansion and contraction of the rib-cage during breathing (Figure 1A–E). In addition, the intercostal muscles exert postural control, and they combine their respiratory and postural activity in a superposed manner (Rimmer et al., 1995). Single intercostal 1a afferents project to the region of Clarke’s column, to the intercostal motor nucleus, and to the intermediate regions, conveying sensory feedback originating from muscle spindles in the intercostal muscles (Nakayama et al., 1998). From Clarke’s column, information is projected to the cerebellar cortex via the spinocerebellar tract, originating from two types of respiration-related neurons in the lower thoracic segments (T9-T12). This spinocerebellar tract contains uncrossed as well as crossed ascending axons that play different roles in transmitting signals between the spinal cord and the cerebellum (Tanaka and Hirai, 1994). Rhythmic activity in uncrossed spinocerebellar tract neurons, located in and around Clarke’s column, reflects afferent activity from the chest wall, whereas that of crossed neurons, located in laminae VII and VIII, reflect descending influence from the respiratory centers with or without peripheral influences (Matsushita et al., 1979; Tanaka and Hirai, 1994). It has been suggested that the cerebellum uses the posture-related information of the thoracic spinocerebellar tract neurons to adjust posture or coordination of whole body movements (Tanaka and Hirai, 1994).

### Hormonal regulation of respiration

Several hormones can affect development and metabolism, and thus have an indirect effect on respiration. Thyroid hormones, for instance, are critical for the development of the respiratory system, and their dysfunction can lead to respiratory failure, including the respiratory distress syndrome (Pei et al., 2011; Rousseau et al., 2021). Hormones with a specific role in respiratory control are discussed below.

### Sex hormones

Progesterone, estradiol and testosterone can all affect respiratory parameters (White et al., 1985), and these effects could help explain differences in respiratory behavior between males and females, as well as during different life stages (Gargaglioni et al., 2019; LoMauro and Aliverti, 2021). Indeed, sex hormone receptors are widely distributed in the brain, including central chemoreceptor areas (Gargaglioni et al., 2019; LoMauro and Aliverti, 2021), certain respiratory motor neurons, such as the hypoglossal and phrenic nuclei (Behan and Thomas, 2005), and cerebellar Purkinje cells (Perez-Pouchoulen et al., 2016). Progesterone levels correlate with the muscle tone of the genioglossus muscle, and thus with upper airway rigidity, which could play a role in the reduced occurrence of obstructive sleep apnea in females when compared to males (Popovic and White, 1998).

### Leptin

Leptin is primarily secreted by adipocytes. An increase in adipose tissue therefore leads to more leptin secretion, which results in increased breathing activity (Chang et al., 2013; Bassi et al., 2016; Gauda et al., 2020). Leptin can activate a specific subset of excitatory neurons in the NTS projecting to respiratory premotor neurons in the rVRG, as well as to the dorsomedial hypothalamus (Do et al., 2020). Next to the direct projection from the NTS to the rVRG, an indirect pathway via the lateral parabrachial nucleus to the pre-Bötzinger complex is also likely to contribute to the impact of leptin on respiratory frequency (Yu et al., 2022). The dorsomedial hypothalamus, which itself also contains leptin receptors, can contribute to upper airway control during respiration (Yao et al., 2016). The impact of the leptin-mediated pathways may be compromised in obesity, as leptin resistance can contribute to the development of obesity hypoventilation syndrome and central sleep apnea (O’Donnell et al., 2000; Yao et al., 2016; Framnes and Arble, 2018).

Full-length leptin receptors (*LEPRB*) are not restricted to the NTS and dorsomedial hypothalamus, but can also be found in the carotid bodies, neocortex, substantia nigra and cerebellum (Guan et al., 1997; Gavello et al., 2016). Although it is not clear whether these all affect respiratory control, it has been shown that activation of leptin receptors in the carotid bodies can affect breathing and induce ventilatory response to hypoxia (Caballero-Eraso et al., 2019).

### Hyperventilation

Not only increased metabolic activity, but also emotional arousal can cause an increase in the level of ventilation. While such a stress-induced reaction makes sense as preparation for a fight or flight reaction, it can derail during a panic attack (Suess et al., 1980; Meuret et al., 2017). In the absence of increased metabolic demands, hyperventilation induces a decrease in pCO_2_, which increases blood pH (Gardner, 1996). Hyperventilation can be associated with several symptoms of panic, including shortness of breath, heart racing, dizziness, and fear of dying (Gardner, 1996; Meuret et al., 2017). Although hyperventilation is typically triggered by stress, anxiety or panic, in rare cases it can also have a neurological cause, and central neurogenic hyperventilation is often associated with pontine damage (Plum and Swanson, 1959; Tarulli et al., 2005).

## Central chemoreceptor areas

### Retrotrapezoid nucleus

The retrotrapezoid nucleus is considered as the main central chemoreceptor area, and inhibition of its activity reduces the ventilatory response to hypercapnia (Mulkey et al., 2004; Marina et al., 2010; Burke et al., 2015; Ruffault et al., 2015), while optogenetic stimulation of the retrotrapezoid nucleus can increase the breathing rate by reducing the duration of expiration (Abbott et al., 2009; Abbott et al., 2011; Burke et al., 2015; Souza et al., 2020). Cholinergic input, probably from the PPTg and the PiCo, can increase the activity of chemoreceptors (Sobrinho et al., 2016; Lima et al., 2019b), while serotonergic input from the caudal and dorsal raphe can enhance the chemosensitive response in the retrotrapezoid nucleus (Rosin et al., 2006; Brust et al., 2014; Wu et al., 2019; Leirão et al., 2021).

The retrotrapezoid nucleus consists in mice of around 700 Phox2b-positive cells located ventrolateral to the facial nucleus. The locations of these neurons partially overlap with those of the lateral parafacial nucleus (Smith et al., 1989; Onimaru and Homma, 2003; Ramanantsoa et al., 2011; Shi et al., 2017). The retrotrapezoid nucleus houses also around 200 biochemically and morphologically different neurons that lack CO_2_-sensitivity, and that could be related to sighing via their direct projection to the pre-Bötzinger complex (Li et al., 2016; Shi et al., 2017).

Next to pCO_2_, also sensory input relayed via the caudal and commissural parts of the NTS (Rosin et al., 2006) affects the activity of the retrotrapezoid nucleus. In addition, direct inputs come from the pre-Bötzinger complex (Tan et al., 2010; Yang and Feldman, 2018), Kölliker-Fuse nucleus, and lateral and medial parabrachial nuclei (Rosin et al., 2006; Song et al., 2012a; Lima et al., 2019b), rVRG and cVRG (Rosin et al., 2006; Jones et al., 2016), lateral and paraventricular hypothalamus (Rosin et al., 2006; Geerling et al., 2010), and central amygdala and periaqueductal gray (Rosin et al., 2006).

The output of the retrotrapezoid is glutamatergic and affects respiration directly via projections to the pre-Bötzinger complex, rVRG and cVRG, as well as to the cervical and thoracic spinal cord (Rosin et al., 2006; Bochorishvili et al., 2012; Li et al., 2016; Silva et al., 2016a), and indirectly via the Bötzinger complex, Kölliker-Fuse nucleus, lateral parafacial nucleus, NTS, and lateral and medial parabrachial nucleus (Rosin et al., 2006; Abbott et al., 2009; Bochorishvili et al., 2012; Silva et al., 2016a).

### Nucleus of the solitary tract

The NTS houses respiratory chemoreceptors (Coates et al., 1993; Nattie and Li, 2002; Nattie and Li, 2008; Fu et al., 2017), and is the prime recipient of visceral input. Especially the caudal part of the NTS receives direct input from pulmonary and cardiovascular baro-, chemo- and stretch receptors, as well as from chemoreceptors in the carotid bodies (Miura and Reis, 1972; Sant’Ambrogio, 1982; Finley and Katz, 1992; Mifflin, 1992; Lee and Yu, 2014; Zoccal et al., 2014; Mazzone and Undem, 2016; Umans and Liberles, 2018; Zera et al., 2019; Suarez-Roca et al., 2021; Figure 5A–B). These inputs allow the NTS to contribute to metabolic homeostasis, affecting not only cardiorespiratory function, but also food intake and digestion (Rinaman, 2010; Zoccal et al., 2014). The NTS is not essential for inspiration, but NTS dysfunction impairs the response to hypercapnia (Berger and Cooney, 1982; Speck and Feldman, 1982; Nattie and Li, 2008; Dean and Putnam, 2010), which could underlie congenital central hypoventilation syndrome (Fu et al., 2017). The ventrolateral NTS is also known as the dorsal respiratory group (Berger, 1977).

The NTS contains multiple types of respiratory neurons, some relate to inspiration, others to expiration or are phase-independent (Backman et al., 1984; Ezure and Tanaka, 2000; Ho et al., 2001; Kubin et al., 2006; Subramanian et al., 2007; Figure 5D). Among these are neurons, located mainly in the commissural and medial NTS, that respond to pulmonary irritant receptors, including RARs and C-fibers mediating cough reflexes, and that are suppressed by the pump neurons (P-cells; Berger, 1977; Kubin et al., 2006; Canning et al., 2014; Mutolo, 2017; Farrell et al., 2020).

P-cells of the ventrolateral and other parts of the caudal NTS respond to SARs reporting airway stretch (Kubin et al., 2006; Zoccal et al., 2014; Mazzone and Undem, 2016; Umans and Liberles, 2018). The firing rates of the P-cells relate to lung volume, possibly modulated by the respiratory rhythm (Berger, 1977; Davies et al., 1987; Bonham and McCrimmon, 1990; Miyazaki et al., 1999). Activation of these P-cells results in an inhibition of the rVRG (Ezure and Tanaka, 1996; Zheng et al., 1998) and phrenic motor nucleus (Fedorko et al., 1983; Ellenberger et al., 1990b; Boulenguez et al., 2007; Lois et al., 2009).

In addition, the NTS is involved in several other mechanisms controlling respiration. These entail a strong and direct projection to the phrenic motor nucleus (Loewy and Burton, 1978; Fedorko et al., 1983; Rikard-Bell et al., 1984; Dobbins and Feldman, 1994; Boulenguez et al., 2007; Lois et al., 2009). Furthermore, P-cells of the intermediate NTS mediate the Hering-Breuer inspiratory reflex that protects the lungs against overinflation (Breuer, 1868; Berger, 1977; Bonham and McCrimmon, 1990; Kubin et al., 2006; Lee and Yu, 2014; Chang et al., 2015; Nonomura et al., 2017; Umans and Liberles, 2018).

In addition to the extensive visceral inputs, the caudal NTS also receives central input, in particular from other regions of the NTS, lateral and paraventricular hypothalamus and central amygdala (Geerling et al., 2010; Ruyle et al., 2019; Gasparini et al., 2020), but also from the pre-Bötzinger complex (Tan et al., 2010; Koshiya et al., 2014; Yang and Feldman, 2018), Bötzinger complex (Merrill et al., 1983; Fedorko and Merrill, 1984; Livingston and Berger, 1989; Ezure et al., 2003), rVRG (Yamada et al., 1988; Ellenberger et al., 1990a; Zheng et al., 1998), Kölliker-Fuse nucleus (Fulwiler and Saper, 1984; Song et al., 2012a; Geerling et al., 2017), retrotrapezoid nucleus (Rosin et al., 2006; Bochorishvili et al., 2012), caudal raphe (Brust et al., 2014), bed nucleus of the stria terminalis (Gasparini et al., 2020), lateral and medial parabrachial nuclei (Saper and Loewy, 1980; Herbert et al., 1990; Bianchi et al., 1998), periaqueductal gray (Chen et al., 2022), spinal trigeminal nucleus (Panneton et al., 2006), paratrigeminal nucleus (Saxon and Hopkins, 1998; de Sousa Buck et al., 2001; McGovern et al., 2015b; Driessen et al., 2018), as well as from insular and infralimbic areas of the cerebral cortex (Gasparini et al., 2020; Figure 5C). Evidence for significant projections from the cerebellum to the NTS is currently lacking (Teune et al., 2000; Gasparini et al., 2020).

As mentioned above, strong and direct projections from the NTS to the phrenic nucleus have been reported, while the NTS targets also the upper airway motor nuclei (Loewy and Burton, 1978; Norgren, 1978; Beckstead et al., 1980; Núñez-Abades et al., 1990; Hayakawa et al., 2000; Kawai, 2018; Guo et al., 2020). Other target areas are the pre-Bötzinger complex (Yang et al., 2020), Bötzinger complex (Gang et al., 1995), PiCo (Oliveira et al., 2021), lateral parafacial nucleus (Biancardi et al., 2021), cVRG (Loewy and Burton, 1978; Beckstead et al., 1980; Gerrits and Holstege, 1996), retrotrapezoid nucleus (Rosin et al., 2006; Lima et al., 2019b), locus coeruleus (McGovern et al., 2015b; Kawai, 2018), dorsal raphe (Peyron et al., 2018), lateral, paraventricular and dorsomedial hypothalamus (King et al., 2012; McGovern et al., 2015b; Kawai, 2018), bed nucleus of the stria terminalis and central amygdala (Shin et al., 2008; Bienkowski and Rinaman, 2013; McGovern et al., 2015b; Ni et al., 2016; Kawai, 2018), lateral and medial parabrachial nuclei (Loewy and Burton, 1978; Beckstead et al., 1980; Herbert et al., 1990; McGovern et al., 2015b; Hashimoto et al., 2018; Kawai, 2018; Yu et al., 2022), periaqueductal gray (Herbert and Saper, 1992; Kawai, 2018), spinal trigeminal nucleus (Loewy and Burton, 1978; McGovern et al., 2015b), pedunculopontine tegmental nucleus (PPTg) (Steininger et al., 1992), cerebellum (Batini et al., 1978; Somana and Walberg, 1979b; Saigal et al., 1980b; Fu et al., 2011), and inferior olive (Loewy and Burton, 1978; McGovern et al., 2015b; Figure 5C).

### Locus coeruleus

The locus coeruleus has broad impact on brain activity, affecting among others attention, motivation, memory, and the level of arousal through its widespread network of noradrenergic fibers (Schwarz et al., 2015; Breton-Provencher and Sur, 2019; Chandler et al., 2019; Poe et al., 2020). The locus coeruleus can also mediate sensory-evoked awakenings from sleep (Hayat et al., 2020) and has been proposed to be a key center in coupling brain activity with the respiratory cycle (Melnychuk et al., 2021).

Hypercapnia leads to increased activity in the locus coeruleus, which is an evolutionary conserved phenomenon observed in amphibians and mammals (Elam et al., 1981; Pineda and Aghajanian, 1997; Biancardi et al., 2008; Santin and Hartzler, 2013; Quintero et al., 2017). This increase in neural activity can lead to stronger basal ventilation (Hilaire et al., 2004; Liu et al., 2021b), although this effect was not observed in all studies (Gargaglioni et al., 2010). The impact of the locus coeruleus may therefore depend on behavioral or experimental conditions.

Stimulation of the locus coeruleus can modulate activity in the pre-Bötzinger complex via a direct projection (Liu et al., 2021b). The locus coeruleus also targets many other brain regions with three diverging pathways, with individual locus coeruleus neurons projecting to functionally related areas (Szabadi, 2013; Schwarz et al., 2015; Poe et al., 2020). The ascending pathway targets the thalamus and cerebral cortex, but also the paraventricular and lateral hypothalamus, periaqueductal gray, dorsal and caudal raphe nuclei, bed nucleus of the stria terminalis, and central amygdala (Jones and Moore, 1977; Jones and Yang, 1985; Hermann et al., 1997; Ni et al., 2016; Borodovitsyna et al., 2020). Other targets include the nuclei of the mesodiencephalic junction (MDJ) (Jones and Yang, 1985). The cerebellar pathway targets both the cerebellar nuclei and cortex (Olson and Fuxe, 1971; Saigal et al., 1980a; Nagai et al., 1981; Room et al., 1981; Loughlin et al., 1986; Dietrichs, 1988; Fu et al., 2011). The third, descending pathway targets the brainstem and spinal cord, including the ambiguus, hypoglossal and facial nuclei, dorsal raphe, and the pedunculopontine tegmental nucleus (Jones and Yang, 1985). The spinal projections are, as typical for locus coeruleus projections, widespread and involve also the phrenic nucleus, without showing a specific concentration of terminals in the latter (Bruinstroop et al., 2012).

In turn, the locus coeruleus receives widespread but relatively sparse projections from the cerebral cortex, and denser projections from the hypothalamus, central amygdala, bed nucleus of the stria terminalis, raphe nuclei, Kölliker-Fuse nucleus and adjacent parabrachial nuclei, and periaqueductal gray (Luppi et al., 1995; Schwarz et al., 2015). There is also input from the pre-Bötzinger complex (Yackle et al., 2017), NTS (McGovern et al., 2015b; Kawai, 2018), PPTg (Woolf and Butcher, 1989), and cerebellar nuclei, in addition to numerous direct projections from cerebellar Purkinje cells (Schwarz et al., 2015).

### Raphe nuclei

**Figure 6.**
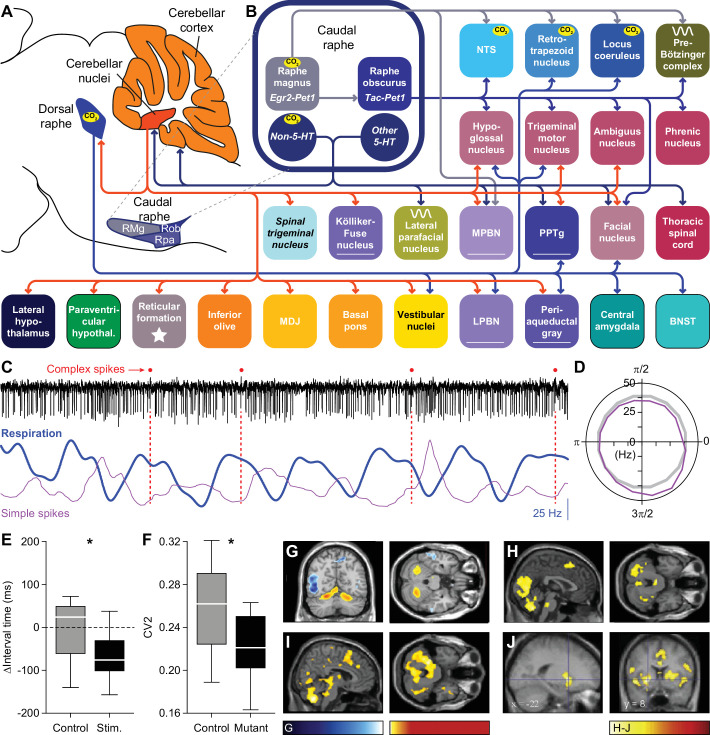
Raphe nuclei and cerebellum. (**A**) The caudal raphe consists of the raphe magnus (RMg), the raphe obscurus (Rob) and the raphe pallidus (Rpa). The cerebellum consists of the cerebellar cortex and the cerebellar nuclei. (**B**) Different populations of raphe neurons can have different projection patterns. For example, Egr2-positive serotonergic neurons of RMg have intrinsic chemoreceptor properties and project mainly to other central chemoreceptor areas. The downstream Tac-positive serotonergic neurons of Rob lack intrinsic chemoreceptor properties and project predominantly to respiratory motor neurons. Other neurons of the caudal raphe, whether serotonergic or non-serotonergic, extend the caudal raphe projections to further respiratory regions. These projections partially overlap with those of the dorsal raphe. Indicated are also the projections from the cerebellar nuclei. Note that also the locus coeruleus receives cerebellar input via direct Purkinje cells projections. (**C**) At rest, Purkinje cells of the lateral cerebellum can show modulation of their complex spike and simple spike frequency in relation to the respiratory cycle. This is illustrated with a representative electrophysiological recording of a Purkinje cell in an awake mouse. Complex spikes are noted with a red dot on top of the trace. In this example, they occur preferably just after the onset of expiration (downward phase of the blue trace). Simple spike firing is increased during the last phase of expiration, as illustrated by their instantaneous frequency (magenta curve). The duration of this fragment is 3.6 s. (**D**) For this Purkinje cell, the simple spike frequency is upregulated at the last phase of respiration, compared to the expected frequency (grey circle). 0=start inspiration. (**E**) Optogenetic stimulation of Purkinje cells in the lateral cerebellum can shorten the interval until the next inspiration. (**F**) In the absence of excitatory output of the cerebellar nuclei, in the Atoh1-En1/2 mouse model for cerebellar neuropathology, the respiratory cycle is more regular than in control mice, as quantified by the local coefficient of variation (CV2). fMRI scans indicate that specific brain regions, including the cerebellum, are activated during respiratory challenges: (**G**) Increasing respiratory resistance. (**H**) Hypoxia (13% oxygen for 1 min). (**I**) Actively slowed breathing. (**J**) During breath holding, the cerebellum is not activated. * p<0.05 Scaling of activity levels in G: –6.7 to –3.1 (blue colors) and +3.1 to+8.5 (red colors); in H-J: 0–15. Panels C and D are modified from Figure 1 from Romano et al., 2020, E from Figure 6 from Romano et al., 2020, F from Figure 4 from Taylor et al., 2022, and H and I from Figure 4 from Critchley et al., 2015.

The raphe nuclei consist of clusters of neurons along the midline of the midbrain, pons and medulla. Of these, the dorsal and caudal raphe both house CO_2_ chemoreceptors, and together form the main source of serotonin in the respiratory system (Wang et al., 2001; Severson et al., 2003; Teran et al., 2014; Figure 6A). Disturbances in serotonin release have been linked to respiratory dysfunction in Prader-Willi syndrome (Matarazzo et al., 2017) as well as to SIDS (Paterson et al., 2006; Kinney and Haynes, 2019). The risk of the latter may be increased by prenatal exposure to nicotine that can later cause impairments in serotonin release during hypercapnia (Avraam et al., 2020).

The dorsal raphe nucleus mediates the CO_2_ arousal reflex via its projection to the lateral parabrachial nucleus (Petrov et al., 1992; Smith et al., 2018; Kaur et al., 2020). Other projections, sometimes involving collateral fibers, target predominantly forebrain regions, including the lateral hypothalamic nucleus, bed nucleus of the stria terminalis, central amygdala, and vestibular nuclei (Vertes, 1991; Petrov et al., 1992; Halberstadt and Balaban, 2006; Vasudeva et al., 2011; McDevitt et al., 2014). Descending projections target the hypoglossal, trigeminal and facial motor nuclei (Li et al., 1993; Guo et al., 2020), retrotrapezoid nucleus (Rosin et al., 2006), locus coeruleus (Luppi et al., 1995; Schwarz et al., 2015), periaqueductal gray (Vertes, 1991), and PPTg (Vertes, 1991; Steininger et al., 1992; Figure 6B).

The dorsal raphe nucleus receives input from many areas, with relatively dense projections originating in the cerebral cortex, lateral, paraventricular and dorsomedial hypothalamus, bed nucleus of the stria terminalis, central amygdala, periaqueductal gray, lateral and medial parabrachial nuclei, Kölliker-Fuse nucleus, NTS, locus coeruleus, and vestibular nuclei (Jones and Yang, 1985; Peyron et al., 1998a; Pollak Dorocic et al., 2014; Weissbourd et al., 2014; Peyron et al., 2018; Shi et al., 2021). In addition, also the cerebellar nuclei project to the dorsal raphe nucleus, but this projection seems to be relatively sparse (Teune et al., 2000; Weissbourd et al., 2014).

The caudal, or medullary, raphe nuclei can also directly affect respiration (Lalley, 1986; Ptak et al., 2009; Depuy et al., 2011; Sabino et al., 2021). The caudal raphe nuclei exist of the raphe magnus, raphe obscurus, and raphe pallidus, and contain serotonergic, non-serotonergic and mixed neurons (Pilowsky, 2014; Figure 6A). Accordingly, neural responses of neurons in the caudal raphe are heterogeneous and hypercapnic acidosis can activate some, and inhibit other neurons of the caudal raphe (Richerson, 1995; Wang et al., 1998), the former category comprising serotonergic neurons, the latter not (Wang et al., 2001; Taylor et al., 2005).

*Egr2-Pet1*-expressing serotonergic neurons of the raphe magnus mediate the respiratory CO_2_ chemoreflex (Brust et al., 2014). These chemoreceptor neurons target predominantly other chemoreceptor areas in the brainstem: the retrotrapezoid nucleus, NTS, locus coeruleus, pre-Bötzinger complex and medial parabrachial nucleus (Brust et al., 2014). *Tac1-Pet1*-expressing serotonergic neurons of the raphe obscurus do not express chemoreceptor properties themselves, but are most likely downstream of *Egr2-Pet1* neurons and preferentially innervate motor nuclei, including the phrenic, facial, trigeminal, hypoglossal, and ambiguus nucleus, but also the pre-Bötzinger complex and NTS (Hennessy et al., 2017).

### Lateral hypothalamus

Orexinergic neurons of the lateral hypothalamus are the only neurons of the diencephalon with central chemoreceptor properties (Williams et al., 2007; Song et al., 2012b; Li et al., 2013; Fukushi et al., 2019; Wang et al., 2021). Orexins, neuropeptides exclusively produced by the lateral and posterior hypothalamus, are distributed widely throughout the brain and promote wakefulness (de Lecea et al., 1998; Peyron et al., 1998b; Hagan et al., 1999; Adamantidis et al., 2007; Berteotti et al., 2021). The activity of orexinergic neurons is largely restricted to the awake state (Mileykovskiy et al., 2005), and dysfunction of the orexinergic system can cause narcolepsy (Thannickal et al., 2000; Bassetti et al., 2019; Berteotti et al., 2021).

Orexins can also mediate respiratory chemoreflex responses and increase the tidal volume (Young et al., 2005; Zhang et al., 2005; Deng et al., 2007). To this end, orexinergic fibers innervate the phrenic (Young et al., 2005) and hypoglossal nuclei (Fung et al., 2001; Guo et al., 2020). Other targets include the pre-Bötzinger complex (Young et al., 2005; Yang et al., 2020; Trevizan-Baú et al., 2021a), Kölliker-Fuse nucleus (Peyron et al., 1998b; Yokota et al., 2016; Trevizan-Baú et al., 2021a), retrotrapezoid nucleus (Rosin et al., 2006), NTS (Peyron et al., 1998b; Gasparini et al., 2020), locus coeruleus (Luppi et al., 1995; Peyron et al., 1998b; Hagan et al., 1999; Schwarz et al., 2015), dorsal and caudal raphe nuclei (Lee et al., 2003; Lee et al., 2005; Ogawa et al., 2014; Weissbourd et al., 2014), bed nucleus of the stria terminalis (Shin et al., 2008; Ni et al., 2016), central amygdala (Peyron et al., 1998b; Fu et al., 2020), periaqueductal gray (Peyron et al., 1998b; Trevizan-Baú et al., 2021a), and PPTg (Semba and Fibiger, 1992; Steininger et al., 1992). There is also widespread innervation of the cerebellar cortex and nuclei, in particular of the fastigial nucleus (Dietrichs, 1984; Ciriello and Caverson, 2014; Çavdar et al., 2018a).

Orexinergic cells of the lateral hypothalamus receive input from other areas of the hypothalamus, including the paraventricular and dorsomedial nuclei, and from the bed nucleus of the stria terminalis, central amygdala, periaqueductal gray, dorsal raphe nucleus, and lateral parabrachial nucleus (Yoshida et al., 2006; Arima et al., 2019). Other studies revealed input from the pre-Bötzinger complex (Yang and Feldman, 2018), NTS (McGovern et al., 2015b; Kawai, 2018), locus coeruleus (Jones and Moore, 1977), medial parabrachial nucleus (Saper and Loewy, 1980; Fulwiler and Saper, 1984; Moga et al., 1990; Bianchi et al., 1998), PPTg (Woolf and Butcher, 1986), cerebellar interposed nucleus (Lu et al., 2015), and cerebellar dentate nucleus (Teune et al., 2000).

### Fastigial nucleus

Electrical or chemical stimulation of the rostral part of the cerebellar fastigial nucleus can affect the respiratory pattern (Bassal and Bianchi, 1982; Lutherer and Williams, 1986; Williams et al., 1989; Xu and Frazier, 1995; Xu and Frazier, 2000; Xu et al., 2001b; Xu and Frazier, 2002; Hernandez et al., 2004). Furthermore, bilateral lesions of the fastigial nucleus suppressed spontaneous breathing in anesthetized cats (Williams et al., 1986). This effect was likely to be specific, as bilateral lesions of the cerebellar dentate nuclei had no impact of spontaneous breathing in that same study. An impact of anesthesia cannot be excluded, though, as this result could not be reproduced in awake goats (Martino et al., 2007). In contrast, the latter study describes a reduction in hypercapnia-induced increases in ventilation following bilateral lesioning of the fastigial nucleus, suggesting that the impact of the fastigial nucleus becomes especially apparent during periods with increased pCO_2_. Neural recordings demonstrated activity patterns in phase with respiration at rest, but in particular during respiratory challenges such as tracheal occlusion, bilateral carotid occlusion, or injection of sodium cyanide (Lutherer et al., 1989; Xu and Frazier, 1997; Xu and Frazier, 2002; Lu et al., 2013). Thus, the evidence converges on a role for the fastigial nucleus in mediating ventilatory responses to hypercapnia. The other two cerebellar nuclei lack the chemoreceptor abilities of the fastigial nucleus (Xu et al., 2001a; Xu and Frazier, 2002), and putative other roles of the interposed and dentate nuclei in respiratory control have not been extensively studied. However, electrical stimulation of these nuclei seemed to promote expiration (Farber, 1987; Huang et al., 1993), indicating a more widespread involvement of the cerebellum in respiratory control. The connections to and from the fastigial nucleus are described in the section on the cerebellum.

## Other sensory areas

### Paratrigeminal nucleus

The NTS and the paratrigeminal nucleus together form the main entrances of vagal sensory input, and the paratrigeminal nucleus receives mainly input from the proximal airways via the jugular ganglion (Driessen et al., 2015; McGovern et al., 2015a; McGovern et al., 2015b). The paratrigeminal nucleus also receives primary sensory input from the trigeminal, glossopharyngeal and lingual nerves, and from the upper cervical cord (Saxon and Hopkins, 2006; Panneton et al., 2017; Driessen, 2019).

The strongest projections from the paratrigeminal nucleus target the NTS and the lateral and medial parabrachial nucleus, often involving collateral projections (Menétrey et al., 1987; Saxon and Hopkins, 1998; Caous et al., 2001; de Sousa Buck et al., 2001; McGovern et al., 2015b; Driessen et al., 2018; Hashimoto et al., 2018). Other targets are the Kölliker-Fuse nucleus, ambiguus nucleus, periaqueductal gray, spinal trigeminal nucleus, and inferior olive (Caous et al., 2001; de Sousa Buck et al., 2001; McGovern et al., 2015b; Driessen et al., 2018). A projection to the cerebellum has been described (Somana and Walberg, 1979b), but this has later been questioned (Menétrey et al., 1987).

### Spinal trigeminal nucleus

The trigeminal nerve conveys sensory input from the face and terminates in the sensory trigeminal nuclei that consist of a primary and a spinal nucleus. The spinal nucleus appears particularly relevant for respiratory control, receiving not only tactile information, for example, from the mystacial vibrissae (Bosman et al., 2011), but also olfactory input from the nasal mucosa, bypassing the forebrain olfactory system (Doty et al., 1978; Anton and Peppel, 1991; Schaefer et al., 2002). Stimulation of the trigeminal olfactory system can lead to sniffs and respiratory depression without involvement of the forebrain (Pérez de Los Cobos Pallares et al., 2016). Activation of the trigeminal olfactory system occurs mainly by noxious or irritable gasses, and the resultant respiratory depression is supposed to protect the lungs, and may contribute to the diving reflex (Panneton, 2013).

The anterior ethmoidal nerve, the branchlet of the trigeminal nerve that innervates the nasal mucosa, terminates in the interpolar and caudal subnuclei of the spinal trigeminal nucleus (Anton and Peppel, 1991; Panneton et al., 2006). From these regions, projections target the pre-Bötzinger complex, Kölliker-Fuse nucleus, NTS and lateral parabrachial nucleus (Panneton et al., 2006; Zhang et al., 2018), as well as the facial nucleus (Panneton et al., 2006), lateral parafacial nucleus (Biancardi et al., 2021), cVRG (Li et al., 2021), medial parabrachial nucleus (Hashimoto et al., 2018), and periaqueductal gray (Wiberg et al., 1986; Beitz, 1989). In addition, there is direct output to the cerebellum (Van Ham and Yeo, 1992; Fu et al., 2011; Henschke and Pakan, 2020) and inferior olive (Swenson and Castro, 1983b; Huerta et al., 1985; Molinari et al., 1996; Yatim et al., 1996; Panneton et al., 2006). The latter is also indirectly targeted via the MDJ (Kubo et al., 2018).

## Premotor areas

Although direct projections from central pattern generators and respiratory sensory areas to motor neurons do exist, it is likely that indirect pathways via premotor areas are more prominent.

### Retroambiguus nucleus

The Bötzinger and pre-Bötzinger complexes form a cell column in the ventrolateral medulla that continues caudally until the first cervical spinal segment. Caudal to the pre-Bötzinger complex is the retroambiguus nucleus that houses inspiratory premotor neurons in its rostral part and expiratory premotor neurons in its caudal part (Merrill, 1970). The respiratory neurons of the retroambiguus nucleus have later been termed the ventral respiratory group. Somewhat confusingly, some authors use the terms retroambiguus nucleus and ventral respiratory group as synonyms (Shannon and Freeman, 1981), while others refer to the retroambiguus nucleus specifically as the cVRG (Subramanian and Holstege, 2009), or consider the retroambiguus nucleus and cVRG as overlapping areas within the caudal medullary reticular formation (Jones et al., 2016). For the purpose of this review, we use the terms rVRG for the inspiratory and cVRG for the expiratory part of the ventral respiratory column.

### Rostral ventral respiratory group

The rVRG provides monosynaptic input to the phrenic motor nucleus, and is the most prominent intermediate between the pre-Bötzinger complex and the diaphragmatic motor neurons (Ellenberger and Feldman, 1988; Ellenberger et al., 1990a; Tian and Duffin, 1996; Boulenguez et al., 2007; Buttry and Goshgarian, 2015). The direct pathway is complemented by a presumably weaker disynaptic pathway via premotor interneurons in the upper cervical (C1 and C2) segments (Lipski et al., 1994; Tian and Duffin, 1996; Lane et al., 2008). In addition, the rVRG projects also to the ambiguus, hypoglossal and facial motor nuclei (Yamada et al., 1988; Ellenberger et al., 1990b; Lipski et al., 1994; Zheng et al., 1998). The rVRG likely contributes to shaping the pattern of respiratory motor output, processing and transmitting sensory afferent information, coordinating ventilation with motor activity, and regulating accessory and respiratory muscle activity (Jensen et al., 2019).

The output of the rVRG is not limited to primary motor areas, but targets also the cVRG (Ellenberger and Feldman, 1990; Gerrits and Holstege, 1996) and Kölliker-Fuse nucleus (Ellenberger et al., 1990b; Lipski et al., 1994; Yokota et al., 2016), and to a lesser extent also the lateral parafacial nucleus (Biancardi et al., 2021), reticular formation (Zheng et al., 1998), retrotrapezoid nucleus (Rosin et al., 2006), NTS and lateral parabrachial nucleus (Yamada et al., 1988; Ellenberger et al., 1990a; Zheng et al., 1998), spinal trigeminal nucleus (Zheng et al., 1998), cerebellum (Gaytán and Pásaro, 1998), and inferior olive (Swenson and Castro, 1983a).

Input to the rVRG does not originate solely in the pre-Bötzinger complex, but comes also from the Bötzinger complex (Jiang and Lipski, 1990; Bryant et al., 1993; Ezure et al., 2003), Kölliker-Fuse nucleus (Ellenberger and Feldman, 1990; Zheng et al., 1998; Yokota et al., 2016), cVRG (Zheng et al., 1998), NTS (Ezure and Tanaka, 1996; Zheng et al., 1998), retrotrapezoid nucleus (Rosin et al., 2006; Bochorishvili et al., 2012; Silva et al., 2016a), and medial parabrachial nucleus (Yokota et al., 2016).

### Caudal ventral respiratory group

The cVRG is home to expiratory pre-motor neurons (Merrill, 1970; Arita et al., 1987), and therefore crucial for the control of expiration-related behavior, like vocalization and expulsive reflexes such as vomiting, coughing and sneezing (Umezaki et al., 1997; Subramanian and Holstege, 2009). While vocalization depends on the projections to the nucleus ambiguus and spinal cord, expulsive reflexes use predominantly the latter only (Holstege, 1989; Umezaki et al., 1997). In particular during coughing, the cVRG may trigger also the inspiratory phase (Cinelli et al., 2020).

Contrary to the rVRG, the cVRG does not project to the phrenic nucleus, but instead innervates motor neurons of abdominal muscles in the thoracic spinal cord, and those of the upper airway muscles in the ambiguus, hypoglossal, trigeminal, and facial nuclei (Holstege, 1989; VanderHorst et al., 2001; Ezure et al., 2003; Boers et al., 2006; Holstege and Subramanian, 2016; Jones et al., 2016). Other outputs target pre-Bötzinger and Bötzinger complexes, Kölliker-Fuse nucleus, rVRG, retrotrapezoid nucleus, lateral parabrachial nucleus and periaqueductal gray (Holstege, 1989; Gang et al., 1995; Zheng et al., 1998; Rosin et al., 2006; Jones et al., 2016). Vocalization-related input to the cVRG comes largely from the periaqueductal gray, and this connection is also associated with sexual behavior (Holstege, 1989; VanderHorst et al., 2000; Oka et al., 2008; Subramanian and Holstege, 2009; Holstege and Subramanian, 2016).

The cVRG receives both excitatory and inhibitory input from the pre-Bötzinger complex (Gerrits and Holstege, 1996; Tan et al., 2010; Yang and Feldman, 2018). Also the Bötzinger complex, Kölliker-Fuse nucleus, lateral parafacial nucleus and rVRG project to the cVRG (Fedorko and Merrill, 1984; Ellenberger and Feldman, 1990; Jiang and Lipski, 1990; Bryant et al., 1993; Gerrits and Holstege, 1996; Ezure et al., 2003; Song et al., 2012a; Silva et al., 2016a). Other direct inputs come from the NTS (Loewy and Burton, 1978; Beckstead et al., 1980; Gerrits and Holstege, 1996), retrotrapezoid nucleus (Gerrits and Holstege, 1996; Rosin et al., 2006; Bochorishvili et al., 2012; Silva et al., 2016a), and lateral and medial parabrachial nuclei (Gerrits and Holstege, 1996). A direct input from the carotid bodies to the area of the cVRG has been described (Finley and Katz, 1992), but this connection did not receive as much attention as that to the NTS.

### Reticular formation

The hindbrain reticular formation is a highly heterogenous region spanning from the pons to the caudal medulla, counting many subdivisions with often unclear borders. While many respiratory regions are connected with parts of the reticular formation, this connectivity is difficult to compare between different studies, especially considering that the nomenclature of the reticular subdivisions has been quite fluid. For this reason, we focus only on those connections with a clear role in respiratory control; other connections are summarized in Supplementary file 1. In particular, we mention three connections from the pre-Bötzinger complex in which the reticular formation serves as pre-motor area. First, there is the connection via the perihypoglossal area to the hypoglossal nucleus that is relevant for upper airway control (Chamberlin et al., 2007; Tan et al., 2010; Yang and Feldman, 2018). Second, two regions serve as premotor areas for the part of the facial nucleus that controls among others nose movements: the retrofacial area just caudal to the facial nucleus, and a part of the intermediate reticular formation, and both areas probably receive direct input from the pre-Bötzinger complex (Kurnikova et al., 2019). Third, next to the nose region of the intermediate reticular formation is the vibrissal region that links the pre-Bötzinger complex to the vibrissal part of the facial nucleus (Moore et al., 2013; Takatoh et al., 2022). The latter two pathways can contribute to the coupling of nose movements, whisking and respiration (Welker, 1964; Moore et al., 2013; Kurnikova et al., 2019; Romano et al., 2020; Takatoh et al., 2022). The reticular formation could serve a similar pre-motor function for the diaphragm as well, as in particular the gigantocellular part projects directly to the phrenic nucleus (Ellenberger et al., 1990b; Dobbins and Feldman, 1994; Lois et al., 2009).

## Limbic system

Of the limbic system, the amygdala and hypothalamus are involved in subconscious control of respiration. The former is particularly important for handling fear responses, for example, as a consequence of high levels of CO_2_, while the latter is especially important for coupling with the endocrine system, as well as for peptidergic modulation of respiration. The lateral hypothalamus is a central chemoreceptor area, and – although part of the limbic system – discussed in the section on central chemoreception.

### Central amygdala

The central amygdala plays a role in CO_2_-induced fear behavior (Ziemann et al., 2009), and electrical stimulation of the central amygdala can indeed affect breathing (Applegate et al., 1983; Harper et al., 1984; Nobis et al., 2018). In humans, amygdalar stimulation leads to hypopnea or even apnea during nasal breathing, but not during mouth breathing (Nobis et al., 2018), indicating stronger impact on the upper airways than on respiratory rhythmogenesis itself.

Renewed attention for a respiratory role of the central amygdala comes from findings in sudden unexpected death in epilepsy (SUDEP). Typically, this sequence leads to SUDEP: rapid breathing after a seizure, followed by apnea, bradycardia, and finally cardiac arrest (Ryvlin et al., 2013). Furthermore, EEG recordings in patients with epilepsy correlate amygdalar activity with seizure-induced apnea (Nobis et al., 2019).

It has been proposed that the impact of the amygdala on respiratory control is mainly exerted via its direct projection to the bed nucleus of the stria terminalis (Nobis et al., 2018). However, as the central amygdala gives rise to widespread GABAergic projections, also other connections may be relevant for respiratory control (Hopkins and Holstege, 1978; Pape and Pare, 2010; Liu et al., 2021a). Target areas include the pre-Bötzinger complex (Yang et al., 2020; Trevizan-Baú et al., 2021a), hypoglossal nucleus (Guo et al., 2020), retrotrapezoid nucleus (Rosin et al., 2006), NTS (Gasparini et al., 2020), locus coeruleus (Schwarz et al., 2015; Liu et al., 2021a), dorsal raphe (Peyron et al., 1998a; Ogawa et al., 2014; Pollak Dorocic et al., 2014; Weissbourd et al., 2014), caudal raphe (Hermann et al., 1997), lateral hypothalamus (Yoshida et al., 2006), lateral and medial parabrachial nuclei (Moga et al., 1990; Liu et al., 2021a; Yang et al., 2021), periaqueductal gray (Oka et al., 2008; Liu et al., 2021a; Trevizan-Baú et al., 2021a), and PPTg (Semba and Fibiger, 1992).

The central amygdala receives input from the NTS (Kawai, 2018), locus coeruleus (Borodovitsyna et al., 2020), dorsal raphe (Bienkowski and Rinaman, 2013), lateral and paraventricular hypothalamus (Fu et al., 2020), bed nucleus of the stria terminalis (Fu et al., 2020), lateral and medial parabrachial nuclei (Bienkowski and Rinaman, 2013), periaqueductal gray (Rizvi et al., 1991), and PPTg (Dautan et al., 2016).

### Bed nucleus of the stria terminalis

The bed nucleus of the stria terminalis is part of the extended amygdala and a main output station of the central amygdala (Liu et al., 2021a). It is central to fear, aggression and stress responses (Walker et al., 2003; Lebow and Chen, 2016). In relation to respiration, the bed nucleus of the stria terminalis may be particularly relevant for anxious responses, or even panic caused by increased levels of CO_2_ (Taugher et al., 2014). To this end, the bed nucleus of the stria terminalis expresses relatively high levels of acid-sensing ion channel 1A (ASIC1A) (Coryell et al., 2007; Taugher et al., 2014). ASIC1A in the bed nucleus of the stria terminalis is also essential for the freezing reaction of mice, which implicates a strong reduction in breathing, upon exposure to a predator odor (Taugher et al., 2015).

Apart from the central amygdala (Dong et al., 2001a; Shin et al., 2008; Lebow and Chen, 2016; Ni et al., 2016), other areas targeting the bed nucleus of the stria terminalis are the lateral and dorsomedial hypothalamus (Shin et al., 2008), NTS (Ricardo and Tongju Koh, 1978; Shin et al., 2008; Bienkowski and Rinaman, 2013; Ni et al., 2016; Kawai, 2018), locus coeruleus (Ni et al., 2016), dorsal raphe nucleus (Vertes, 1991; Shin et al., 2008; Bienkowski and Rinaman, 2013; Ni et al., 2016), lateral parabrachial nucleus (Shin et al., 2008; Bienkowski and Rinaman, 2013; Ni et al., 2016; Jaramillo et al., 2021), and periaqueductal gray (Shin et al., 2008; Ni et al., 2016). Relating to their functional similarities, the central amygdala and bed nucleus of the stria terminalis receive input from the same brain regions, often even from collaterals, except from the substantia nigra pars compacta, that does not project to the bed nucleus of the stria terminalis (Bienkowski and Rinaman, 2013). This does not imply that all projections are equally strong. In particular, the parabrachial nuclei project stronger to the central amygdala, and the NTS stronger to the bed nucleus of the stria terminalis (Bienkowski and Rinaman, 2013).

Output reaches the central amygdala (Dong et al., 2001b; Fu et al., 2020), lateral, dorsomedial and paraventricular hypothalamus (Yoshida et al., 2006; Barbier et al., 2021), NTS (Gasparini et al., 2020), locus coeruleus (Luppi et al., 1995; Schwarz et al., 2015), dorsal raphe nuclei (Peyron et al., 1998a; Lee et al., 2003; Ogawa et al., 2014; Pollak Dorocic et al., 2014; Weissbourd et al., 2014), caudal raphe (Hermann et al., 1997), lateral and medial parabrachial nuclei (Moga et al., 1990; Luskin et al., 2021), and PPTg (Steininger et al., 1992).

### Paraventricular hypothalamus

The paraventricular nucleus of the hypothalamus is vital for the hypoxia reflex: decreased heart rate and increased blood pressure and phrenic nerve activity (Kubo et al., 1997; Reddy et al., 2005; Ruyle et al., 2019). The paraventricular nucleus is reciprocally connected with the NTS (Rinaman, 1998; Geerling et al., 2010; King et al., 2012; Gasparini et al., 2020), and the NTS serves both as intermediate for the ascending input coming from the carotid bodies, and for the descending output affecting the phrenic nucleus (Ruyle et al., 2018; Ruyle et al., 2019). The impact of these NTS-projecting paraventricular fibers may be complemented by vasopressinergic connections to the phrenic nucleus (Kc et al., 2002), oxytocinergic projections to the pre-Bötzinger complex (Mack et al., 2002; Yang et al., 2020; Trevizan-Baú et al., 2021a), or by projections to the Kölliker-Fuse nucleus (Yokota et al., 2016; Trevizan-Baú et al., 2021a), PiCo (Oliveira et al., 2021), ambiguus, hypoglossal and facial motor nuclei (Mack et al., 2007; Geerling et al., 2010; Guo et al., 2020), reticular formation (Geerling et al., 2010), retrotrapezoid nucleus (Rosin et al., 2006; Geerling et al., 2010), locus coeruleus (Luppi et al., 1995; Zheng et al., 1995; Schwarz et al., 2015), dorsal and caudal raphe (Zheng et al., 1995; Hermann et al., 1997; Peyron et al., 1998a; Lee et al., 2003; Geerling et al., 2010; Ogawa et al., 2014; Pollak Dorocic et al., 2014; Weissbourd et al., 2014; Singh et al., 2022), lateral hypothalamus (Yoshida et al., 2006; Singh et al., 2022), lateral and medial parabrachial nuclei (Zheng et al., 1995; Geerling et al., 2010; Singh et al., 2022), periaqueductal gray (Zheng et al., 1995; Geerling et al., 2010; Trevizan-Baú et al., 2021a; Singh et al., 2022), bed nucleus of the stria terminalis (Singh et al., 2022), central amygdala (Fu et al., 2020), spinal trigeminal and paratrigeminal nuclei (Zheng et al., 1995; Geerling et al., 2010), PPTg (Zheng et al., 1995; Geerling et al., 2010), and cerebellum (Dietrichs, 1984; Çavdar et al., 2018a).

Apart from the NTS, the lateral and dorsomedial nucleus of the hypothalamus (Thompson et al., 1996; Singh et al., 2022), locus coeruleus (Jones and Moore, 1977; Jones and Yang, 1985; Cunningham and Sawchenko, 1988), bed nucleus of the stria terminalis (Barbier et al., 2021), and lateral parabrachial nucleus (Saper and Loewy, 1980; Fulwiler and Saper, 1984; Moga et al., 1990) project to the paraventricular nucleus.

### Dorsomedial hypothalamus

The dorsomedial hypothalamus is essential for mediating respiratory effects of stressful stimuli (Bondarenko et al., 2015). To this end, it receives input from other hypothalamic regions and the bed nucleus of the stria terminalis, as well as from the periaqueductal gray, lateral parabrachial nucleus, NTS (Thompson and Swanson, 1998; Do et al., 2020), pre-Bötzinger complex (Yang and Feldman, 2018), and Kölliker-Fuse nucleus (Geerling et al., 2017).

The dorsomedial hypothalamus projects predominantly to other hypothalamic nuclei, in particular to the paraventricular nucleus, but also to the bed nucleus of the stria terminalis, periaqueductal gray, NTS (Thompson et al., 1996), Kölliker-Fuse nucleus (Trevizan-Baú et al., 2021a), dorsal and caudal raphe (Peyron et al., 2018; Trevizan-Baú et al., 2021a), and cerebellar cortex (Çavdar et al., 2018a).

## Modulatory brain regions

On top of the central pattern generators, (pre)motor areas, sensory areas and limbic system, also other brain regions can affect specific aspects of respiration, often to adapt respiration to other behaviors. In fact, few brain regions are completely unrelated to respiration. In the following section, we restrict ourselves to those brain regions of which a non-voluntary respiratory function has been clearly described.

### Lateral parabrachial nucleus

The parabrachial nuclei produce a tonic excitatory drive that can, in conjunction with output from the Kölliker-Fuse nucleus, contribute to setting the duration of inspiration and expiration (Navarrete-Opazo et al., 2020). In addition, the lateral parabrachial nucleus is also crucial in the hypercapnic arousal reflex that can trigger waking up during sleep (Kaur et al., 2013). It has been suggested that frequent hypercapnic arousals contribute to day-time fatigue and cardiovascular problems in patients with obstructive sleep apnea (Abbott and Souza, 2021). The lateral parabrachial nucleus has a tonic descending drive and an event-driven ascending one. The latter is consistent with other functions of the lateral parabrachial nucleus, as it responds to various aversive signals, varying from food poisoning to itch and hypercapnia, and can send a general alarm signal to the forebrain (Palmiter, 2018; Chiang et al., 2019; Jaramillo et al., 2021).

The main outputs target the lateral hypothalamus (Saper and Loewy, 1980; Fulwiler and Saper, 1984; Moga et al., 1990; Bianchi et al., 1998; Yoshida et al., 2006; Arima et al., 2019), bed nucleus of the stria terminalis (Saper and Loewy, 1980; Fulwiler and Saper, 1984; Moga et al., 1990; Bianchi et al., 1998; Shin et al., 2008; Bienkowski and Rinaman, 2013; Ni et al., 2016), central amygdala (Saper and Loewy, 1980; Moga et al., 1990; Bianchi et al., 1998; Tokita et al., 2010; Bienkowski and Rinaman, 2013), and thalamus (Jaramillo et al., 2021). Other targets are the pre-Bötzinger and Bötzinger complexes (Gang et al., 1995; Yang et al., 2020; Yu et al., 2022), lateral parafacial nucleus (Biancardi et al., 2021), cVRG (Gerrits and Holstege, 1996), ambiguus nucleus (Rübsamen and Schweizer, 1986), hypoglossal nucleus (Yokota et al., 2015), retrotrapezoid nucleus (Rosin et al., 2006), NTS (Saper and Loewy, 1980; Herbert et al., 1990; Bianchi et al., 1998), locus coeruleus (Luppi et al., 1995), dorsal and caudal raphe (Saper and Loewy, 1980; Hermann et al., 1997; Bianchi et al., 1998; Lee et al., 2003; Peyron et al., 2018), paraventricular nucleus (Saper and Loewy, 1980; Fulwiler and Saper, 1984; Moga et al., 1990; Singh et al., 2022), periaqueductal gray (Bianchi et al., 1998), and PPTg (Steininger et al., 1992).

The main ascending inputs to the lateral parabrachial nucleus come from the spinal cord and NTS (Palmiter, 2018). Other inputs come from the pre-Bötzinger and Bötzinger complexes (Tan et al., 2010; Yang and Feldman, 2018), Kölliker-Fuse nucleus (Song et al., 2012a; Geerling et al., 2017), rVRG (Holstege, 1989), NTS (Beckstead et al., 1980; Herbert et al., 1990; McGovern et al., 2015b; Kawai, 2018; Yu et al., 2022), retrotrapezoid nucleus (Rosin et al., 2006; Bochorishvili et al., 2012; Silva et al., 2016a), locus coeruleus (Robertson et al., 2013; Yang et al., 2021), dorsal raphe (Petrov et al., 1992; Quattrochi et al., 1998; Kaur et al., 2020), bed nucleus of stria terminalis (Moga et al., 1990), central amygdala (Moga et al., 1990; Yang et al., 2021), paraventricular nucleus (Zheng et al., 1995; Geerling et al., 2010; Singh et al., 2022), spinal trigeminal nucleus (Panneton et al., 2006; Zhang et al., 2018), paratrigeminal nucleus (Saxon and Hopkins, 1998; Caous et al., 2001; McGovern et al., 2015b; Driessen et al., 2018), PPTg (Quattrochi et al., 1998; Lima et al., 2019b), and fastigial nucleus (Teune et al., 2000).

### Medial parabrachial nucleus

Activity of the medial parabrachial nucleus affects, like that of the lateral parabrachial nucleus, the breathing frequency by modulating the duration of expiration (von Euler et al., 1976; Zuperku et al., 2017). The medial parabrachial nucleus contains more expiratory neurons than the neighboring Kölliker-Fuse nucleus that has more inspiratory and phase-spanning neurons (Song et al., 2006). The medial parabrachial nucleus has a dense expression of µ-opioid receptors (Ding et al., 1996) and plays an important role in the mediation of respiratory depression by opioids (Miller et al., 2017; Algera et al., 2019; Saunders and Levitt, 2020). Unlike the lateral parabrachial nucleus, the medial parabrachial nucleus is not involved in the hypercapnic arousal reflex (Kaur et al., 2013).

The medial parabrachial nucleus has both excitatory and inhibitory projections to pre-inspiratory neurons in the pre-Bötzinger complex and to expiratory neurons in the Bötzinger complex (Zuperku et al., 2019). Activity of the medial parabrachial nucleus can be affected by input from pulmonary stretch receptors coming via the NTS (Zuperku et al., 2021), from which it receives direct input (Herbert et al., 1990). In addition, it receives input from chemosensitive neurons of the caudal raphe nucleus (Brust et al., 2014).

The medial parabrachial nucleus receives also input from the pre-Bötzinger complex (Tan et al., 2010; Yang and Feldman, 2018), Kölliker-Fuse nucleus (Song et al., 2012a; Geerling et al., 2017), rVRG (Holstege, 1989), NTS (Loewy and Burton, 1978; Herbert et al., 1990; McGovern et al., 2015b), retrotrapezoid nucleus (Rosin et al., 2006), locus coeruleus (Robertson et al., 2013), caudal raphe (Brust et al., 2014), bed nucleus of stria terminalis (Moga et al., 1990; Luskin et al., 2021), central amygdala (Moga et al., 1990; Liu et al., 2021a; Yang et al., 2021), paraventricular nucleus (Zheng et al., 1995; Geerling et al., 2010; Singh et al., 2022), spinal trigeminal nucleus (Hashimoto et al., 2018), paratrigeminal nucleus (Saxon and Hopkins, 1998; Caous et al., 2001; McGovern et al., 2015b; Driessen et al., 2018), Purkinje cells in the cerebellar cortex (Sugihara et al., 2009; Hashimoto et al., 2018), and fastigial nucleus (Teune et al., 2000).

The medial parabrachial nucleus projects to the pre-Bötzinger and Bötzinger complexes (Gang et al., 1995; Yang et al., 2020), lateral parafacial nucleus (Biancardi et al., 2021), rVRG and cVRG (Gerrits and Holstege, 1996; Yokota et al., 2016), ambiguus and hypoglossal nuclei (Saper and Loewy, 1980; Rübsamen and Schweizer, 1986; Núñez-Abades et al., 1990; Guo et al., 2020), retrotrapezoid nucleus (Rosin et al., 2006), NTS (Herbert et al., 1990; Bianchi et al., 1998), locus coeruleus (Luppi et al., 1995), dorsal and caudal raphe (Saper and Loewy, 1980; Hermann et al., 1997; Bianchi et al., 1998; Lee et al., 2003; Peyron et al., 2018), lateral hypothalamus (Saper and Loewy, 1980; Fulwiler and Saper, 1984; Moga et al., 1990; Bianchi et al., 1998), bed nucleus of the stria terminalis (Bianchi et al., 1998; Bienkowski and Rinaman, 2013; Ni et al., 2016), central amygdala (Fulwiler and Saper, 1984; Moga et al., 1990; Bienkowski and Rinaman, 2013), periaqueductal gray (Bianchi et al., 1998), and cerebellum (Fu et al., 2011).

### Periaqueductal gray

The periaqueductal gray, also known as the central gray, contributes to maintaining the homeostatic balance of an individual. To this end it participates in controlling defensive, emotional, social, sexual and autonomic behaviors, but also in modulating unpleasant sensations, such as pain, itch or the urge to cough (Bandler et al., 2000; Linnman et al., 2012; Motta et al., 2017). In addition, the periaqueductal gray is central to the control of vocalizations (Magoun et al., 1937; Zhang et al., 1994; Esposito et al., 1999; Jürgens, 2002; Subramanian et al., 2021) via its strong projection to the cVRG that is the only brain area directly targeting all motor areas required for vocalization (VanderHorst et al., 2000; Holstege and Subramanian, 2016). This pathway is especially relevant for emotional vocalizations, like crying and laughing, that do not rely on Broca’s area, as human speech does (Holstege and Subramanian, 2016). For this reason, the periaqueductal gray can be seen as the laughing center, receiving excitatory input from emotion-related pathways originating from the basal temporal and frontal lobes, limbic system, and basal ganglia, and inhibition from voluntary systems, in particular (pre)motor cortices (Wild et al., 2003; Klingbeil et al., 2021). As to the suppression of involuntary laughter, the periaqueductal gray can also contribute to the suppression of the urge to cough. The periaqueductal gray sends predominantly GABAergic fibers to the NTS (Chen et al., 2022; Figure 5F). This can have clinical relevance, as the common condition of chronic cough is thought to be caused by hypersensitivity to airway stimulation (Morice et al., 2020).

Although the periaqueductal gray lacks clearly discernible cell groups, specific functions can be roughly attributed to four longitudinal columns that differ in their connectivity patterns (Carrive, 1993). The lateral (lPAG) and dorsolateral (dlPAG) columns are mostly involved in active coping strategies, such as fight or flight, while the ventrolateral (vlPAG) column is associated with passive coping strategies, like freezing (Bandler et al., 2000; Linnman et al., 2012; Faull et al., 2019).

The periaqueductal gray subserves several respiratory functions to support stereotypical behavioral responses. These functions are distributed over the four cell columns, largely in line with their general function (Subramanian et al., 2008; Subramanian, 2013; Faull et al., 2019). Chemical stimulation of the dlPAG triggers active breathing, as occurring during fight and flight situations, while stimulation of the dorsomedial and the medial part of the lPAG resulted in slow deep breathing and dyspnea, and inspiratory apneusis, respectively. Finally, stimulation of the lateral parts of lPAG and vlPAG induced respiratory patterns associated with vocalizations (Subramanian et al., 2008).

The periaqueductal gray is an important hub between the fore- and hindbrain, receiving input from multiple cortical areas as well as from the lateral, dorsomedial and paraventricular hypothalamus (Beitz, 1989; Zheng et al., 1995; Thompson et al., 1996; Geerling et al., 2010; Trevizan-Baú et al., 2021a; Singh et al., 2022). Ascending input to the periaqueductal gray comes directly from the spinal cord, and in particular from the upper cervical cord, to the lPAG and vlPAG (Keay et al., 1997). Also the NTS, the prime recipient of vagal sensory input, projects to the ventrolateral and medial PAG (Herbert and Saper, 1992; Kawai, 2018). Other respiratory centers in the brainstem also project to the lPAG and vlPAG: the pre-Bötzinger and Bötzinger complexes, Kölliker-Fuse nucleus (Tan et al., 2010; Yang and Feldman, 2018; Trevizan-Baú et al., 2021b), locus coeruleus (Jones and Moore, 1977; Jones and Yang, 1985), dorsal and caudal raphe (Vertes, 1991; Herbert and Saper, 1992; Trevizan-Baú et al., 2021b), lateral and medial parabrachial nuclei (Bianchi et al., 1998), paratrigeminal nucleus (McGovern et al., 2015b), and fastigial and dentate cerebellar nuclei (Teune et al., 2000; Frontera et al., 2020).

Descending output from the periaqueductal gray targets multiple respiratory centers. All four columns, but in particular the lPAG and vlPAG, target the pre-Bötzinger complex, Kölliker-Fuse nucleus and lateral parafacial nucleus (Tan et al., 2010; Yang and Feldman, 2018; Biancardi et al., 2021; Trevizan-Baú et al., 2021b). Ipsilateral connections to the parafacial nucleus are predominantly glutamatergic, while contralateral projections have a mixture of glutamatergic and GABAergic fibers (Biancardi et al., 2021). The periaqueductal gray innervates also the PiCo (Oliveira et al., 2021), retrotrapezoid nucleus (Rosin et al., 2006), NTS (VanderHorst et al., 2000; Chen et al., 2022), locus coeruleus (Luppi et al., 1995; Schwarz et al., 2015), dorsal and caudal raphe (Hermann et al., 1997; Ogawa et al., 2014; Pollak Dorocic et al., 2014; Peyron et al., 2018; Trevizan-Baú et al., 2021b), PPTg (Semba and Fibiger, 1992; Steininger et al., 1992), and inferior olive (Swenson and Castro, 1983b; VanderHorst et al., 2000). Forebrain projections include the lateral hypothalamus (Woolf and Butcher, 1986; Yoshida et al., 2006), bed nucleus of the stria terminalis (Shin et al., 2008; Ni et al., 2016), and central amygdala (Rizvi et al., 1991).

### Tegmentum

The pedunculopontine tegmental nucleus (PPTg) and the adjacent laterodorsal tegmental nucleus provide important sources of cholinergic fibers, the activation of which is sufficient to induce REM sleep during non-REM sleep (Van Dort et al., 2015). To this end, the pedunculopontine and laterodorsal tegmental nuclei provide widespread innervation of the thalamus and basal ganglia (Mena-Segovia and Bolam, 2017), but also of the retrotrapezoid nucleus (Lima et al., 2019b; Silva et al., 2019). As the chemoreceptor activity in the retrotrapezoid nucleus can be modulated by acetylcholine (Sobrinho et al., 2016), this could imply a different sensitivity to pCO_2_ during REM sleep. Indeed, partial lesioning of the PPTg resulted in abnormal sleep patterns during intermittent hypoxia, while leaving basic respiratory parameters intact (Fink et al., 2021).

Pharmacological activation of muscarinic acetylcholine receptors in the PPTg resulted in decreased breathing rates in awake rats (Lima et al., 2019a), and also glutamate injections could suppress breathing in anesthetized rats (Saponjic et al., 2003; Topchiy et al., 2010). Inhibition of the PPTg resulted in increased inspiratory activity (Silva et al., 2019). Overall, it seems that the PPTg can inhibit the function of the retrotrapezoid nucleus, and thereby modulate the respiratory drive. This may be particularly relevant during REM sleep, given the interaction between PPTg, hypoxia and disrupted sleep patterns (Silva et al., 2019; Fink et al., 2021).

The target areas of the PPTg are not restricted to those mentioned above, but also include the Kölliker-Fuse nucleus (Lima et al., 2019b), lateral parafacial nucleus (Biancardi et al., 2021), hypoglossal, trigeminal and facial motor nuclei (Woolf and Butcher, 1989), locus coeruleus (Woolf and Butcher, 1989), dorsal and caudal raphe (Woolf and Butcher, 1989; Hermann et al., 1997; Ogawa et al., 2014; Lima et al., 2019b), lateral hypothalamus (Woolf and Butcher, 1986), bed nucleus of the stria terminalis (Ni et al., 2016), central amygdala (Dautan et al., 2016), lateral parabrachial nucleus (Quattrochi et al., 1998; Lima et al., 2019b), spinal trigeminal nucleus (Woolf and Butcher, 1989), and all three cerebellar nuclei (Woolf and Butcher, 1989; Ruggiero et al., 1997; Vitale et al., 2016).

Input to the PPTg comes from the NTS (Steininger et al., 1992), locus coeruleus (Steininger et al., 1992), dorsal and caudal raphe (Vertes, 1991; Semba and Fibiger, 1992; Steininger et al., 1992), lateral and paraventricular hypothalamus (Semba and Fibiger, 1992; Steininger et al., 1992; Zheng et al., 1998; Geerling et al., 2010), bed nucleus of the stria terminalis (Steininger et al., 1992), central amygdala (Semba and Fibiger, 1992), lateral parabrachial nucleus (Steininger et al., 1992), and cerebellar nuclei (Hazrati and Parent, 1992; Steininger et al., 1992).

## Basal ganglia

Despite their widespread connectivity and importance for motor control (Roseberry et al., 2016; Lee et al., 2020; Quartarone et al., 2020), the basal ganglia are not typically considered to be part of the respiratory control network. Possibly the best-known consequence of basal gangliar dysfunction is Parkinson’s disease, caused by progressive degeneration of dopaminergic neurons in the substantia nigra (Kalia and Lang, 2015). Patients with Parkinson’s disease can be confronted with respiratory problems, but these may be attributed to degeneration of respiratory muscles, and therefore not directly to respiratory control (Guedes et al., 2012; Baille et al., 2018; Guilherme et al., 2021). Patients with Parkinson’s disease may also experience sleep-disturbed breathing or dyspnea (Docu Axelerad et al., 2021). With a prevalence of 12%, dyspnea is a relatively rare symptom of Parkinson’s disease (Barone et al., 2009), and its cause is unclear (Docu Axelerad et al., 2021). Overall, the basal ganglia, and the substantia nigra in particular, therefore, do not seem to play a major role in respiratory control.

Nevertheless, there are anatomical connections between the substantia nigra and the respiratory control centers, including a direct pathway to the pre-Bötzinger complex (Yang et al., 2020). In a rat model for Parkinson’s disease, modulation of the retrotrapezoid nucleus by the substantia nigra indirectly, via the periaqueductal gray, could contribute to respiratory control (Lima et al., 2018). Given the uncertainty of the impact of the basal ganglia on respiratory control, these outputs of the substantia nigra have been hypothesized to provide respiratory control centers with information on other ongoing movements, to facilitate respiratory control into the behavioral context of the animal (Yang et al., 2020). For this reason, we did not further consider the basal ganglia as control center for the subconscious regulation of respiration.

## Cerebral cortex

**Figure 7.**
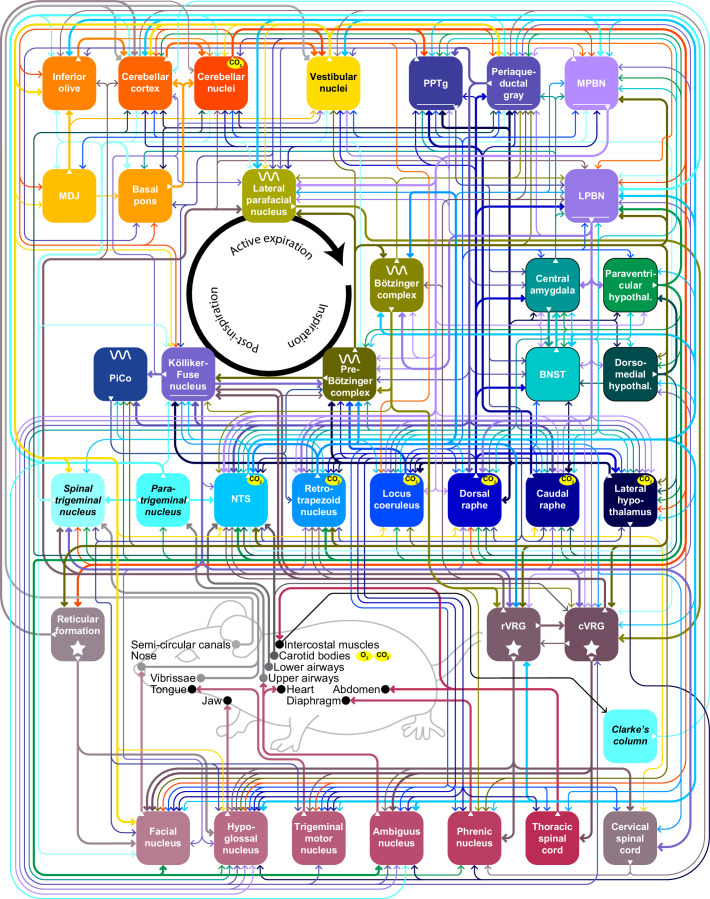
Main pathways underlying subconscious control of respiration Schematic drawing representing the brain areas most involved in subconscious control of respiration and the main pathways connecting them. Fat lines indicate relatively strong connections and thin lines moderately strong ones. Sparse connections are not included in this scheme. When an area is marked with ‘CO_2_’ it contains central chemoreceptor properties, while the carotid bodies sense blood levels of CO_2_ and of O_2_. The central pattern generators are indicated with a sine wave. BNST = bed nucleus of the stria terminalis; cVRG = caudal ventral respiratory group; LPBN = lateral parabrachial nucleus; MPBN = medial parabrachial nucleus; NTS = nucleus tractus solitarii; PiCo = postinspiratory complex; PPTg = peripeduncular tegmental nucleus; rVRG = rostral ventral respiratory group. See also Supplementary file 1.

Breathing is rather unique among the autonomic functions as it can be subjected to volitional control, modulated by the emotional state, adapted to other ongoing behaviors, and vital for vocalizations and human speech. It is quite likely that the cerebral cortex plays an important role in these functions (Herrero et al., 2018). Unlike lesions of the subcortical, and in particular of the medullar respiratory centers, lesions of cortical respiratory areas do not abolish respiration (Ramirez et al., 2011). The anatomical aspects of cortical modulation of respiration have received relatively little attention, and we consider the integration of the thalamocortical system into the anatomical scheme of Figure 7 an important future task.

There is early evidence for a direct projection from the cat motor cortex to the phrenic nucleus, bypassing the medullary respiratory circuitry (Rikard-Bell et al., 1985). In addition, a wide spectrum of projections from different cortical areas targets subcortical respiratory control areas, with the most numerous projections targeting the periaqueductal gray and the Kölliker-Fuse nucleus, and less abundant projections to the caudal raphe nucleus, Bötzinger and pre-Bötzinger complexes (Trevizan-Baú et al., 2021a). In this study, the Kölliker-Fuse nucleus was predominantly targeted from the (facial) somatosensory, endopiriform and rhinal cortices, and the periaqueductal gray from the insular, cingulate, motor, pre- and infralimbic cortices. In addition, the NTS receives a direct projection form the insular cortex (Torrealba and Müller, 1996; Gasparini et al., 2020).

In the context of the present review, especially the function of the insular cortex is relevant. It appears to be the prime cortical target area of visceral input, and it has a viscerotopic organization (Cechetto and Saper, 1987; Bagaev and Aleksandrov, 2006; Livneh and Andermann, 2021). EEG recordings of the insular cortex revealed coherence with breathing (Herrero et al., 2018). Depending on the location within the insular cortex, microstimulation could either increase or decrease the respiratory rate (Bagaev and Aleksandrov, 2006), suggesting that the insular cortex can affect respiration in response to vagal sensory input.

## Cerebellum

The cerebellum is composed of a cortex surrounding the fastigial, interposed and dentate nuclei. The output of the cerebellar cortex is formed by Purkinje cells that predominantly target the cerebellar nuclei (Voogd and Glickstein, 1998), although direct connections to the vestibular nuclei, locus coeruleus, and lateral and medial parabrachial nuclei exist as well (Zeeuw and Berrebi, 1995; Sadakane et al., 2000; Sugihara et al., 2009; Schwarz et al., 2015; Hashimoto et al., 2018; Novello et al., 2022). From the cerebellar nuclei, there are projections back to the cerebellar cortex (Tolbert et al., 1977; Gao et al., 2016), inhibitory projections to the inferior olive (Voogd and Glickstein, 1998), and excitatory projections to the MDJ (De Zeeuw and Ruigrok, 1994; Wang et al., 2022) and many brain regions relevant for respiratory control.

Strikingly, there are no direct connections to the phrenic nucleus (Lois et al., 2009), nor to the pre-Bötzinger complex (Yang et al., 2020). Instead, diaphragmatic activity can be affected by a direct projection to the premotor rVRG (Gaytán and Pásaro, 1998). Although there are direct projections from the cerebellum to the trigeminal motor nucleus, affecting upper airway muscles (Judd et al., 2021), facial nucleus (Moolenaar and Rucker, 1976; Fujita et al., 2020), and hypoglossal nucleus (Guo et al., 2020; Judd et al., 2021), these direct projections to motor nuclei are typically relatively sparse so that indirect projections, for example, via the reticular formation, are probably much more abundant (Novello et al., 2022).

Other cerebellar projections that can be relevant for respiratory control target the Kölliker-Fuse nucleus (Fujita et al., 2020), locus coeruleus (Teune et al., 2000; Schwarz et al., 2015), dorsal raphe nuclei (Teune et al., 2000; Çavdar et al., 2018b), periaqueductal gray (Teune et al., 2000; Frontera et al., 2020; Fujita et al., 2020; Judd et al., 2021), lateral and paraventricular hypothalamus (Dietrichs et al., 1994; Zhu et al., 2006). Many of these areas, in particular the Kölliker-Fuse nucleus, have profound projections to medullary respiratory circuit, so that these projections can explain how the cerebellum can affect respiratory control.

Despite the absence of strong pathways from the cerebellum to the respiratory pattern generators and motor nuclei, cerebellar activity can profoundly modulate respiration. Functional brain imaging of healthy human subjects revealed that the cerebellum is particularly active during coping with respiratory challenges: broad activation during hypoxia and slow breathing (Critchley et al., 2015), and specific activation in lobules V and VI during hypercapnia (Colebatch et al., 1991; Kastrup et al., 1999). Also the cerebellar nuclei are active during hypercapnia and hypoxia (McKay et al., 2010), as well as during volitional expiration (Ramsay et al., 1993). Changes in environmental air pressure can trigger cerebellar activity as well (Isaev et al., 2002; Raux et al., 2013; Figure 6G–J).

The notion that the cerebellum is particularly relevant for deviating from rhythmic breathing is corroborated by the finding that mice suffering from a complete lack of output from the cerebellar cortex or from a partial loss of cerebellar output neurons display overly regular breathing at rest (Liu et al., 2020; Taylor et al., 2022; Figure 6F). Stimulation of the fastigial nucleus in cats can affect phrenic nerve activity, and in particular terminate expiratory activity when stimulated during expiration (Zhang et al., 1999), analogous to optogenetic stimulation experiments of Purkinje cells (Romano et al., 2020; Figure 6E). And indeed, Lurcher mice that suffer from a complete loss of Purkinje cells during their development, demonstrated impaired responses to hypercapnic and hypoxic challenges (Calton et al., 2014; Calton et al., 2016). Neuronal activity of the cerebellar cortex and the fastigial nucleus is linked to respiration (Lutherer et al., 1989; Gruart and Delgado-García, 1992; Cao et al., 2012b; Lu et al., 2013; Romano et al., 2020; Figure 6C–D). At rest, modulation of the activity of Purkinje cell occurs without phase lead or lag relative to the respiratory cycle (Romano et al., 2020), and one could speculate that the cerebellum does not only act to adapt respiration to ongoing behavior, but also facilitates adaptation of other behavior to respiration, in line with observations in cerebellar patients (Ebert et al., 1995).

The cerebellum may, in addition, have a particular role in the regulation of breathing during sleep, which includes different stages with different respiratory and oculomotor dynamics (Canto et al., 2017; De Zeeuw and Canto, 2022; Pujol et al., 2022). Indeed, when shifting from awake to subconscious behavior and vice-versa, different brain structures such as cerebellum, hippocampus and amygdala, are dynamically activated and de-activated in line with changes in breathing (Pujol et al., 2022). Accordingly, the impact of sleep on breathing and its role in the development of diurnal respiratory failure in patients suffering from hypoventilation and/or cerebellar disorders can be overlooked (Piper and Yee, 2014; Canto et al., 2017). It remains to be elucidated which sets of mechanisms are involved in this process, but it should be noted that sleep by itself does not only reduce respiratory drive, but also diminishes responsiveness to hypoxia and hypercapnia. For example, acute increases in CO_2_ during rapid eye movement sleep can initiate the process of bicarbonate retention, which further depresses ventilatory responsiveness (Piper and Yee, 2014) and which in turn can affect activity in the cerebellar nuclei (Parsons et al., 2001; Martino et al., 2007).

### Mossy fiber inputs

Mossy fibers constitute one of the two main glutamatergic input pathways to the cerebellum. They carry ascending as well as descending information to granule cells in the cerebellar cortex, while often forming collaterals to the cerebellar nuclei (van der Want et al., 1987; Huang et al., 2013; Ruigrok et al., 2015). Cerebellar granule cells make up about halve of all neurons in the brain, and their bifurcating axons, the parallel fibers, target Purkinje cells directly, and indirectly via inhibitory interneurons (Harvey and Napper, 1991; De Zeeuw et al., 2011; Ruigrok et al., 2015).

The pontine nuclei, consisting of the basal pons and the nucleus reticularis tegmenti pontis, are the prime source of mossy fibers; not only are they the main intermediate for descending pathways from the cerebral cortex, they also receive subcortical input from among others the lateral hypothalamus, central amygdala, periaqueductal gray, spinal trigeminal nucleus, PPTg, MDJ, and cerebellar nuclei (Mihailoff et al., 1989; Brodal and Bjaalie, 1992; Liu and Mihailoff, 1999; Pijpers and Ruigrok, 2006; Bosman et al., 2011; Fu et al., 2011; Huang et al., 2013; Ruigrok et al., 2015; Henschke and Pakan, 2020).

Other important mossy fiber sources are the two vagal sensory nuclei relating respiratory visceral input to the brain, the NTS (Batini et al., 1978; Somana and Walberg, 1979a; Saigal et al., 1980a; Fu et al., 2011) and paratrigeminal nucleus (Somana and Walberg, 1979b). Strong mossy fiber connections bind also the spinal trigeminal nucleus, medial parabrachial nucleus, and several parts of the medullary reticular formation with the cerebellum (Ruigrok et al., 1995; Yatim et al., 1996; Fu et al., 2011). This is also true for multiple spinal regions (Sengul et al., 2015; Baek et al., 2019). Weaker projections come from the lateral parabrachial nucleus (Fu et al., 2011), Kölliker-Fuse nucleus (Fu et al., 2011), and rVRG (Gaytán and Pásaro, 1998). The pre-Bötzinger complex provides only sparse projections to the cerebellum (Yang and Feldman, 2018).

### Climbing fiber inputs

The other main input to the cerebellum is formed by the climbing fibers that originate exclusively from the contralateral inferior olive and that form extraordinarily strong glutamatergic synapses on Purkinje cells, next to relatively weak collateral projections to the cerebellar nuclei (Szentágothai and Rajkovits, 1959; Ruigrok et al., 2015; Lu et al., 2016). Where each adult Purkinje cell can receive input from over 100,000 parallel fibers, it is typically innervated by only a single climbing fiber (Harvey and Napper, 1991; Bosman and Konnerth, 2009). Climbing fiber spikes invariably trigger complex spike firing by the postsynaptic Purkinje cells, and these consist of normal action potentials in conjunction with dendritic spikelets caused by profound influx of calcium (Llinás and Sugimori, 1980; De Zeeuw et al., 2011). Because of their impact on intracellular calcium levels, complex spikes affect the synaptic strength of parallel fiber inputs, and thereby control cerebellar learning (Ito, 2000; Coesmans et al., 2004; Ohtsuki et al., 2009; van Woerden et al., 2009; Gao et al., 2012; Yang and Lisberger, 2014; Romano et al., 2018). Accordingly, climbing fiber activity is strictly regulated, occurs at a sustained, but low rate, and can thereby serve a homeostatic function at rest, while reporting salient events when they occur (Zhou et al., 2014; Ju et al., 2019; Negrello et al., 2019; Bina et al., 2021). In general, the complex spikes therefore modulate the activity pattern of simple spikes, that organize motor output.

Apart from the cerebellum, the most important input areas to the inferior olive are the spinal cord (Brown et al., 1977), periaqueductal gray (Brown et al., 1977; Swenson and Castro, 1983b; VanderHorst et al., 2000), spinal trigeminal nucleus (Swenson and Castro, 1983b; Huerta et al., 1985; Van Ham and Yeo, 1992; Yatim et al., 1996), and MDJ (de Zeeuw et al., 1989; Wang et al., 2022). The MDJ is the main intermediate between the cerebral cortex and the inferior olive: it relays input from the rostromedial and caudal parts of the cerebral cortex to the principal olive, and from the rostrolateral parts of the cerebral cortex to the medial accessory olive (Wang et al., 2022). In addition, the MDJ receives input from the spinal trigeminal nucleus (Kubo et al., 2018) and the cerebellar nuclei (De Zeeuw and Ruigrok, 1994; Wang et al., 2022). Input to the inferior olive comes also from the two vagal sensory recipient nuclei: NTS (Loewy and Burton, 1978; McGovern et al., 2015b) and paratrigeminal nucleus (McGovern et al., 2015b), as well as from rVRG (Swenson and Castro, 1983b).

### Other cerebellar inputs

Other cerebellar afferents could potentially contribute to respiratory control, although their precise impact has not yet been studied. Hypothalamocerebellar connections are evolutionary preserved, but more prominent in primates than in rodents (Dietrichs, 1984; Dietrichs et al., 1994). They originate from several hypothalamic nuclei. In the context of this review, orexinergic fibers from the lateral hypothalamus to the cerebellar nuclei seem to be the most relevant, possibly in conjunction with a projection from the paraventricular nucleus (Dietrichs, 1984; Haines et al., 1997; Zhu et al., 2006; Yu et al., 2010; Çavdar et al., 2018a).

The locus coeruleus provides widespread noradrenergic innervation of the cerebellar cortex and nuclei (Olson and Fuxe, 1971; Saigal et al., 1980a; Loughlin et al., 1986; Dietrichs, 1988; Szabadi, 2013), which could contribute to a general modulation of cerebellar output (Parfitt et al., 1988; Di Mauro et al., 2013; Lippiello et al., 2015). Also both the dorsal and caudal raphe nuclei project to the cerebellar cortex and nuclei, providing serotonergic input (Shinnar et al., 1975; Pierce et al., 1977; Bishop and Ho, 1985; Fu et al., 2011), which can have profound impact on cerebellar function (Kawashima, 2018). In mild forms of cerebellar ataxia, application of a serotonin receptor agonist could alleviate various motor symptoms (Takei et al., 2005). These, and the other anatomical projections, are summarized in Figure 7.

### Cerebellar pathology

**Figure 8.**
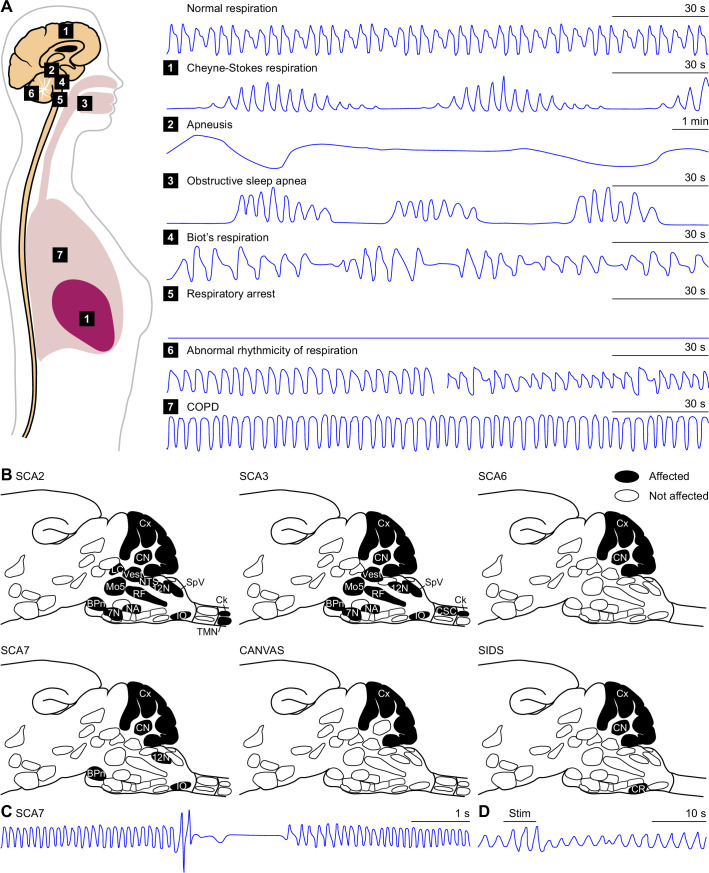
Pathology. (**A**) Based on the location of a lesion or structural abnormality, several types of disordered breathing can be expected. Cheyne-Stokes respiration can result from a bilateral hemispheric or diencephalic lesion. Apneustic breathing has originally been described as the consequence of a lesion at the level of the Kölliker-Fuse nucleus, but can probably also be caused by damage to surrounding tissue. A Biot’s respiration pattern can develop after a lesion in the medulla, while a respiratory arrest occurs after damage to the pre-Bötzinger complex or the upper spinal cord. Obstructive sleep apnea typically shows interrupted breathing pattern during sleep. Cerebellar dysfunction can lead to more (left) or less (right) rhythmic respiration. COPD patients show increased respiratory rates and reduced passive expiration. It should be noted however that cardiopulmonary or metabolic causes can also trigger all these respiratory patterns. Schematized traces of the tidal volume based on: Cheyne-Stokes respiration: Lorenzi-Filho et al., 1999; Apneusis: Mador and Tobin, 1990; Biot’s respiration: Farney et al., 2003: Abnormal rhythmicity: Ebert et al., 1995; COPD (and normal): Jolley et al., 2009. (**B**) Respiration-related brain areas demonstrating structural damage in several disorders, projected in the same notation as in Figure 2A. Note that SCA6 is mainly a cerebellar disorder. While CANVAS affects the cerebellum, the syndrome has also peripheral nerve involvement. In SIDS multiple non-cerebellar causes have been described in post mortem studies. Some post mortem brain studies, however, have shown irregularly formed Purkinje cells in the cerebellar cortex. See Supplementary file 1. (**C**) Apneustic breathing pattern in a mouse model of spinocerebellar ataxia type 7. Schematic representation based on Fusco et al., 2021. (**D**) Electrical stimulation (Stim) adjacent to the fastigial nucleus induces a fastigial pressor response in an anesthetized cat. During stimulation, the tidal volume is increased, and suppressed afterwards. Based on Miura and Takayama, 1988.

Although the role of the cerebellum in respiratory control is not yet settled in clinical settings, several lines of evidence indicate a specific role in respiratory control. For instance, in some developmental disorders, cerebellar abnormalities and respiratory dysfunction co-occur. Arguably, the most explicit example is sudden infant death syndrome (SIDS), the unexplained abrupt death of infants, typically during sleep, and most likely related to respiratory problems (Kelly et al., 1986; Kinney and Thach, 2009). Several studies link SIDS to cerebellar malformations. For instance, post-mortem analyses relate SIDS to the presence of a wide external granular layer, which is a sign of developmental delay of the cerebellum (Cruz-Sánchez et al., 1997; Lavezzi et al., 2007a). Also malformed and displaced Purkinje cells were observed in several cases (Lavezzi et al., 2013; Matschke et al., 2020). Additionally, the dentate-olivary system can be affected in SIDS (Lavezzi et al., 2007b). Note that malformations of the cerebellum and related structures have been demonstrated only in a subset of cases, as SIDS can have multiple causes (Kinney and Thach, 2009; Figure 8B).

Nevertheless, respiratory deficiencies and prolonged dependency on assisted or even mechanical ventilation are frequently observed after cerebellar damage (Chen et al., 2005; Tsitsopoulos et al., 2012; Lee et al., 2013; Arnone et al., 2017). Relatively mild aberrations in respiratory functions were also noted in different types of spinocerebellar ataxia (Sriranjini et al., 2010), while in a mouse model for spinocerebellar ataxia type 7 (SCA7) abnormal respiratory patterns were recorded (Figure 8B–C). Distinct types of spinocerebellar ataxia can affect various brain regions relevant for subconscious control of respiration (Figure 8B, Supplementary file 1). Finally, chronic cough may be one of the earliest symptoms of cerebellar ataxia with neuropathy and vestibular areflexia syndrome (CANVAS), and manifests in the absence of any perceived lung abnormalities (Infante et al., 2018).

## Disordered breathing

Lesions, e.g., as a consequence of hemorrhage, at different levels of the brain can result in different patterns of disordered breathing (Figure 8A).

A bilateral hemispheric or diencephalic lesion can lead to Cheyne-Stokes respiration (Brown and Plum, 1961). This is characterized by an undulating pattern of breathing with increasing depth and frequency alternated with waves of shallow, slower breathing (Lorenzi-Filho et al., 1999).

Apneustic breathing, characterized by the absence or extreme delay of the inspiration to expiration switch, has originally been described as the consequence of a lesion at the level of the Kölliker-Fuse nucleus or the surrounding area (Marckwald, 1890; Lumsden, 1923; Caille et al., 1981; Mador and Tobin, 1990).

Biot’s respiration is characterized by irregular periods of apnea alternated by several breaths of identical depth (Farney et al., 2003; Wijdicks, 2007). Although, the irregularity of this breathing pattern has led to the more common term ‘ataxic breathing’, the cerebellum is not primarily involved in this type of respiration (Summ et al., 2022). However, Biot’s respiration pattern can result after a lesion in the medulla (Summ et al., 2022). Biot’s respiration can also be a complication of long-term opioid use (Farney et al., 2003).

Finally, a respiratory arrest can result after damage to the pre-Bötzinger complex or the upper spinal cord (Ramirez et al., 1998; Schwarzacher et al., 2011; Arora et al., 2012).

Animal experiments have demonstrated that a lack of cerebellar output can lead to hyperregular respiration (Liu et al., 2020; Taylor et al., 2022). Cerebellar patients, however, can also display the opposite and present with irregular respiration (Ebert et al., 1995). Both, hyperregular as well as irregular breathing, can be the consequence of impaired coordination of respiration with ongoing behavior, as described for instance by Ebert et al., 1995.

While each of these brain areas result in a specific type of respiratory pattern. It must be noted that a specific respiratory pattern can have multiple causes such as metabolic or cardiopulmonary disorders. In addition, also peripheral diseases, like COPD (Jolley et al., 2009), can cause disordered breathing. COPD is a chronic obstructive pulmonary condition characterized by abnormalities, especially narrowing of the small airways, of the lung which leads to limitation of the airflow.

## Coordination of different behaviors

Throughout this review, we have discussed several behaviors that are tightly coupled to respiration, such as those involved in airway clearance, feeding, and vocalization, as well as adjusting the rate of ventilation to metabolic demands. In addition, also postural control and cardiovascular function are linked to respiration. All pump muscles have dual functions; they do not only enable ventilation but control also posture and movements (Ebert et al., 2000). The coordination between these different tasks can be affected in cerebellar patients, displaying regular breathing at rest, but arhythmic breathing during arm movements (Ebert et al., 1995).

### Brain rhythms

Global and local rhythms abound in the brain, and they have been proposed to arrange long-range synchrony and functional coupling, and thus play a vital role in motor control, sensory perception, and conscious processing (Llinás, 1988; Khoshyomn et al., 1999; Mantini et al., 2007; Fries, 2015; Lindeman et al., 2021). There is accumulating evidence that the respiratory rhythm can affect these brain rhythms.

In the olfactory system, a coupling between respiration and rhythmic activity was noticed already a long time ago (Adrian, 1942; Fontanini et al., 2003). As odorants are carried by air, each sniff brings in new information, and can be considered as the temporal unit of olfaction (Kepecs et al., 2006; Verhagen et al., 2007). Sniffing can, however, be more widely seen as patterning sensory processing, as it, for instance, can dynamically synchronize with whisking (Welker, 1964; Cao et al., 2012a; Moore et al., 2013). A hint that respiration is even more related to brain function comes from the observation that humans tend to inhale at the start of a cognitive test, even if that does not involve olfaction (Perl et al., 2019). This goes so far that a change in performance on a visuospatial task varies between inspiration and expiration was observed in line with alteration in the EEG spectrum (Perl et al., 2019). In fact, respiration-induced patterns in neural oscillations are ubiquitously observed throughout the brain (Ito et al., 2014; Yanovsky et al., 2014; Heck et al., 2016; Zelano et al., 2016; Tort et al., 2018; Kluger and Gross, 2021).

Changes in brain oscillations are by no means restricted to the frequency domain of respiration or sniffing. Gamma oscillations (30–100 Hz), which could reflect sensorimotor integration (Zagha et al., 2013; Lindeman et al., 2021), can show changes in power that are phase-locked to the respiratory rhythm (Ito et al., 2014; Kluger and Gross, 2021). Respiration can, therefore, serve as a scaffold for sensory, sensorimotor and cognitive functions.

### Cardiorespiratory function

Ventilation is only one aspect of the ultimate goal of respiration: getting oxygen to mitochondria throughout the body, while maintaining balanced concentrations of O_2_ and CO_2_ in the blood. Hence, respiration has to be coordinated with cardiac and vascular regulation, a process collectively known as cardiorespiratory function. Ineffective cardiorespiratory function is an important predictor for development of averse cardiovascular events and mortality, and has a higher predictive value than for example, smoking or hypertension (Lee et al., 2010; Ross et al., 2016).

An example of the integrated control of cardiovascular and respiratory function is the fastigial pressor response that entails simultaneous increases in heart rate and arterial pressure with changes in ventilation (Miura and Reis, 1969; Achari and Downman, 1970; Lutherer and Williams, 1986; Bradley et al., 1987; Xu and Frazier, 2000; Hernandez et al., 2004; Nisimaru, 2004). The specificity of the fastigial nucleus for triggering the fastigial pressor response has been called into question, arguing that it was actually caused by activation of the passing afferents to the lateral parabrachial nucleus (Miura and Takayama, 1988). Yet, lesioning of the fastigial nuclei does lead to impairment of the fastigial pressor response (Zhuang et al., 2008; Figure 8D).

Cardiac and respiratory regulation are coupled on a cycle-by-cycle basis, as the heart rate increases during inspiration, and decreases again during post-inspiration and active expiration (Hirsch and Bishop, 1981; Elstad et al., 2018). Respiratory sinus arrhythmia is caused by respiration-related fluctuations in activity of inhibitory preganglionic parasympathetic cardiac vagal neurons that are primarily located in the nucleus ambiguus (McAllen and Spyer, 1976; Neff et al., 2003; Dergacheva et al., 2010). The Kölliker-Fuse nucleus provides the phasic excitatory drive to these cardiac premotor neurons that is required for respiratory sinus arrhythmia (Farmer et al., 2016).

Stimulation of pro-opiomelanocortin-producing neurons in the NTS can augment respiratory sinus arrhythmia, along with triggering suppressed breathing (bradypnea) and cardiac function (bradycardia) (Cerritelli et al., 2016). Pro-opiomelanocortin is a precursor of the opioid ß-endorphin, and its impact of cardiovascular function can be mimicked by administered opioids (Walker et al., 2007; Izrailtyan et al., 2018). The pro-opiomelanocortin neurons of the NTS directly target important respiratory centers as the pre-Bötzinger complex, giving rise to bradypnea, the ambiguus nucleus, giving rise to bradycardia, and the hypoglossal nucleus, rVRG, hypoglossal nucleus, and raphe obscurus nucleus (Cerritelli et al., 2016; Figure 5E).

## Ideas and speculations

It is an attractive idea to have one or a few central pattern generator areas that control the respiratory rhythm (Moore et al., 2013; Anderson and Ramirez, 2017). In particular, the pre-Bötzinger complex is essential in this respect, as inspiration depends on it (Smith et al., 1991; Ramirez et al., 1998; Gray et al., 2001; Tan et al., 2008; Schwarzacher et al., 2011; Ashhad and Feldman, 2020; Dhingra et al., 2020). In this view, projections organized in a hierarchical fashion pass the respiratory control from the pattern generator(s) to the respiratory motor neurons. Throughout this review we summarize, however, that many other brain regions, including areas like the cerebellum and the limbic system that are not classically considered to be respiratory areas, can affect respiratory control (Figure 8). As all these areas are interconnected (Figure 7), the picture of an integrated network emerges. Thus, depending on specific behavioral needs, these other brain regions may modulate or even overrule the central pattern generators. This does not contradict the idea of central pattern generators being the main players that control rhythmic respiration, but postulates that these pattern generators together with their downstream areas are themselves embedded in a larger network that allows for flexibility within the respiratory control system. We therefore suggest to view the respiratory control system primarily as an integrated network, rather than a hierarchical system. Consequently, we believe that integration of behavioral conditions will add to the pathogenesis of respiratory disease.

## Additional files

Supplementary file 1. List of studies demonstrating the existence of monosynaptic projections between areas relevant for subconscious control of respiration. Connections that were identified as sparse by the authors of these studies are not listed in this table. Note that the list of neurotransmitters involved is not complete. 1 Tree shrew (*Tupaia belangeri chinensis*); 2 Rufous horseshoe bat (*Rhinolophus rouxi*); 3 Rhesus monkey (*Macaca mulatta*); 4 Crab-eating macaque (*Macaca fascicularis*); 5 Squirrel monkey (*Saimiri sciureus*). 5-HT = serotonin, A = anterograde, aa = amino acids, AAV = adeno associated virus, ACh = acetyl choline, BDA = biotinylated dextran amines, Biotinam = biotinamide, CRH = corticotropin-releasing hormone, CTb = cholera toxin B subunit, cVRG = caudal part of the ventral respiratory group, DA = dopamine, Degen = degeneration, DTR = dextran-Texas red, DY = diamidino yellow, EB = Evans blue, Exc = excitatory (not specified which neurotransmitter), E-phys = electrophysiology, FB = fast blue, FG = fluorogold, FR = fluoro ruby, GABA = γ-aminobutyric acid, GFP = green fluorescent protein, Glu = glutamate, Gly = glycine, HSV = herpes simplex virus, HRP = horseradish peroxidase, Inh = inhibitory (not specified which neurotransmitter), m = muscle, MDJ = nuclei of the meso-diencephalic junction, n = nucleus or nuclei, NA = noradrenaline / norepinephrine, Nbiotin = neurobiotin, NMB = neuromedin B, NT = neurotransmitter, NTS = nucleus tractus solitarii, Optogen = optogenetic stimulation, OXT = oxytocin, PHA-L = *Phaseolus vulgaris* leucoagglutinin, PiCo = post-inspiration complex, PPTg = pedunculopontine tegmental nucleus, PI = propidium iodide, POMC = pro-opiomelanocortin, Pseudorab = Pseudorabies, R = retrograde, rVRG = rostral part of the ventral respiratory group, term = terminalis, TMR = tetramethylrhodamine, VP = vasopressin, WGA = wheat germ agglutinin. b – Respiratory control areas affected by pathology Summary of brain regions involved in respiratory control and affected by selected diseases or syndromes. An area is listed in this table if a study reported damage to that area in a majority of subjects included in that study. Case studies with a single patient are not included in this table.
