# Supplementary material for: The integrated brain network that controls respiration
Source: eLife. 2023 Mar 8;12:e83654. doi: 10.7554/eLife.83654 (PMC9995121; doi:10.7554/eLife.83654)
Supplement: Supplementary file 1. — Connections that were identified as sparse by the authors of these studies are not listed in this table. Note that the list of neurotransmitters involved is not complete. 1 Tree shrew (Tupaia belangeri chinensis); 2 Rufous horseshoe bat (Rhinolophus rouxi); 3 Rhesus monkey (Macaca mulatta); 4 Crab-eating macaque (Macaca fascicularis); 5 Squirrel monkey (Saimiri sciureus). 5-HT = serotonin, A = anterograde, aa = amino acids, AAV = adeno associated virus, ACh = acetyl choline, BDA = biotinylated dextran amines, Biotinam = biotinamide, CRH = corticotropin-releasing hormone, CTb = cholera toxin B subunit, cVRG = caudal part of the ventral respiratory group, DA = dopamine, Degen = degeneration, DTR = dextran-Texas red, DY = diamidino yellow, EB = Evans blue, Exc = excitatory (not specified which neurotransmitter), E-phys = electrophysiology, FB = fast blue, FG = fluorogold, FR = fluoro ruby, GABA = γ-aminobutyric acid, GFP = green fluorescent protein, Glu = glutamate, Gly = glycine, HSV = herpes simplex virus, HRP = horseradish peroxidase, Inh = inhibitory (not specified which neurotransmitter), m = muscle, MDJ = nuclei of the meso-diencephalic junction, n = nucleus or nuclei, NA = noradrenaline / norepinephrine, Nbiotin = neurobiotin, NMB = neuromedin B, NT = neurotransmitter, NTS = nucleus tractus solitarii, Optogen = optogenetic stimulation, OXT = oxytocin, PHA-L = Phaseolus vulgaris leucoagglutinin, PiCo = post-inspiration complex, PPTg = pedunculopontine tegmental nucleus, PI = propidium iodide, POMC = pro-opiomelanocortin, Pseudorab = Pseudorabies, R = retrograde, rVRG = rostral part of the ventral respiratory group, term = terminalis, TMR = tetramethylrhodamine, VP = vasopressin, WGA = wheat germ agglutinin. b – Respiratory control areas affected by pathology Summary of brain regions involved in respiratory control and affected by selected diseases or syndromes. An area is listed in this table if a study reported damage to that area in a majority of subjects inclu [file elife-83654-supp1.docx]

| Source area | Target area | NT | Tracer | A/R | Species | Reference |
| --- | --- | --- | --- | --- | --- | --- |
| Carotid bodies | NTS | Glu | WGA-HRP | A | Cat | (Claps and Torrealba, 1988) |
|  |  |  | WGA-HRP | A | Rat | (Finley and Katz, 1992) |
|  | cVRG | Glu | WGA-HRP | A | Rat | (Finley and Katz, 1992) |
| Intercostal m. spindles | Clarke’s column | Glu | HRP | A | Cat | (Nakayama et al., 1998) |
| Nasal mucosa | Sp9inal trigeminal n. | Glu | HRP | A | Cat | (Lucier and Egizii, 1986) |
|  |  |  | WGA-HRP | A | Rat | (Anton and Peppel, 1991) |
| Semicircular canals | Cerebellar cortex | Glu | ^3^H aa | A | Monkey^5^ | (Carleton and Carpenter, 1984) |
|  | Vestibular nuclei | Glu |  |  | Cat | (Gacek, 1969) |
|  |  |  | HRP | A | Cat | (Mannen et al., 1982) |
|  |  |  | ^3^H aa | A | Monkey^5^ | (Carleton and Carpenter, 1984) |
| Ambiguus nucleus | Bötzinger complex |  | WGA-HRP | R | Cat | (Gang et al., 1995) |
|  | Reticular formation |  | HRP | R | Bat^2^ | (Rübsamen and Schweizer, 1986) |
|  | Lateral parabrachial n. |  | HRP | R | Bat^2^ | (Rübsamen and Schweizer, 1986) |
|  | Medial parabrachial n. |  | HRP | R | Bat^2^ | (Rübsamen and Schweizer, 1986) |
| Trigeminal motor n. | Cerebellar cortex | Glu | HRP | R | Mouse | (Fu et al., 2011) |
| Pre-Bötzinger complex | Phrenic nucleus | Glu | Rabies | R | Mouse | (Wu et al., 2017) |
|  | Facial nucleus | Glu  Gly | AAV | A | Mouse | (Yang and Feldman, 2018) |
|  | Hypoglossal nucleus |  | BDA | A | Rat | (Koshiya et al., 2014) |
|  |  |  | Rabies | R | Mouse | (Guo et al., 2020) |
|  | Bötzinger complex | Glu  Gly | AAV | A | Rat | (Tan et al., 2010) |
|  |  |  | AAV | A | Mouse | (Yang and Feldman, 2018) |
|  | PiCo | Glu  Gly | AAV | A | Mouse | (Yang and Feldman, 2018) |
|  | Lateral parafacial n. | Glu  Gly  GABA | AAV | A | Rat | (Tan et al., 2010) |
|  |  |  | AAV | A | Mouse | (Yang and Feldman, 2018) |
|  |  |  | HSV | R | Rat | (Biancardi et al., 2021) |
|  | Retrotrapezoid n. | Glu  Gly | AAV | A | Rat | (Tan et al., 2010) |
|  |  |  | AAV | A | Mouse | (Yang and Feldman, 2018) |
|  | NTS | Glu  Gly | AAV | A | Rat | (Tan et al., 2010) |
|  |  |  | BDA | A | Rat | (Koshiya et al., 2014) |
|  |  |  | AAV | A | Mouse | (Yang and Feldman, 2018) |
|  | Locus coeruleus | Glu | Rabies | R | Mouse | (Yackle et al., 2017) |
|  | rVRG | Glu  Gly | AAV | A | Rat | (Tan et al., 2010) |
|  |  |  | Rabies | R | Mouse | (Wu et al., 2017) |
|  |  |  | AAV | A | Mouse | (Yang and Feldman, 2018) |
|  | cVRG | Glu  Gly | WGA-HRP | R | Cat | (Gerrits and Holstege, 1996) |
|  |  |  | AAV | A | Rat | (Tan et al., 2010) |
|  |  |  | AAV | A | Mouse | (Yang and Feldman, 2018) |
|  | Reticular formation | Glu  Gly | AAV | A | Rat | (Tan et al., 2010) |
|  |  |  | BDA | A | Rat | (Moore et al., 2013) |
|  |  |  | BDA | A | Rat | (Koshiya et al., 2014) |
|  |  |  | AAV | A | Mouse | (Yang and Feldman, 2018) |
|  |  |  | Rabies | R | Mouse | (Takatoh et al., 2022) |
|  | Lateral hypothalamus | Glu  Gly | AAV | A | Mouse | (Yang and Feldman, 2018) |
|  | Dorsomedial hypothal. | Glu  Gly | AAV | A | Mouse | (Yang and Feldman, 2018) |
|  | Vestibular nuclei |  | BDA | A | Rat | (Koshiya et al., 2014) |
|  | Kölliker-Fuse nucleus | Glu  Gly | AAV | A | Rat | (Tan et al., 2010) |
|  |  |  | AAV | A | Mouse | (Yang and Feldman, 2018) |
|  | Lateral parabrachial n. | Glu  Gly | AAV | A | Rat | (Tan et al., 2010) |
|  |  |  | AAV | A | Mouse | (Yang and Feldman, 2018) |
|  | Medial parabrachial n. | Glu  Gly | AAV | A | Rat | (Tan et al., 2010) |
|  |  |  | AAV | A | Mouse | (Yang and Feldman, 2018) |
|  | Periaqueductal gray | Glu  Gly | AAV | A | Rat | (Tan et al., 2010) |
|  |  |  | AAV | A | Mouse | (Yang and Feldman, 2018) |
|  |  |  | CTb | R | Rat | (Trevizan-Baú et al., 2021b) |
| Bötzinger complex | Phrenic nucleus | Gly | E-phys |  | Cat | (Merrill and Fedorko, 1984) |
|  |  |  | WGA-HRP | R | Rabbit | (Ellenberger et al., 1990b) |
|  |  |  | E-phys |  | Rat | (Tian et al., 1998) |
|  |  |  | Rabies | R | Mouse | (Wu et al., 2017) |
|  | Pre-Bötzinger complex | Gly | Rabies | R | Mouse | (Yang et al., 2020) |
|  | Lateral parafacial n. | Gly  GABA  Glu | HSV | R | Rat | (Biancardi et al., 2021) |
|  | NTS | Gly  GABA | E-phys |  | Cat | (Merrill et al., 1983) |
|  |  |  | E-phys |  | Cat | (Fedorko and Merrill, 1984) |
|  |  |  | HRP | R | Cat | (Livingston and Berger, 1989) |
|  |  |  | Biocytin | A | Rat | (Ezure et al., 2003) |
|  | rVRG | Gly | E-phys |  | Cat | (Jiang and Lipski, 1990) |
|  |  |  | Biocytin | A | Rat | (Bryant et al., 1993) |
|  |  |  | Biocytin | A | Rat | (Ezure et al., 2003) |
|  | cVRG | Gly | E-phys |  | Cat | (Fedorko and Merrill, 1984) |
|  |  |  | E-phys |  | Cat | (Jiang and Lipski, 1990) |
|  |  |  | Biocytin | A | Rat | (Bryant et al., 1993) |
|  |  |  | WGA-HRP | R | Cat | (Gerrits and Holstege, 1996) |
|  |  |  | Biocytin | A | Rat | (Ezure et al., 2003) |
|  | Kölliker-Fuse nucleus | GABA  Gly | Biocytin | A | Rat | (Ezure et al., 2003) |
|  | Lateral parabrachial n. | Gly | Biocytin | A | Rat | (Ezure et al., 2003) |
|  | Periaqueductal gray | Gly | CTb | R | Rat | (Trevizan-Baú et al., 2021b) |
| PiCo | Pre-Bötzinger complex | ACh | Rabies | R | Mouse | (Yang et al., 2020) |
|  | Retrotrapezoid n. | ACh | FG | R | Rat | (Lima et al., 2019) |
| Lateral parafacial n. | cVRG | Glu | FG | R | Rat | (Silva et al., 2016a) |
| Retrotrapezoid n. | Thoracic motor n. | Glu | BDA | A | Rat | (Rosin et al., 2006) |
|  | Pre-Bötzinger complex | Glu  Galanin  GABA  NMB | BDA | A | Rat | (Rosin et al., 2006) |
|  |  |  | BDA | A | Rat | (Bochorishvili et al., 2012) |
|  |  |  | GFP | A | Mouse | (Li et al., 2016) |
|  |  |  | BDA | A | Rat | (Silva et al., 2016a) |
|  | Bötzinger complex | Glu  Galanin | WGA-HRP | R | Cat | (Gang et al., 1995) |
|  |  |  | BDA | A | Rat | (Rosin et al., 2006) |
|  |  |  | BDA | A | Rat | (Bochorishvili et al., 2012) |
|  |  |  | BDA | A | Rat | (Silva et al., 2016a) |
|  | Lateral parafacial n. | Glu  GABA  Gly | CTb | R | Rat | (Zoccal et al., 2018) |
|  |  |  | HSV | A | Rat | (Biancardi et al., 2021) |
|  | NTS | Glu  Galanin | BDA | A | Rat | (Rosin et al., 2006) |
|  |  |  | BDA | A | Rat | (Bochorishvili et al., 2012) |
|  | rVRG | Glu  Galanin | BDA | A | Rat | (Rosin et al., 2006) |
|  |  |  | BDA | A | Rat | (Bochorishvili et al., 2012) |
|  |  |  | BDA | A | Rat | (Silva et al., 2016a) |
|  | cVRG | Glu  GABA  Galanin | WGA-HRP | R | Cat | (Gerrits and Holstege, 1996) |
|  |  |  | BDA | A | Rat | (Rosin et al., 2006) |
|  |  |  | BDA | A | Rat | (Bochorishvili et al., 2012) |
|  |  |  | BDA | A | Rat | (Silva et al., 2016a) |
|  |  |  | FG | R | Rat | (Silva et al., 2016a) |
|  | Cervical spinal cord | Glu | BDA | A | Rat | (Rosin et al., 2006) |
|  | Kölliker-Fuse nucleus | Glu  GABA  Galanin | BDA | A | Rat | (Rosin et al., 2006) |
|  |  |  | BDA | A | Rat | (Bochorishvili et al., 2012) |
|  |  |  | BDA | A | Rat | (Silva et al., 2016b) |
|  | Lateral parabrachial n. | Glu  Galanin | BDA | A | Rat | (Rosin et al., 2006) |
|  |  |  | BDA | A | Rat | (Bochorishvili et al., 2012) |
|  |  |  | BDA | A | Rat | (Silva et al., 2016b) |
|  | Medial parabrachial n. | Glu | BDA | A | Rat | (Rosin et al., 2006) |
| NTS | Phrenic nucleus | Glu | ^3^H aa | A | Cat | (Loewy and Burton, 1978) |
|  |  |  | HRP | R | Cat | (Rikard-Bell et al., 1984) |
|  |  |  | E-phys |  | Cat | (Fedorko et al., 1983) |
|  |  |  | WGA-HRP | R | Rabbit | (Ellenberger et al., 1990b) |
|  |  |  | Pseudorab | R | Rat | (Dobbins and Feldman, 1994) |
|  |  |  | FG | R | Rat | (Boulenguez et al., 2007) |
|  |  |  | Rabies | R | Cat | (Lois et al., 2009) |
|  |  |  | Rabies | R | Mouse | (Wu et al., 2017) |
|  | Trigeminal motor n. |  | BDA | A | Rat | (Kawai, 2018) |
|  | Facial nucleus |  | ^3^H aa | A | Rat | (Norgren, 1978) |
|  |  |  | BDA | A | Rat | (Kawai, 2018) |
|  | Hypoglossal nucleus | POMC | ^3^H aa | A | Rat | (Norgren, 1978) |
|  |  |  | AAV | A | Mouse | (Cerritelli et al., 2016) |
|  |  |  | Rabies | R | Mouse | (Guo et al., 2020) |
|  | Ambiguus nucleus | Exc  Inh  POMC | ^3^H aa | A | Cat | (Loewy and Burton, 1978) |
|  |  |  | ^3^H aa | A | Rat | (Norgren, 1978) |
|  |  |  | ^3^H aa | A | Rat | (Ricardo and Koh, 1978) |
|  |  |  | ^3^H aa | A | Monkey^4^ | (Beckstead et al., 1980) |
|  |  |  | DY | R | Rat | (Núñez-Abades et al., 1990) |
|  |  |  | WGA-HRP | A | Rat | (Hayakawa et al., 2000) |
|  |  |  | AAV | A | Mouse | (Cerritelli et al., 2016) |
|  |  |  | BDA | A | Rat | (Kawai, 2018) |
|  | Pre-Bötzinger complex | POMC | AAV | A | Mouse | (Cerritelli et al., 2016) |
|  |  |  | Rabies | R | Mouse | (Yang et al., 2020) |
|  | Bötzinger complex |  | WGA-HRP | R | Cat | (Gang et al., 1995) |
|  | PiCo | Glu | FG | R | Rat | (Oliveira et al., 2021) |
|  | Lateral parafacial n. | Glu  GABA  Gly | Biotinam | A | Rat | (Takakura et al., 2007) |
|  |  |  | CTb | R | Rat | (Silva et al., 2019) |
|  |  |  | HSV | R | Rat | (Biancardi et al., 2021) |
|  | Retrotrapezoid n. | Ach | CTb | R | Rat | (Rosin et al., 2006) |
|  |  |  | FG | R | Rat | (Lima et al., 2019) |
|  | Locus coeruleus |  | CTb | A | Rat | (McGovern et al., 2015) |
|  |  |  | BDA | A | Rat | (Kawai, 2018) |
|  | Dorsal raphe |  | CTb | R | Rat | (Peyron et al., 2018) |
|  | Caudal raphe | POMC | AAV | A | Mouse | (Cerritelli et al., 2016) |
|  | Spinal trigeminal n. |  | ^3^H aa | A | Cat | (Loewy and Burton, 1978) |
|  |  |  | CTb | A | Rat | (McGovern et al., 2015) |
|  | rVRG | POMC | E-phys |  | Cat | (Ezure and Tanaka, 1996) |
|  |  |  | Biocytin | R | Rat | (Zheng et al., 1998) |
|  |  |  | AAV | A | Mouse | (Cerritelli et al., 2016) |
|  |  |  | AAV | A | Mouse | (Do et al., 2020) |
|  | cVRG |  | ^3^H aa | A | Cat | (Loewy and Burton, 1978) |
|  |  |  | ^3^H aa | A | Monkey^4^ | (Beckstead et al., 1980) |
|  |  |  | WGA-HRP | R | Cat | (Gerrits and Holstege, 1996) |
|  | Reticular formation | POMC | ^3^H aa | A | Rat | (Ricardo and Koh, 1978) |
|  |  |  | CTb | A | Rat | (McGovern et al., 2015) |
|  |  |  | AAV | A | Mouse | (Cerritelli et al., 2016) |
|  |  |  | BDA | A | Rat | (Kawai, 2018) |
|  | Central amygdala |  | ^3^H aa | A | Rat | (Ricardo and Koh, 1978) |
|  |  |  | CTb | R | Rat | (Bienkowski and Rinaman, 2013) |
|  |  |  | CTb | A | Rat | (McGovern et al., 2015) |
|  |  |  | BDA | A | Rat | (Kawai, 2018) |
|  | Bed n. of stria term. | NA | ^3^H aa | A | Rat | (Ricardo and Koh, 1978) |
|  |  |  | CTb | R | Rat | (Shin et al., 2008) |
|  |  |  | CTb | R | Rat | (Bienkowski and Rinaman, 2013) |
|  |  |  | FG | R | Tree shrew^1^ | (Ni et al., 2016) |
|  |  |  | BDA | A | Rat | (Kawai, 2018) |
|  | Lateral hypothalamus |  | CTb | A | Rat | (McGovern et al., 2015) |
|  |  |  | BDA | A | Rat | (Kawai, 2018) |
|  | Paraventricular n. | GABA  DA | ^3^H aa | A | Rat | (Ricardo and Koh, 1978) |
|  |  |  | FG | R | Rat | (King et al., 2012) |
|  |  |  | CTb | A | Rat | (McGovern et al., 2015) |
|  |  |  | BDA | A | Rat | (Kawai, 2018) |
|  | Dorsomedial hypothal. | Glu | ^3^H aa | A | Rat | (Ricardo and Koh, 1978) |
|  |  |  | BDA | A | Rat | (Kawai, 2018) |
|  |  |  | AAV | A | Mouse | (Do et al., 2020) |
|  | Cerebellar cortex |  | HRP | R | Cat | (Batini et al., 1978) |
|  |  |  | HRP | R | Cat | (Somana and Walberg, 1979a) |
|  |  |  | HRP | R | Sheep | (Saigal et al., 1980a) |
|  |  |  | HRP | R | Mouse | (Fu et al., 2011) |
|  | Inferior olive |  | ^3^H aa | A | Cat | (Loewy and Burton, 1978) |
|  |  |  | CTb | A | Rat | (McGovern et al., 2015) |
|  | Vestibular nuclei |  | Rabies | R | Mouse | (Shi et al., 2021) |
|  | Kölliker-Fuse nucleus | Glu  GABA | ^3^H aa | A | Cat | (Loewy and Burton, 1978) |
|  |  |  | PHA-L | A | Rat | (Herbert et al., 1990) |
|  |  |  | CTb | A | Rat | (McGovern et al., 2015) |
|  | Lateral parabrachial n. |  | ^3^H aa | A | Monkey^4^ | (Beckstead et al., 1980) |
|  |  |  | PHA-L | A | Rat | (Herbert et al., 1990) |
|  |  |  | CTb | A | Rat | (McGovern et al., 2015) |
|  |  |  | BDA | A | Rat | (Kawai, 2018) |
|  |  |  | AAV | A | Mouse | (Yu et al., 2021) |
|  |  |  | AAV | R | Mouse | (Yu et al., 2021) |
|  | Medial parabrachial n. |  | ^3^H aa | A | Cat | (Loewy and Burton, 1978) |
|  |  |  | PHA-L | A | Rat | (Herbert et al., 1990) |
|  |  |  | CTb | A | Rat | (McGovern et al., 2015) |
|  |  |  | FB | R | Mouse | (Hashimoto et al., 2018) |
|  | Periaqueductal gray | NA | FB / FG | R | Rat | (Herbert and Saper, 1992) |
|  |  |  | PHA-L | A | Rat | (Herbert and Saper, 1992) |
|  |  |  | BDA | A | Rat | (Kawai, 2018) |
|  | PPTg |  | WGA-HRP | R | Rat | (Steininger et al., 1992) |
| Locus coeruleus | Phrenic nucleus | NA | AAV | A | Rat | (Bruinstroop et al., 2012) |
|  | Thoracic spinal cord | NA | AAV | A | Rat | (Bruinstroop et al., 2012) |
|  | Facial nucleus | NA | ^3^H-leucine | A | Rat | (Jones and Yang, 1985) |
|  | Hypoglossal nucleus | NA | ^3^H-leucine | A | Rat | (Jones and Yang, 1985) |
|  | Ambiguus nucleus | NA | ^3^H-leucine | A | Rat | (Jones and Yang, 1985) |
|  | Pre-Bötzinger complex | NA | AAV | A | Mouse | (Liu et al., 2021b) |
|  |  |  | AAV | R | Mouse | (Liu et al., 2021b) |
|  | Dorsal raphe | NA | ^3^H-leucine | A | Rat | (Jones and Yang, 1985) |
|  | Caudal raphe | NA | ^3^H aa | A | Rat | (Jones and Moore, 1977) |
|  |  |  | ^3^H-leucine | A | Rat | (Jones and Yang, 1985) |
|  |  |  | CTb | R | Rat | (Hermann et al., 1997) |
|  | Cervical spinal cord | NA | HRP | R | Monkey^4^ | (Westlund and Coulter, 1980) |
|  |  |  | True blue | R | Rat | (Jones and Yang, 1985) |
|  |  |  | AAV | A | Rat | (Bruinstroop et al., 2012) |
|  | Central amygdala | NA | ^3^H aa | A | Rat | (Jones and Moore, 1977) |
|  |  |  | ^3^H-leucine | A | Rat | (Jones and Yang, 1985) |
|  |  |  | Dextran | R | Rat | (Borodovitsyna et al., 2020) |
|  | Bed n. stria term. | NA | FG | R | Tree shrew^1^ | (Ni et al., 2016) |
|  | Lateral hypothalamus | NA | ^3^H aa | A | Rat | (Jones and Moore, 1977) |
|  | Paraventricular n. | NA | ^3^H aa | A | Rat | (Jones and Moore, 1977) |
|  |  |  | ^3^H-leucine | A | Rat | (Jones and Yang, 1985) |
|  |  |  | PHA-L | A | Rat | (Cunningham and Sawchenko, 1988) |
|  | Cerebellar cortex | NA | Degen | R | Rat | (Olson and Fuxe, 1971) |
|  |  |  | HRP | R | Sheep | (Saigal et al., 1980b) |
|  |  |  | EB | R | Rat | (Room et al., 1981) |
|  |  |  | Primuline | R | Rat | (Nagai et al., 1981) |
|  |  |  | HRP | R | Rat | (Loughlin et al., 1986) |
|  |  |  | WGA-HRP | R | Cat | (Dietrichs, 1988) |
|  |  |  | HRP | R | Mouse | (Fu et al., 2011) |
|  | Fastigial nucleus | NA | FG | R | Rat | (Ruggiero et al., 1997) |
|  |  |  | WGA-HRP | R | Cat | (Dietrichs, 1988) |
|  | Interposed nucleus | NA | WGA-HRP | R | Cat | (Dietrichs, 1988) |
|  | Dentate nucleus | NA | WGA-HRP | R | Cat | (Dietrichs, 1988) |
|  | MDJ | NA | ^3^H-leucine | A | Rat | (Jones and Yang, 1985) |
|  | Vestibular nuclei |  | Rabies | R | Mouse | (Shi et al., 2021) |
|  | Lateral parabrachial n. | NA  Glu | GFP | A | Mouse | (Robertson et al., 2013) |
|  |  |  | E-phys |  | Mouse | (Yang et al., 2021) |
|  | Medial parabrachial n. | NA | GFP | A | Mouse | (Robertson et al., 2013) |
|  | Periaqueductal gray | NA | ^3^H aa | A | Rat | (Jones and Moore, 1977) |
|  |  |  | ^3^H-leucine | A | Rat | (Jones and Yang, 1985) |
|  | PPTg | NA | ^3^H-leucine | A | Rat | (Jones and Yang, 1985) |
| Dorsal raphe | Trigeminal motor n. | 5-HT | FG | R | Rat | (Li et al., 1993) |
|  | Facial nucleus | 5-HT | FG | R | Rat | (Li et al., 1993) |
|  | Hypoglossal nucleus | 5-HT | Rabies | R | Mouse | (Guo et al., 2020) |
|  | Retrotrapezoid n. | 5-HT | CTb | R | Rat | (Rosin et al., 2006) |
|  | Locus coeruleus | 5-HT | CTb | R | Rat | (Luppi et al., 1995) |
|  |  |  | Rabies | R | Mouse | (Schwarz et al., 2015) |
|  | Reticular formation | 5-HT | PHA-L | A | Rat | (Vertes, 1991) |
|  | Central amygdala | 5-HT | PHA-L | A | Rat | (Vertes, 1991) |
|  |  |  | FB | R | Rat | (Halberstadt and Balaban, 2006) |
|  |  |  | CTb | R | Rat | (Bienkowski and Rinaman, 2013) |
|  | Bed n. of stria term. | 5-HT  DA | PHA-L | A | Rat | (Vertes, 1991) |
|  |  |  | CTb | R | Rat | (Shin et al., 2008) |
|  |  |  | CTb | R | Rat | (Bienkowski and Rinaman, 2013) |
|  |  |  | FG | R | Tree shrew^1^ | (Ni et al., 2016) |
|  | Lateral hypothalamus | 5-HT  GABA | PI/TB/EB | R | Rat | (Woolf and Butcher, 1986) |
|  |  |  | PHA-L | A | Rat | (Vertes, 1991) |
|  |  |  | CTb | R | Rat | (Yoshida et al., 2006) |
|  |  |  | FG | R | Mouse | (Bang and Commons, 2012) |
|  | Vestibular nuclei | 5-HT | DY | R | Rat | (Halberstadt and Balaban, 2006) |
|  |  |  | Rabies | R | Mouse | (Shi et al., 2021) |
|  | Lateral parabrachial n. | 5-HT | Microbead | R | Rat | (Petrov et al., 1992) |
|  |  |  | Microbead | R | Cat | (Quattrochi et al., 1998) |
|  |  |  | AAV | A | Mouse | (Kaur et al., 2020) |
|  |  |  | CTb | R | Mouse | (Kaur et al., 2020) |
|  | Periaqueductal gray | 5-HT | PHA-L | A | Rat | (Vertes, 1991) |
|  | PPTg | 5-HT | PHA-L | A | Rat | (Vertes, 1991) |
|  |  |  | WGA-HRP | R | Rat | (Steininger et al., 1992) |
| Caudal raphe  (Medullary raphe, consisting of raphe magnus,  raphe pallidus and  raphe obscurus) | Phrenic nucleus | 5-HT | AAV | A | Mouse | (Depuy et al., 2011) |
|  |  |  | GFP | A | Mouse | (Hennessy et al., 2017) |
|  | Thoracic spinal cord | 5-HT | AAV | A | Mouse | (Depuy et al., 2011) |
|  | Trigeminal motor n. | 5-HT | FG | R | Rat | (Li et al., 1993) |
|  |  |  | AAV | A | Mouse | (Depuy et al., 2011) |
|  |  |  | GFP | A | Mouse | (Hennessy et al., 2017) |
|  | Facial nucleus | 5-HT | FG | R | Rat | (Li et al., 1993) |
|  |  |  | AAV | A | Mouse | (Depuy et al., 2011) |
|  |  |  | GFP | A | Mouse | (Hennessy et al., 2017) |
|  | Hypoglossal nucleus | 5-HT | FG | R | Rat | (Li et al., 1993) |
|  |  |  | AAV | A | Mouse | (Depuy et al., 2011) |
|  |  |  | GFP | A | Mouse | (Hennessy et al., 2017) |
|  |  |  | Rabies | R | Mouse | (Guo et al., 2020) |
|  | Ambiguus nucleus | 5-HT | AAV | A | Mouse | (Depuy et al., 2011) |
|  |  |  | GFP | A | Mouse | (Hennessy et al., 2017) |
|  | Pre-Bötzinger complex | 5-HT | AAV | A | Mouse | (Depuy et al., 2011) |
|  |  |  | GFP | A | Mouse | (Brust et al., 2014) |
|  |  |  | GFP | A | Mouse | (Hennessy et al., 2017) |
|  | Lateral parafacial n. | 5-HT  Glu  GABA  Gly | HSV | R | Rat | (Biancardi et al., 2021) |
|  | Retrotrapezoid n. | 5-HT | CTb | R | Rat | (Rosin et al., 2006) |
|  |  |  | GFP | A | Mouse | (Brust et al., 2014) |
|  | NTS | 5-HT | GFP | A | Mouse | (Brust et al., 2014) |
|  |  |  | GFP | A | Mouse | (Hennessy et al., 2017) |
|  | Locus coeruleus | 5-HT | GFP | A | Mouse | (Brust et al., 2014) |
|  | Cerebellar cortex | 5-HT  non-5-HT | HRP | R | Cat | (Shinnar et al., 1975) |
|  |  |  | HRP | R | Cat | (Pierce et al., 1977) |
|  |  |  | HRP | A | Rat | (Takagi et al., 1981) |
|  |  |  | HRP | R | Rat | (Bishop and Ho, 1985) |
|  |  |  | HRP | R | Mouse | (Fu et al., 2011) |
|  | Fastigial nucleus | 5-HT | HRP | R | Cat | (Pierce et al., 1977) |
|  | Interposed nucleus | 5-HT | HRP | R | Cat | (Pierce et al., 1977) |
|  | Medial parabrachial n. | 5-HT | GFP | A | Mouse | (Brust et al., 2014) |
|  | Periaqueductal gray | 5-HT  non-5-HT | HRP | A | Rat | (Takagi et al., 1981) |
|  |  |  | FB / FG | R | Rat | (Herbert and Saper, 1992) |
|  |  |  | CTB | R | Rat | (Trevizan-Baú et al., 2021b) |
|  | PPTg | 5-HT | WGA-HRP | R | Rat | (Semba and Fibiger, 1992) |
| Paratrigeminal nucleus | Ambiguus nucleus |  | Biocytin | A | Rat | (Caous et al., 2001) |
|  |  |  | Biocytin | A | Rat | (de Sousa Buck et al., 2001) |
|  | NTS |  | WGA-HRP | R | Rat | (Menétrey et al., 1987) |
|  |  |  | TMR dextran | A | Rat | (Saxon and Hopkins, 1998) |
|  |  |  | FG | R | Rat | (Saxon and Hopkins, 1998) |
|  |  |  | FG | R | Rat | (de Sousa Buck et al., 2001) |
|  |  |  | CTb | A | Rat | (McGovern et al., 2015) |
|  |  |  | BDA | A | Guinea pig | (Driessen et al., 2018) |
|  | Spinal trigeminal n. |  | TMR dextran | A | Rat | (Saxon and Hopkins, 1998) |
|  |  |  | CTb | A | Rat | (McGovern et al., 2015) |
|  |  |  | BDA | A | Guinea pig | (Driessen et al., 2018) |
|  | Reticular formation |  | Biocytin | A | Rat | (Caous et al., 2001) |
|  |  |  | CTb | A | Rat | (McGovern et al., 2015) |
|  | Cerebellar cortex |  | HRP | R | Cat | (Somana and Walberg, 1979b) |
|  | Fastigial nucleus |  | HRP | R | Cat | (Somana and Walberg, 1979b) |
|  | Interposed nucleus |  | HRP | R | Cat | (Somana and Walberg, 1979b) |
|  | Inferior olive |  | CTb | A | Rat | (McGovern et al., 2015) |
|  | Kölliker-Fuse nucleus |  | TMR dextran | A | Rat | (Saxon and Hopkins, 1998) |
|  |  |  | CTb | A | Rat | (McGovern et al., 2015) |
|  |  |  | BDA | A | Guinea pig | (Driessen et al., 2018) |
|  | Lateral parabrachial n. |  | TMR dextran | A | Rat | (Saxon and Hopkins, 1998) |
|  |  |  | Biocytin | A | Rat | (Caous et al., 2001) |
|  |  |  | CTb | A | Rat | (McGovern et al., 2015) |
|  |  |  | BDA | A | Guinea pig | (Driessen et al., 2018) |
|  | Medial parabrachial n. |  | TMR dextran | A | Rat | (Saxon and Hopkins, 1998) |
|  |  |  | Biocytin | A | Rat | (Caous et al., 2001) |
|  |  |  | CTb | A | Rat | (McGovern et al., 2015) |
|  |  |  | BDA | A | Guinea pig | (Driessen et al., 2018) |
|  |  |  | FB | R | Mouse | (Hashimoto et al., 2018) |
|  | Periaqueductal gray |  | CTb | A | Rat | (McGovern et al., 2015) |
| Spinal trigeminal n. | Facial nucleus | Glu | BDA | A | Rat | (Panneton et al., 2006) |
|  |  |  | AAV | A | Rat | (Elbaz et al., 2022) |
|  | Pre-Bötzinger complex | Glu | BDA | A | Rat | (Panneton et al., 2006) |
|  | Lateral parafacial n. | Glu | HSV | R | Rat | (Biancardi et al., 2021) |
|  | NTS | Glu | BDA | A | Rat | (Panneton et al., 2006) |
|  | cVRG | Glu | GFP | A | Mouse | (Li et al., 2021) |
|  | Reticular formation | Glu | BDA | A | Rat | (Dauvergne et al., 2001) |
|  |  |  | AAV | A | Rat | (Elbaz et al., 2022) |
|  | Cerebellar cortex | Glu | HRP | R | Sheep | (Saigal et al., 1980a) |
|  |  |  | WGA-HRP | A | Rabbit | (Van Ham and Yeo, 1992) |
|  |  |  | WGA-HRP  Biocytin  BDA | A | Rat | (Yatim et al., 1996) |
|  |  |  | WGA-HRP | R | Rat | (Yatim et al., 1996) |
|  |  |  | HRP | R | Mouse | (Fu et al., 2011) |
|  |  |  | CTb | R | Mouse | (Henschke and Pakan, 2020) |
|  |  |  | AAV | A | Rat | (Elbaz et al., 2022) |
|  | Basal pons | Glu | WGA-HRP | R | Rat | (Mihailoff et al., 1989) |
|  | MDJ | Glu | FG | R | Mouse | (Kubo et al., 2018) |
|  | Inferior olive | Glu | HRP | R | Rat | (Swenson and Castro, 1983) |
|  |  |  | ^3^H aa | A | Cat | (Huerta et al., 1985) |
|  |  |  | WGA-HRP | R | Rabbit | (Van Ham and Yeo, 1992) |
|  |  |  | WGA-HRP  Biocytin  BDA | A | Rat | (Yatim et al., 1996) |
|  |  |  | WGA-HRP | A | Rat | (Molinari et al., 1996) |
|  |  |  | BDA | A | Rat | (Panneton et al., 2006) |
|  | Vestibular nuclei | Glu | BDA  Biocytin | A | Rat | (Buisseret-Delmas et al., 1999) |
|  |  |  | Rabies | R | Mouse | (Shi et al., 2021) |
|  | Kölliker-Fuse nucleus | Glu | BDA | A | Rat | (Panneton et al., 2006) |
|  |  |  | BDA | A | Rat | (Zhang et al., 2018) |
|  |  |  | AAV | A | Rat | (Elbaz et al., 2022) |
|  | Lateral parabrachial n. | Glu | BDA | A | Rat | (Panneton et al., 2006) |
|  |  |  | BDA | A | Rat | (Zhang et al., 2018) |
|  |  |  | FG | R | Rat | (Zhang et al., 2018) |
|  | Medial parabrachial n. | Glu | FB | R | Mouse | (Hashimoto et al., 2018) |
|  | Periaqueductal gray | Glu | WGA-HRP | R | Cat | (Wiberg et al., 1986) |
|  |  |  | WGA-HRP | R | Rat | (Beitz, 1989) |
| Clarke’s column | Cerebellar cortex | Glu | HRP |  | Cat | (Matsushita et al., 1979) |
|  |  |  | HRP | R | Rat | (Matsushita and Hosoya, 1979) |
|  |  |  | tdTomato | A | Mouse | (Pop et al., 2022) |
|  |  |  | FG | R | Mouse | (Pop et al., 2022) |
| rVRG | Phrenic nucleus | Glu | PHA-L | A | Rat | (Ellenberger and Feldman, 1988) |
|  |  |  | WGA-HRP | R | Rat | (Ellenberger et al., 1990a) |
|  |  |  | Nbiotin | A | Rat | (Lipski et al., 1994) |
|  |  |  | E-phys |  | Rat | (Tian and Duffin, 1996) |
|  |  |  | FG | R | Rat | (Boulenguez et al., 2007) |
|  |  |  | Rabies | R | Mouse | (Wu et al., 2017) |
|  | Facial nucleus | Glu | PHA-L | A | Rat | (Yamada et al., 1988) |
|  |  |  | WGA-HRP | R | Rat | (Ellenberger et al., 1990a) |
|  |  |  | Biocytin | A | Rat | (Zheng et al., 1998) |
|  | Hypoglossal nucleus | Glu | PHA-L | A | Rat | (Yamada et al., 1988) |
|  |  |  | Biocytin | A | Rat | (Zheng et al., 1998) |
|  |  |  | Nbiotin | A | Rat | (Lipski et al., 1994) |
|  | Ambiguus nucleus | Glu | PHA-L | A | Rat | (Yamada et al., 1988) |
|  | Lateral parafacial n. | Glu  Gly  GABA | HSV | R | Rat | (Biancardi et al., 2021) |
|  | Retrotrapezoid n. |  | CTb | R | Rat | (Rosin et al., 2006) |
|  | NTS | Glu | PHA-L | A | Rat | (Yamada et al., 1988) |
|  |  |  | WGA-HRP | R | Rat | (Ellenberger et al., 1990a) |
|  |  |  | Biocytin | A | Rat | (Zheng et al., 1998) |
|  | Spinal trigeminal n. |  | Biocytin | A | Rat | (Zheng et al., 1998) |
|  | cVRG | Glu | PHA-L | A | Rat | (Ellenberger and Feldman, 1990) |
|  |  |  | WGA-HRP | R | Cat | (Gerrits and Holstege, 1996) |
|  | Reticular formation |  | Biocytin | A | Rat | (Zheng et al., 1998) |
|  | Cervical spinal cord | Glu | Nbiotin | A | Rat | (Lipski et al., 1994) |
|  |  |  | E-phys |  | Rat | (Tian and Duffin, 1996) |
|  | Cerebellar cortex | Glu | FR | A | Rat | (Gaytán and Pásaro, 1998) |
|  | Fastigial nucleus | Glu | FR | A | Rat | (Gaytán and Pásaro, 1998) |
|  | Interposed nucleus | Glu | FR | A | Rat | (Gaytán and Pásaro, 1998) |
|  | Dentate nucleus | Glu | FR | A | Rat | (Gaytán and Pásaro, 1998) |
|  | Inferior olive |  | HRP | R | Rat | (Swenson and Castro, 1983) |
|  | Kölliker-Fuse nucleus | Glu | PHA-L | A | Rat | (Ellenberger et al., 1990b) |
|  |  |  | Nbiotin | A | Rat | (Lipski et al., 1994) |
|  |  |  | FG | R | Rat | (Yokota et al., 2016) |
|  | Lateral parabrachial n. | Glu | PHA-L | A | Rat | (Yamada et al., 1988) |
|  |  |  | WGA-HRP | R | Rat | (Ellenberger et al., 1990a) |
| cVRG | Thoracic spinal cord |  | HRP | R | Cat | (Holstege, 1989) |
|  |  |  | HRP | A | Rat | (Ezure et al., 2003) |
|  |  |  | WGA-HRP | A | Cat | (Boers et al., 2006) |
|  | Trigeminal motor n. |  | BDA | A | Rat | (Jones et al., 2016) |
|  | Facial nucleus |  | ^3^H-leucine | R | Cat | (Holstege, 1989) |
|  |  |  | BDA | A | Rat | (Jones et al., 2016) |
|  | Hypoglossal nucleus |  | BDA | A | Rat | (Jones et al., 2016) |
|  | Ambiguus nucleus |  | HRP | R | Bat^2^ | (Rübsamen and Schweizer, 1986) |
|  |  |  | ^3^H-leucine | R | Cat | (Holstege, 1989) |
|  |  |  | WGA-HRP | A | Monkey^3^ | (VanderHorst et al., 2001) |
|  |  |  | BDA | A | Rat | (Jones et al., 2016) |
|  | Pre-Bötzinger complex |  | BDA | A | Rat | (Jones et al., 2016) |
|  | Bötzinger complex |  | BDA | A | Rat | (Jones et al., 2016) |
|  | Retrotrapezoid n. |  | CTb | R | Rat | (Rosin et al., 2006) |
|  |  |  | BDA | A | Rat | (Jones et al., 2016) |
|  | NTS |  | BDA | A | Rat | (Jones et al., 2016) |
|  | rVRG |  | Biocytin | R | Rat | (Zheng et al., 1998) |
|  |  |  | BDA | A | Rat | (Jones et al., 2016) |
|  | Kölliker-Fuse nucleus |  | ^3^H-leucine | R | Cat | (Holstege, 1989) |
|  |  |  | BDA | A | Rat | (Jones et al., 2016) |
|  | Lateral parabrachial n. |  | ^3^H-leucine | R | Cat | (Holstege, 1989) |
|  | Periaqueductal gray |  | BDA | A | Rat | (Jones et al., 2016) |
| Reticular formation | Phrenic nucleus |  | WGA-HRP | R | Rabbit | (Ellenberger et al., 1990b) |
|  |  |  | Pseudorab | R | Rat | (Dobbins and Feldman, 1994) |
|  |  |  | Rabies | R | Cat | (Lois et al., 2009) |
|  | Facial nucleus | Glu  Gly  GABA  5-HT | FG | R | Rat | (Li et al., 1993) |
|  |  |  | BDA  CTb  WGA-HRP | R | Rat | (Dauvergne et al., 2001) |
|  |  |  | Nbiotin | R | Rat | (Moore et al., 2013) |
|  |  |  | AAV | A | Mouse | (Takatoh et al., 2022) |
|  | Hypoglossal nucleus |  | BDA  CTb  WGA-HRP | R | Rat | (Dauvergne et al., 2001) |
|  |  |  | Pseudorab | R | Rat | (Chamberlin et al., 2007) |
|  |  |  | Rabies | R | Mouse | (Guo et al., 2020) |
|  | Ambiguus nucleus |  | HRP | R | Bat^2^ | (Rübsamen and Schweizer, 1986) |
|  | Pre-Bötzinger complex |  | Rabies | R | Mouse | (Yang et al., 2020) |
|  | Lateral parafacial n. | Gly  GABA  Glu | HSV | R | Rat | (Biancardi et al., 2021) |
|  | Retrotrapezoid n. |  | CTb | R | Rat | (Rosin et al., 2006) |
|  | Locus coeruleus |  | Rabies | R | Mouse | (Schwarz et al., 2015) |
|  | Dorsal raphe |  | WGA-HRP | R | Rat | (Lee et al., 2003) |
|  |  |  | Rabies | R | Rat | (Ogawa et al., 2014) |
|  |  |  | Rabies | R | Mouse | (Pollak Dorocic et al., 2014) |
|  |  |  | CTb | R | Rat | (Peyron et al., 2018) |
|  | Caudal raphe |  | CTb | R | Rat | (Hermann et al., 1997) |
|  |  |  | Rabies | R | Rat | (Ogawa et al., 2014) |
|  | Cervical spinal cored |  | True blue | R | Rat | (Jones and Yang, 1985) |
|  | Bed n. stria term. |  | FG | R | Tree shrew^1^ | (Ni et al., 2016) |
|  | Cerebellar cortex | Glu | PHA-L | A | Rat | (Ruigrok et al., 1995) |
|  |  |  | CTb | R | Mouse | (Yuengert et al., 2015) |
|  | Fastigial nucleus | Glu | HRP | R | Cat | (Ruggiero et al., 1977) |
|  |  |  | PHA-L | A | Rat | (Ruigrok et al., 1995) |
|  | Interposed nucleus | Glu | PHA-L | A | Rat | (Ruigrok et al., 1995) |
|  | Dentate nucleus | Glu | PHA-L | A | Rat | (Ruigrok et al., 1995) |
|  | Basal pons |  | WGA-HRP | R | Rat | (Mihailoff et al., 1989) |
|  | Inferior olive |  | HRP | R | Rat | (Swenson and Castro, 1983) |
|  | Vestibular nuclei |  | Rabies | R | Mouse | (Shi et al., 2021) |
|  | Lateral parabrachial n. |  | Microbead | R | Cat | (Quattrochi et al., 1998) |
|  | Medial parabrachial n. |  | FB | R | Mouse | (Hashimoto et al., 2018) |
|  | Periaqueductal gray | Glu | WGA-HRP | R | Rat | (Beitz, 1989) |
|  |  |  | FB / FG | R | Rat | (Herbert and Saper, 1992) |
|  | PPTg |  | WGA-HRP | R | Rat | (Semba and Fibiger, 1992) |
|  |  |  | WGA-HRP | R | Rat | (Steininger et al., 1992) |
| Cervical spinal cord | Phrenic nucleus |  | Pseudorab | R | Rat | (Lane et al., 2008) |
|  | Reticular formation | Glu | Lenti | A | Mouse | (Pop et al., 2022) |
|  |  |  | FG | R | Mouse | (Pop et al., 2022) |
|  | Inferior olive | Glu | HRP | R | Rat | (Swenson and Castro, 1983) |
|  |  |  | FG | R | Mouse | (Pop et al., 2022) |
|  | Vestibular nuclei |  | PHA-L  CTb  BDA | A | Rat | (Matsushita et al., 1995) |
|  |  |  | CTb | R | Ferret | (Jian et al., 2005) |
|  | Periaqueductal gray |  | PHA-L | R | Rat | (Keay et al., 1997) |
| Central amygdala | Hypoglossal nucleus | GABA | Rabies | R | Mouse | (Guo et al., 2020) |
|  | Pre-Bötzinger complex | GABA | Rabies | R | Mouse | (Yang et al., 2020) |
|  |  |  | CTb | R | Rat | (Trevizan-Baú et al., 2021a) |
|  | Retrotrapezoid n. | GABA | CTb | R | Rat | (Rosin et al., 2006) |
|  | NTS | GABA | CTb | R | Rat | (Rinaman, 1998) |
|  |  |  | CTb | R | Mouse | (Gasparini et al., 2020) |
|  | Locus coeruleus | GABA | Rabies | R | Mouse | (Schwarz et al., 2015) |
|  |  |  | AAV | A | Mouse | (Liu et al., 2021a) |
|  | Dorsal raphe | GABA | CTb | R | Rat | (Peyron et al., 1998) |
|  |  |  | Rabies | R | Rat | (Ogawa et al., 2014) |
|  |  |  | Rabies | R | Mouse | (Pollak Dorocic et al., 2014) |
|  |  |  | Rabies | R | Mouse | (Weissbourd et al., 2014) |
|  | Caudal raphe | GABA | CTb | R | Rat | (Hermann et al., 1997) |
|  | Bed n. stria term. | GABA | CTb | R | Rat | (Shin et al., 2008) |
|  |  |  | FG | R | Tree shrew^1^ | (Ni et al., 2016) |
|  |  |  | AAV | A | Mouse | (Liu et al., 2021a) |
|  | Lateral hypothalamus | GABA | CTb | R | Rat | (Yoshida et al., 2006) |
|  | Basal pons |  | WGA-HRP | R | Rat | (Mihailoff et al., 1989) |
|  | Lateral parabrachial n. | GABA | PHA-L | A | Rat | (Moga et al., 1990) |
|  |  |  | WGA-HRP | R | Rat | (Moga et al., 1990) |
|  |  |  | AAV | A | Mouse | (Yang et al., 2021) |
|  | Medial parabrachial n. | GABA | PHA-L | A | Rat | (Moga et al., 1990) |
|  |  |  | WGA-HRP | R | Rat | (Moga et al., 1990) |
|  |  |  | AAV | A | Mouse | (Liu et al., 2021a) |
|  |  |  | AAV | A | Mouse | (Yang et al., 2021) |
|  | Periaqueductal gray | GABA | WGA-HRP | A | Rat | (Rizvi et al., 1991) |
|  |  |  | BDA | A | Rat | (Oka et al., 2008) |
|  |  |  | CTb | R | Rat | (Oka et al., 2008) |
|  |  |  | CTb | R | Mouse | (Frontera et al., 2020) |
|  |  |  | AAV | A | Mouse | (Liu et al., 2021a) |
|  | PPTg | GABA | WGA-HRP | R | Rat | (Semba and Fibiger, 1992) |
| Bed n. stria terminalis | NTS |  | CTb | R | Mouse | (Gasparini et al., 2020) |
|  | Locus coeruleus |  | CTb | R | Rat | (Luppi et al., 1995) |
|  |  |  | Rabies | R | Mouse | (Schwarz et al., 2015) |
|  | Dorsal raphe | GABA | CTb | R | Rat | (Peyron et al., 1998) |
|  |  |  | WGA-HRP | R | Rat | (Lee et al., 2003) |
|  |  |  | Rabies | R | Rat | (Ogawa et al., 2014) |
|  |  |  | Rabies | R | Mouse | (Pollak Dorocic et al., 2014) |
|  |  |  | Rabies | R | Mouse | (Weissbourd et al., 2014) |
|  | Caudal raphe |  | CTb | R | Rat | (Hermann et al., 1997) |
|  | Central amygdala |  | PHA-L | A | Rat | (Dong et al., 2001) |
|  |  |  | Rabies | R | Mouse | (Fu et al., 2020) |
|  | Lateral hypothalamus |  | PI/TB/EB | R | Rat | (Woolf and Butcher, 1986) |
|  |  |  | CTb | R | Rat | (Yoshida et al., 2006) |
|  |  |  | PHA-L | A | Mouse | (Barbier et al., 2021) |
|  | Paraventricular n. |  | PHA-L | A | Mouse | (Barbier et al., 2021) |
|  |  |  | Rabies | R | Mouse | (Singh et al., 2022) |
|  | Dorsomedial hypothal. |  | FG | R | Rat | (Thompson and Swanson, 1998) |
|  |  |  | PHA-L | A | Mouse | (Barbier et al., 2021) |
|  | Lateral parabrachial n. |  | PHA-L | A | Rat | (Moga et al., 1990) |
|  | Medial parabrachial n. | Glu  GABA | PHA-L | A | Rat | (Moga et al., 1990) |
|  |  |  | AAV | A | Mouse | (Luskin et al., 2021) |
|  | PPTg |  | WGA-HRP | R | Rat | (Steininger et al., 1992) |
| Lateral hypothalamus | Phrenic nucleus | Orexin | CTb | R | Rat | (Young et al., 2005) |
|  | Hypoglossal nucleus | Orexin | CTb | R | Rat | (Fung et al., 2001) |
|  |  |  | Rabies | R | Mouse | (Guo et al., 2020) |
|  | Pre-Bötzinger complex | Orexin | CTb | R | Rat | (Young et al., 2005) |
|  |  |  | Rabies | R | Mouse | (Yang et al., 2020) |
|  |  |  | CTb | R | Rat | (Trevizan-Baú et al., 2021a) |
|  | Retrotrapezoid n. | Orexin | CTb | R | Rat | (Rosin et al., 2006) |
|  | NTS | Orexin | CTb | R | Rat | (Rinaman, 1998) |
|  |  |  | CTb | R | Mouse | (Gasparini et al., 2020) |
|  | Locus coeruleus | Orexin | CTb | R | Rat | (Luppi et al., 1995) |
|  |  |  | Rabies | R | Mouse | (Schwarz et al., 2015) |
|  |  |  | CTb | R | Rat | (Arima et al., 2019) |
|  | Dorsal raphe | Glu  GABA  Orexin | CTb | R | Rat | (Peyron et al., 1998) |
|  |  |  | PHA-L | A | Rat | (Peyron et al., 1998) |
|  |  |  | WGA-HRP | R | Rat | (Lee et al., 2003) |
|  |  |  | WGA-HRP | R | Rat | (Lee et al., 2005) |
|  |  |  | Rabies | R | Rat | (Ogawa et al., 2014) |
|  |  |  | Rabies | R | Mouse | (Pollak Dorocic et al., 2014) |
|  |  |  | Rabies | R | Mouse | (Weissbourd et al., 2014) |
|  |  |  | CTb | R | Rat | (Arima et al., 2019) |
|  | Caudal raphe | Orexin | CTb | R | Rat | (Hermann et al., 1997) |
|  |  |  | Rabies | R | Rat | (Ogawa et al., 2014) |
|  | Central amygdala |  | Rabies | R | Rat | (Fu et al., 2020) |
|  | Bed n. of stria term. |  | CTb | R | Rat | (Shin et al., 2008) |
|  |  |  | FG | R | Tree shrew^1^ | (Ni et al., 2016) |
|  | Paraventricular n. |  | Rabies | R | Mouse | (Singh et al., 2022) |
|  | Dorsomedial hypothal. |  | FG | R | Rat | (Thompson and Swanson, 1998) |
|  | Cerebellar cortex | Orexin | WGA-HRP | R | Cat | (Dietrichs, 1984) |
|  |  |  | FG  BDA | R | Rat | (Çavdar et al., 2018) |
|  | Fastigial nucleus | Orexin | FG | R | Rat | (Ciriello and Caverson, 2014) |
|  | Interposed nucleus | Orexin | FG | R | Rat | (Ciriello and Caverson, 2014) |
|  | Basal pons |  | WGA-HRP | R | Rat | (Mihailoff et al., 1989) |
|  |  |  | BDA | A | Rat | (Liu and Mihailoff, 1999) |
|  | Kölliker-Fuse nucleus | Orexin | CTb | R | Rat | (Yokota et al., 2016) |
|  |  |  | CTb | R | Rat | (Trevizan-Baú et al., 2021a) |
|  | Periaqueductal gray | Orexin | CTb | R | Rat | (Trevizan-Baú et al., 2021a) |
|  | PPTg |  | WGA-HRP | A | Rat | (Semba and Fibiger, 1992) |
|  |  |  | WGA-HRP | R | Rat | (Semba and Fibiger, 1992) |
|  |  |  | WGA-HRP | R | Rat | (Steininger et al., 1992) |
| Paraventricular n. | Phrenic nucleus | OXT  VP | CTb | R | Rat | (Kc et al., 2002) |
|  |  |  | CTb | R | Rat | (Mack et al., 2002) |
|  | Facial nucleus |  | PHA-L | A | Rat | (Geerling et al., 2010) |
|  | Hypoglossal nucleus | OXT  VP | Pseudorab | R | Rat | (Mack et al., 2007) |
|  |  |  | Rabies | R | Mouse | (Guo et al., 2020) |
|  | Nucleus ambiguus |  | PHA-L | A | Rat | (Geerling et al., 2010) |
|  | Pre-Bötzinger complex | OXT | CTb | R | Rat | (Mack et al., 2002) |
|  |  |  | Rabies | R | Mouse | (Yang et al., 2020) |
|  |  |  | CTb | R | Rat | (Trevizan-Baú et al., 2021a) |
|  | PiCo |  | FG | R | Rat | (Oliveira et al., 2021) |
|  | Retrotrapezoid n. |  | CTb | R | Rat | (Rosin et al., 2006) |
|  |  |  | PHA-L | A | Rat | (Geerling et al., 2010) |
|  | NTS | CRH  OXT | CTb | R | Rat | (Rinaman, 1998) |
|  |  |  | PHA-L | A | Rat | (Geerling et al., 2010) |
|  |  |  | CTb | R | Rat | (Ruyle et al., 2018) |
|  |  |  | AAV | A | Rat | (Ruyle et al., 2019) |
|  |  |  | CTb | R | Mouse | (Gasparini et al., 2020) |
|  |  |  | AAV | A | Mouse | (Singh et al., 2022) |
|  | Locus coeruleus |  | CTb | R | Rat | (Luppi et al., 1995) |
|  |  |  | PHA-L | A | Rat | (Zheng et al., 1995) |
|  |  |  | Rabies | R | Mouse | (Schwarz et al., 2015) |
|  | Dorsal raphe | Glu  OXT  VP | PHA-L | A | Rat | (Zheng et al., 1995) |
|  |  |  | CTb | R | Rat | (Peyron et al., 1998) |
|  |  |  | WGA-HRP | R | Rat | (Lee et al., 2003) |
|  |  |  | PHA-L | A | Rat | (Geerling et al., 2010) |
|  |  |  | Rabies | R | Mouse | (Pollak Dorocic et al., 2014) |
|  |  |  | Rabies | R | Mouse | (Weissbourd et al., 2014) |
|  | Caudal raphe |  | PHA-L | A | Rat | (Zheng et al., 1995) |
|  |  |  | CTb | R | Rat | (Hermann et al., 1997) |
|  |  |  | Rabies | R | Rat | (Ogawa et al., 2014) |
|  | Paratrigeminal nucleus |  | PHA-L | A | Rat | (Geerling et al., 2010) |
|  | Spinal trigeminal n. |  | PHA-L | A | Rat | (Zheng et al., 1995) |
|  |  |  | PHA-L | A | Rat | (Geerling et al., 2010) |
|  | Central amygdala |  | Rabies | R | Rat | (Fu et al., 2020) |
|  | Bed n. stria term. |  | AAV | A | Mouse | (Singh et al., 2022) |
|  | Lateral hypothalamus |  | CTb | R | Rat | (Yoshida et al., 2006) |
|  |  |  | AAV | A | Mouse | (Singh et al., 2022) |
|  | Dorsomedial hypothal. |  | FG | R | Rat | (Thompson and Swanson, 1998) |
|  |  |  | AAV | A | Mouse | (Singh et al., 2022) |
|  | Cerebellar cortex |  | WGA-HRP | R | Cat | (Dietrichs, 1984) |
|  |  |  | FG  BDA | R | Rat | (Çavdar et al., 2018) |
|  | Kölliker-Fuse nucleus |  | CTb | R | Rat | (Yokota et al., 2016) |
|  |  |  | CTb | R | Rat | (Trevizan-Baú et al., 2021a) |
|  | Lateral parabrachial n. |  | PHA-L | A | Rat | (Zheng et al., 1995) |
|  |  |  | PHA-L | A | Rat | (Geerling et al., 2010) |
|  |  |  | AAV | A | Mouse | (Singh et al., 2022) |
|  | Medial parabrachial n. |  | PHA-L | A | Rat | (Zheng et al., 1995) |
|  |  |  | PHA-L | A | Rat | (Geerling et al., 2010) |
|  |  |  | AAV | A | Mouse | (Singh et al., 2022) |
|  | Periaqueductal gray |  | PHA-L | A | Rat | (Zheng et al., 1995) |
|  |  |  | PHA-L | A | Rat | (Geerling et al., 2010) |
|  |  |  | CTb | R | Rat | (Trevizan-Baú et al., 2021a) |
|  |  |  | AAV | A | Mouse | (Singh et al., 2022) |
|  | PPTg |  | PHA-L | A | Rat | (Zheng et al., 1995) |
|  |  |  | PHA-L | A | Rat | (Geerling et al., 2010) |
| Dorsomedial hypothal. | NTS |  | PHA-L | A | Rat | (Thompson et al., 1996) |
|  |  |  | CTb | R | Rat | (Rinaman, 1998) |
|  | Dorsal raphe |  | CTb | R | Rat | (Peyron et al., 2018) |
|  | Caudal raphe |  | CTb | R | Rat | (Trevizan-Baú et al., 2021a) |
|  | Bed n. stria term. |  | PHA-L | A | Rat | (Thompson et al., 1996) |
|  |  |  | CTb | R | Rat | (Shin et al., 2008) |
|  | Lateral hypothalamus |  | CTb | R | Rat | (Yoshida et al., 2006) |
|  | Paraventricular n. |  | PHA-L | A | Rat | (Thompson et al., 1996) |
|  |  |  | Rabies | R | Mouse | (Singh et al., 2022) |
|  | Cerebellar cortex |  | FG  BDA | R | Rat | (Çavdar et al., 2018) |
|  | Kölliker-Fuse nucleus |  | CTb | R | Rat | (Trevizan-Baú et al., 2021a) |
|  | Periaqueductal gray | Glu | WGA-HRP | R | Rat | (Beitz, 1989) |
|  |  |  | PHA-L | A | Rat | (Thompson et al., 1996) |
|  |  |  | CTb | R | Rat | (Trevizan-Baú et al., 2021a) |
| Cerebellar cortex | Locus coeruleus | GABA | Rabies | R | Mouse | (Schwarz et al., 2015) |
|  | Cerebellar nuclei | GABA | L7 | A | Rat | (De Zeeuw and Berrebi, 1995) |
|  |  |  | BDA | A | Rabbit | (Sadakane et al., 2000) |
|  |  |  | BDA | A | Rat | (Sugihara et al., 2009) |
|  |  |  | Optogen | A | Mouse | (Chaumont et al., 2013) |
|  |  |  | Optogen | A | Mouse | (Witter et al., 2013) |
|  |  |  | Optogen | A | Mouse | (Lindeman et al., 2021) |
|  |  |  | AAV | A | Mouse | (Hashimoto et al., 2018) |
|  |  |  | AAV | A | Mouse | (Judd et al., 2021) |
|  | Vestibular nuclei | GABA | L7 | A | Rat | (De Zeeuw and Berrebi, 1995) |
|  |  |  | BDA | A | Rabbit | (Sadakane et al., 2000) |
|  |  |  | Rabies | R | Mouse | (Shi et al., 2021) |
|  | Lateral parabrachial n. | GABA | HRP | R | Rabbit | (Sadakane et al., 2000) |
|  | Medial parabrachial n. | GABA | BDA | A | Rat | (Sugihara et al., 2009) |
|  |  |  | AAV | A | Mouse | (Hashimoto et al., 2018) |
|  |  |  | FB | R | Mouse | (Hashimoto et al., 2018) |
| Fastigial nucleus | Facial nucleus | Glu | AAV | A | Mouse | (Fujita et al., 2020) |
|  | Hypoglossal nucleus | Glu | Rabies | R | Mouse | (Guo et al., 2020) |
|  | Reticular formation | Glu  Gly | ^3^H aa | A | Monkey^3^ | (Batton et al., 1977) |
|  |  |  | PHA-L  BDA | A | Rat | (Teune et al., 2000) |
|  |  |  | AAV | A | Mouse | (Fujita et al., 2020) |
|  |  |  | Texas Red | R | Mouse | (Bagnall et al., 2009) |
|  | Cerebellar cortex | GABA  Gly | HRP | R | Cat | (Gould and Graybiel, 1976) |
|  |  |  | AAV | A | Mouse | (Fujita et al., 2020) |
|  | Inferior olive  *Medial accessory olive* | GABA | ^3^H aa | A | Monkey^3^ | (Batton et al., 1977) |
|  |  |  | PHA-L  BDA | A | Rat | (Teune et al., 2000) |
|  |  |  | AAV | A | Mouse | (Fujita et al., 2020) |
|  | MDJ | Glu | PHA-L  BDA | A | Rat | (Teune et al., 2000) |
|  |  |  | CTb | R | Mouse | (Wang et al., 2021) |
|  | Vestibular nuclei | Glu  Gly | ^3^H aa | A | Monkey^3^ | (Batton et al., 1977) |
|  |  |  | PHA-L  BDA | A | Rat | (Teune et al., 2000) |
|  |  |  | Texas Red | R | Mouse | (Bagnall et al., 2009) |
|  |  |  | AAV | A | Mouse | (Fujita et al., 2020) |
|  |  |  | Rabies | R | Mouse | (Shi et al., 2021) |
|  | Kölliker-Fuse nucleus | Glu | AAV | A | Mouse | (Fujita et al., 2020) |
|  | Lateral parabrachial n. | Glu | PHA-L | A | Rat | (Teune et al., 2000) |
|  | Medial parabrachial n. | Glu | PHA-L  BDA | A | Rat | (Teune et al., 2000) |
|  | Periaqueductal gray | Glu | PHA-L  BDA | A | Rat | (Teune et al., 2000) |
|  |  |  | AAV | A | Mouse | (Frontera et al., 2020) |
|  |  |  | CTb | R | Mouse | (Frontera et al., 2020) |
|  |  |  | AAV | A | Mouse | (Fujita et al., 2020) |
| Interposed nucleus | Trigeminal motor n. | Glu | AAV | A | Mouse | (Judd et al., 2021) |
|  | Spinal trigeminal n. | Glu  GABA | PHA-L  BDA | A | Rat | (Teune et al., 2000) |
|  |  |  | AAV | A | Mouse | (Judd et al., 2021) |
|  | Reticular formation | Glu | PHA-L  BDA | A | Rat | (Teune et al., 2000) |
|  |  |  | AAV | A | Mouse | (Judd et al., 2021) |
|  | Lateral hypothalamus | Glu | DTR | A | Rat | (Lu et al., 2015) |
|  |  |  | FR | R | Rat | (Teune et al., 2000) |
|  | Cerebellar cortex | GABA  Gly  Glu | HRP | R | Cat | (Gould and Graybiel, 1976) |
|  |  |  | HRP | R | Monkey^3^ | (Tolbert et al., 1977) |
|  |  |  | Ctb | R | Mouse | (Gao et al., 2016) |
|  |  |  | AAV | A | Mouse | (Judd et al., 2021) |
|  | Basal pons | Glu  GABA | AAV | A | Mouse | (Judd et al., 2021) |
|  | Inferior olive  *Dorsal accessory olive*  *Medial accessory olive* | GABA | PHA-L  BDA | A | Rat | (Teune et al., 2000) |
|  | MDJ | Glu | CTb | R | Mouse | (Wang et al., 2021) |
|  | Vestibular nuclei | Glu | PHA-L  BDA | A | Rat | (Teune et al., 2000) |
|  |  |  | AAV | A | Mouse | (Judd et al., 2021) |
|  |  |  | Rabies | R | Mouse | (Shi et al., 2021) |
|  | Medial parabrachial n. | Glu | AAV | A | Mouse | (Judd et al., 2021) |
|  | Periaqueductal gray | Glu | AAV | A | Mouse | (Judd et al., 2021) |
|  | PPTg | Glu | WGA-HRP | R | Rat | (Steininger et al., 1992) |
| Dentate nucleus | Spinal trigeminal n. | Glu | PHA-L  BDA | A | Rat | (Teune et al., 2000) |
|  | Reticular formation | Glu | PHA-L  BDA | A | Rat | (Teune et al., 2000) |
|  | Lateral hypothalamus | Glu | PHA-L  BDA | A | Rat | (Teune et al., 2000) |
|  | Cerebellar cortex | GABA  Gly | HRP | R | Cat | (Gould and Graybiel, 1976) |
|  |  |  | HRP | R | Monkey^3^ | (Tolbert et al., 1977) |
|  |  |  | E-phys |  | Mouse | (Uusisaari and Knöpfel, 2010) |
|  | Basal pons | Glu | WGA-HRP | R | Rat | (Mihailoff et al., 1989) |
|  |  |  | PHA-L  BDA | A | Rat | (Teune et al., 2000) |
|  | Inferior olive  *Principal olive* | GABA | PHA-L  BDA | A | Rat | (Teune et al., 2000) |
|  | MDJ | Glu | PHA-L  BDA | A | Rat | (Teune et al., 2000) |
|  |  |  | CTb | R | Mouse | (Wang et al., 2021) |
|  | Periaqueductal gray | Glu | PHA-L  BDA | A | Rat | (Teune et al., 2000) |
|  | PPTg | Glu | WGA-HRP | R | Rat | (Steininger et al., 1992) |
| Cerebellar nuclei  *Not specified* | Dorsal raphe | Glu | Rabies | R | Mouse | (Weissbourd et al., 2014) |
|  | Reticular formation | Glu | Rabies | R | Mouse | (Takatoh et al., 2022) |
| Basal pons | Cerebellar cortex | Glu | CTb | R | Rat | (Pijpers and Ruigrok, 2006) |
|  |  |  | HRP | R | Mouse | (Fu et al., 2011) |
|  |  |  | AAV | A | Mouse | (Huang et al., 2013) |
|  |  |  | AAV | A | Mouse | (Henschke and Pakan, 2020) |
|  | Fastigial nucleus | Glu | HRP | R | Cat | (Ruggiero et al., 1977) |
|  |  |  | PHA-L | A | Rat | (Mihailoff, 1993) |
|  | Interposed nucleus | Glu | PHA-L | A | Rat | (Mihailoff, 1993) |
|  |  |  | Rabies | R | Mouse | (Judd et al., 2021) |
|  | Dentate nucleus | Glu | E-phys |  | Cat | (Shinoda et al., 1992) |
|  |  |  | PHA-L | A | Rat | (Mihailoff, 1993) |
| Inferior olive | Cerebellar cortex | Glu | Degen. | A | Cat | (Szentágothai and Rajkovits, 1959) |
|  |  |  | BDA | A | Rat | (Sugihara et al., 2001) |
|  |  |  | HRP | R | Mouse | (Fu et al., 2011) |
|  | Fastigial nucleus | Glu | HRP | R | Cat | (Ruggiero et al., 1977) |
|  |  |  | HRP | R | Rat | (Swenson and Castro, 1983) |
|  |  |  | BDA | A | Rat | (Sugihara and Shinoda, 2007) |
|  | Interposed nucleus | Glu | HRP | R | Rat | (Swenson and Castro, 1983) |
|  |  |  | BDA | A | Rat | (Pijpers et al., 2005) |
|  |  |  | BDA | A | Rat | (Sugihara and Shinoda, 2007) |
|  | Dentate nucleus | Glu | HRP | R | Rat | (Swenson and Castro, 1983) |
|  |  |  | BDA | A | Rat | (Sugihara and Shinoda, 2007) |
| MDJ | Basal pons |  | WGA-HRP | R | Rat | (Mihailoff et al., 1989) |
|  | Inferior olive |  | HRP | R | Cat | (Onodera, 1984) |
|  |  |  | CTb | R | Mouse | (Wang et al., 2021) |
|  | Vestibular nuclei |  | Rabies | R | Mouse | (Shi et al., 2021) |
| Vestibular nuclei | Facial nucleus | Glu | AAV | A | Mouse | (Shi et al., 2021) |
|  | Hypoglossal nucleus | Glu | Rabies | R | Mouse | (Guo et al., 2020) |
|  | Lateral parafacial n. | Glu  GABA  Gly | HSV | R | Rat | (Biancardi et al., 2021) |
|  | NTS | Glu | PHA-L | A | Rabbit | (Balaban and Beryozkin, 1994) |
|  |  |  | PHA-L | A | Cat | (Yates et al., 1994) |
|  |  |  | Nbiotin | A | Rat | (Bácskai et al., 2002) |
|  |  |  | AAV | A | Mouse | (Shi et al., 2021) |
|  | Locus coeruleus | Glu | Rabies | R | Mouse | (Schwarz et al., 2015) |
|  |  |  | AAV | A | Mouse | (Shi et al., 2021) |
|  | Dorsal raphe | Glu | Rabies | R | Rat | (Ogawa et al., 2014) |
|  |  |  | AAV | A | Mouse | (Shi et al., 2021) |
|  | Caudal raphe |  | Rabies | R | Rat | (Ogawa et al., 2014) |
|  | Spinal trigeminal n. | Glu | BDA | R | Rat | (Buisseret-Delmas et al., 1999) |
|  |  |  | Nbiotin | A | Rat | (Bácskai et al., 2002) |
|  | Reticular formation | Glu | Nbiotin | A | Rat | (Bácskai et al., 2002) |
|  |  |  | AAV | A | Mouse | (Shi et al., 2021) |
|  | Cervical spinal cord |  | PHA-L | A | Cat | (Rose et al., 1992) |
|  |  |  | Nbiotin | A | Rat | (Bácskai et al., 2002) |
|  | Cerebellar cortex | Glu  ACh | HRP | R | Rabbit | (Barmack et al., 1992) |
|  |  |  | HRP | R | Mouse | (Fu et al., 2011) |
|  |  |  | AAV | A | Mouse | (Shi et al., 2021) |
|  | Fastigial nucleus | Glu | AAV | A | Mouse | (Shi et al., 2021) |
|  | Interposed nucleus | Glu | AAV | A | Mouse | (Shi et al., 2021) |
|  | Dentate nucleus | Glu | AAV | A | Mouse | (Shi et al., 2021) |
|  | Inferior olive | Glu | ^3^H Leu | A | Cat | (Gerrits et al., 1985) |
|  |  |  | Nbiotin | A | Rat | (Bácskai et al., 2002) |
|  |  |  | AAV | A | Mouse | (Shi et al., 2021) |
|  | MDJ | Glu | Nbiotin | A | Rat | (Bácskai et al., 2002) |
|  |  |  | AAV | A | Mouse | (Shi et al., 2021) |
|  | Kölliker-Fuse nucleus | Glu | AAV | A | Mouse | (Shi et al., 2021) |
|  | Periaqueductal gray |  | Nbiotin | A | Rat | (Bácskai et al., 2002) |
| Kölliker-Fuse nucleus | Phrenic nucleus | Glu | HRP | R | Cat | (Rikard-Bell et al., 1984) |
|  |  |  | WGA-HRP | R | Rabbit | (Ellenberger et al., 1990b) |
|  |  |  | BDA | A | Rat | (Yokota et al., 2004) |
|  |  |  | FG | R | Rat | (Yokota et al., 2007) |
|  |  |  | BDA | A | Rat | (Song et al., 2012) |
|  |  |  | CTb | R | Mouse | (Yokota et al., 2015) |
|  |  |  | FG | R | Rat | (Yokota et al., 2016) |
|  |  |  | AAV | A | Mouse | (Geerling et al., 2017) |
|  | Facial nucleus | Glu | BDA | A | Rat | (Song et al., 2012) |
|  |  |  | AAV | A | Mouse | (Geerling et al., 2017) |
|  | Hypoglossal nucleus | Glu | BDA | A | Rat | (Song et al., 2012) |
|  |  |  | FG | R | Rat | (Yokota et al., 2007) |
|  |  |  | CTb | R | Mouse | (Yokota et al., 2015) |
|  |  |  | FG | R | Rat | (Yokota et al., 2016) |
|  |  |  | AAV | A | Mouse | (Geerling et al., 2017) |
|  | Ambiguus nucleus | Glu | DY | R | Rat | (Núñez-Abades et al., 1990) |
|  |  |  | BDA | A | Rat | (Song et al., 2012) |
|  | Pre-Bötzinger complex | Glu | Rabies | R | Mouse | (Yang et al., 2020) |
|  | PiCo | Glu | FG | R | Rat | (Oliveira et al., 2021) |
|  | Lateral parafacial n. | Glu | HSV | R | Rat | (Biancardi et al., 2021) |
|  | Retrotrapezoid n. | Glu | CTb-HRP | R | Rabbit | (Li and Song, 2001) |
|  |  |  | BDA | A | Rat | (Song et al., 2012) |
|  |  |  | FG | R | Rat | (Lima et al., 2019) |
|  | NTS | Glu | WGA-HRP | R | Rat | (Fulwiler and Saper, 1984) |
|  |  |  | BDA | A | Rat | (Song et al., 2012) |
|  |  |  | AAV | A | Mouse | (Geerling et al., 2017) |
|  | Dorsal raphe |  | CTb | R | Rat | (Peyron et al., 2018) |
|  | Caudal raphe |  | CTb | R | Rat | (Hermann et al., 1997) |
|  | Spinal trigeminal n. | GABA | BDA | A | Rat | (Song et al., 2012) |
|  |  |  | AAV | A | Mouse | (Geerling et al., 2017) |
|  |  |  | AAV | A | Rat | (Elbaz et al., 2022) |
|  | rVRG | Glu | Biocytin | R | Rat | (Zheng et al., 1998) |
|  |  |  | Beads | R | Rat | (Ellenberger and Feldman, 1990) |
|  |  |  | FG | R | Rat | (Yokota et al., 2016) |
|  | cVRG | Glu | WGA-HRP | R | Cat | (Gerrits and Holstege, 1996) |
|  |  |  | BDA | A | Rat | (Song et al., 2012) |
|  | Reticular formation | GABA | AAV | A | Mouse | (Geerling et al., 2017) |
|  | Dorsomedial hypothal. | GABA | AAV | A | Mouse | (Geerling et al., 2017) |
|  | Cerebellar cortex | Glu | HRP | R | Mouse | (Fu et al., 2011) |
|  | Vestibular nuclei |  | Rabies | R | Mouse | (Shi et al., 2021) |
|  | Lateral parabrachial n. | Glu | BDA | A | Rat | (Song et al., 2012) |
|  |  |  | AAV | A | Mouse | (Geerling et al., 2017) |
|  | Medial parabrachial n. | Glu | BDA | A | Rat | (Song et al., 2012) |
|  |  |  | AAV | A | Mouse | (Geerling et al., 2017) |
|  | Periaqueductal gray | Glu | CTB | R | Rat | (Trevizan-Baú et al., 2021b) |
| Lateral parabrachial n. | Hypoglossal nucleus | Glu | CTb | R | Mouse | (Yokota et al., 2015) |
|  | Ambiguus nucleus |  | HRP | R | Bat^2^ | (Rübsamen and Schweizer, 1986) |
|  | Pre-Bötzinger complex |  | Rabies | R | Mouse | (Yang et al., 2020) |
|  |  |  | AAV | R | Mouse | (Yu et al., 2021) |
|  | Bötzinger complex |  | WGA-HRP | R | Cat | (Gang et al., 1995) |
|  | Lateral parafacial n. | Glu | HSV | R | Rat | (Biancardi et al., 2021) |
|  | Retrotrapezoid n. |  | CTb | R | Rat | (Rosin et al., 2006) |
|  | NTS |  | ^3^H aa | A | Rat | (Saper and Loewy, 1980) |
|  |  |  | WGA-HRP | R | Rat | (Herbert et al., 1990) |
|  |  |  | Biocytin | A | Rat | (Bianchi et al., 1998) |
|  | Locus coeruleus |  | CTb | R | Rat | (Luppi et al., 1995) |
|  | Dorsal raphe | Glu | ^3^H aa | A | Rat | (Saper and Loewy, 1980) |
|  |  |  | Biocytin | A | Rat | (Bianchi et al., 1998) |
|  |  |  | WGA-HRP | R | Rat | (Lee et al., 2003) |
|  |  |  | CTb | R | Rat | (Peyron et al., 2018) |
|  | Caudal raphe |  | ^3^H aa | A | Rat | (Saper and Loewy, 1980) |
|  |  |  | CTb | R | Rat | (Hermann et al., 1997) |
|  |  |  | Biocytin | A | Rat | (Bianchi et al., 1998) |
|  | cVRG |  | WGA-HRP | R | Cat | (Gerrits and Holstege, 1996) |
|  | Reticular formation |  | Biocytin | A | Rat | (Bianchi et al., 1998) |
|  | Central amygdala |  | ^3^H aa | A | Rat | (Saper and Loewy, 1980) |
|  |  |  | WGA-HRP | R | Rat | (Moga et al., 1990) |
|  |  |  | Biocytin | A | Rat | (Bianchi et al., 1998) |
|  |  |  | Microbead | R | Mouse | (Tokita et al., 2010) |
|  |  |  | CTb | R | Rat | (Bienkowski and Rinaman, 2013) |
|  | Bed n. stria term. |  | ^3^H aa | A | Rat | (Saper and Loewy, 1980) |
|  |  |  | WGA-HRP | R | Rat | (Fulwiler and Saper, 1984) |
|  |  |  | WGA-HRP | R | Rat | (Moga et al., 1990) |
|  |  |  | Biocytin | A | Rat | (Bianchi et al., 1998) |
|  |  |  | CTb | R | Rat | (Shin et al., 2008) |
|  |  |  | CTb | R | Rat | (Bienkowski and Rinaman, 2013) |
|  |  |  | FG | R | Tree shrew^1^ | (Ni et al., 2016) |
|  | Lateral hypothalamus |  | ^3^H aa | A | Rat | (Saper and Loewy, 1980) |
|  |  |  | WGA-HRP | R | Rat | (Fulwiler and Saper, 1984) |
|  |  |  | WGA-HRP | R | Rat | (Moga et al., 1990) |
|  |  |  | Biocytin | A | Rat | (Bianchi et al., 1998) |
|  |  |  | CTb | R | Rat | (Yoshida et al., 2006) |
|  |  |  | BDA | A | Rat | (Arima et al., 2019) |
|  | Paraventricular n. |  | ^3^H aa | A | Rat | (Saper and Loewy, 1980) |
|  |  |  | WGA-HRP | R | Rat | (Fulwiler and Saper, 1984) |
|  |  |  | WGA-HRP | R | Rat | (Moga et al., 1990) |
|  |  |  | Rabies | R | Mouse | (Singh et al., 2022) |
|  | Dorsomedial hypothal. |  | FG | R | Rat | (Thompson and Swanson, 1998) |
|  | Vestibular nuclei |  | Biocytin | A | Rat | (Bianchi et al., 1998) |
|  | Periaqueductal gray |  | Biocytin | A | Rat | (Bianchi et al., 1998) |
|  | PPTg |  | WGA-HRP | R | Rat | (Steininger et al., 1992) |
| Medial parabrachial n. | Hypoglossal nucleus |  | ^3^H aa | A | Rat | (Saper and Loewy, 1980) |
|  |  |  | Rabies | R | Mouse | (Guo et al., 2020) |
|  | Ambiguus nucleus |  | HRP | R | Bat^2^ | (Rübsamen and Schweizer, 1986) |
|  |  |  | DY | R | Rat | (Núñez-Abades et al., 1990) |
|  | Pre-Bötzinger complex |  | Rabies | R | Mouse | (Yang et al., 2020) |
|  | Bötzinger complex |  | WGA-HRP | R | Cat | (Gang et al., 1995) |
|  | Lateral parafacial n. | Glu  GABA  Gly | HSV | R | Rat | (Biancardi et al., 2021) |
|  | NTS |  | WGA-HRP | R | Rat | (Herbert et al., 1990) |
|  |  |  | Biocytin | A | Rat | (Bianchi et al., 1998) |
|  | Retrotrapezoid n. |  | CTb | R | Rat | (Rosin et al., 2006) |
|  | Locus coeruleus |  | CTb | R | Rat | (Luppi et al., 1995) |
|  | Dorsal raphe | Glu | ^3^H aa | A | Rat | (Saper and Loewy, 1980) |
|  |  |  | Biocytin | A | Rat | (Bianchi et al., 1998) |
|  |  |  | WGA-HRP | R | Rat | (Lee et al., 2003) |
|  |  |  | CTb | R | Rat | (Peyron et al., 2018) |
|  | Caudal raphe |  | ^3^H aa | A | Rat | (Saper and Loewy, 1980) |
|  |  |  | Biocytin | A | Rat | (Bianchi et al., 1998) |
|  | rVRG |  | FG | R | Rat | (Yokota et al., 2016) |
|  | cVRG |  | WGA-HRP | R | Cat | (Gerrits and Holstege, 1996) |
|  | Reticular formation |  | Biocytin | A | Rat | (Bianchi et al., 1998) |
|  | Central amygdala |  | WGA-HRP | R | Rat | (Fulwiler and Saper, 1984) |
|  |  |  | WGA-HRP | R | Rat | (Moga et al., 1990) |
|  |  |  | CTb | R | Rat | (Bienkowski and Rinaman, 2013) |
|  | Bed n. stria termin. |  | Biocytin | A | Rat | (Bianchi et al., 1998) |
|  |  |  | CTb | R | Rat | (Bienkowski and Rinaman, 2013) |
|  |  |  | FG | R | Tree shrew^1^ | (Ni et al., 2016) |
|  | Lateral hypothalamus |  | ^3^H aa | A | Rat | (Saper and Loewy, 1980) |
|  |  |  | WGA-HRP | R | Rat | (Fulwiler and Saper, 1984) |
|  |  |  | WGA-HRP | R | Rat | (Moga et al., 1990) |
|  |  |  | Biocytin | A | Rat | (Bianchi et al., 1998) |
|  | Cerebellar cortex | Glu | HRP | R | Mouse | (Fu et al., 2011) |
|  | Periaqueductal gray |  | Biocytin | A | Rat | (Bianchi et al., 1998) |
| Periaqueductal gray | Ambiguus nucleus |  | HRP | R | Bat^2^ | (Rübsamen and Schweizer, 1986) |
|  | Pre-Bötzinger complex |  | CTb | R | Rat | (Trevizan-Baú et al., 2021b) |
|  | PiCo |  | FG | R | Rat | (Oliveira et al., 2021) |
|  | Lateral parafacial n. | Glu  GABA | HSV | R | Rat | (Biancardi et al., 2021) |
|  | Retrotrapezoid n. |  | CTb | R | Rat | (Rosin et al., 2006) |
|  | NTS | GABA  Glu | WGA-HRP | A | Monkey^3^ | (Vanderhorst et al., 2000) |
|  |  |  | AAV | A | Mouse | (Chen et al., 2022) |
|  |  |  | AAV | R | Mouse | (Chen et al., 2022) |
|  | Locus coeruleus |  | CTb | R | Rat | (Luppi et al., 1995) |
|  |  |  | Rabies | R | Mouse | (Schwarz et al., 2015) |
|  | Dorsal raphe |  | Rabies | R | Rat | (Ogawa et al., 2014) |
|  |  |  | Rabies | R | Mouse | (Pollak Dorocic et al., 2014) |
|  |  |  | CTb | R | Rat | (Peyron et al., 2018) |
|  | Caudal raphe |  | CTb | R | Rat | (Hermann et al., 1997) |
|  |  |  | Rabies | R | Rat | (Ogawa et al., 2014) |
|  |  |  | CTb | R | Rat | (Trevizan-Baú et al., 2021b) |
|  | cVRG | Glu | WGA-HRP | R | Cat | (Holstege, 1989) |
|  |  |  | WGA-HRP | R | Cat | (Gerrits and Holstege, 1996) |
|  |  |  | WGA-HRP | A | Monkey^3^ | (Vanderhorst et al., 2000) |
|  |  |  | WGA-HRP | R | Monkey^3^ | (Vanderhorst et al., 2000) |
|  |  |  | BDA | A | Rat | (Oka et al., 2008) |
|  |  |  | CTb | R | Rat | (Oka et al., 2008) |
|  | Central amygdala |  | WGA-HRP | AR | Rat | (Rizvi et al., 1991) |
|  | Bed n. stria term. | DA | CTb | R | Rat | (Shin et al., 2008) |
|  |  |  | FG | R | Tree shrew^1^ | (Ni et al., 2016) |
|  | Lateral hypothalamus |  | PI/TB/EB | R | Rat | (Woolf and Butcher, 1986) |
|  |  |  | CTb | R | Rat | (Yoshida et al., 2006) |
|  | Basal pons |  | WGA-HRP | R | Rat | (Mihailoff et al., 1989) |
|  | Inferior olive |  | HRP | R | Rat | (Brown et al., 1977) |
|  |  |  | HRP | R | Rat | (Swenson and Castro, 1983) |
|  |  |  | WGA-HRP | A | Monkey^3^ | (Vanderhorst et al., 2000) |
|  | Vestibular nuclei |  | Rabies | R | Mouse | (Shi et al., 2021) |
|  | Kölliker-Fuse nucleus |  | CTb | R | Rat | (Trevizan-Baú et al., 2021b) |
|  | PPTg |  | WGA-HRP | R | Rat | (Semba and Fibiger, 1992) |
|  |  |  | WGA-HRP | R | Rat | (Steininger et al., 1992) |
| PPTg | Trigeminal motor n. | ACh | EB/FG/TB | R | Rat | (Woolf and Butcher, 1989) |
|  | Facial nucleus | ACh | EB/FG/TB | R | Rat | (Woolf and Butcher, 1989) |
|  | Hypoglossal nucleus | ACh | EB/FG/TB | R | Rat | (Woolf and Butcher, 1989) |
|  | Lateral parafacial n. | GABA  Glu | HSV | R | Rat | (Biancardi et al., 2021) |
|  | Retrotrapezoid n. | ACh | PHA-L | A | Rat | (Lima et al., 2019) |
|  |  |  | FG | R | Rat | (Lima et al., 2019) |
|  |  |  | FG | R | Rat | (Silva et al., 2019) |
|  | Locus coeruleus | ACh | EB/FG/TB | R | Rat | (Woolf and Butcher, 1989) |
|  | Dorsal raphe | ACh | Rabies | R | Rat | (Ogawa et al., 2014) |
|  | Caudal raphe | ACh | EB/FG/TB | R | Rat | (Woolf and Butcher, 1989) |
|  |  |  | CTb | R | Rat | (Hermann et al., 1997) |
|  |  |  | Rabies | R | Rat | (Ogawa et al., 2014) |
|  |  |  | PHA-L | A | Rat | (Lima et al., 2019) |
|  | Spinal trigeminal n. | ACh | EB/FG/TB | R | Rat | (Woolf and Butcher, 1989) |
|  | Reticular formation | ACh | EB/FG/TB | R | Rat | (Woolf and Butcher, 1989) |
|  |  |  | FG | R | Rat | (Ruggiero et al., 1997) |
|  | Central amygdala | ACh | AAV | A | Rat | (Dautan et al., 2016) |
|  | Bed n. stria term. | ACh | FG | R | Tree shrew^1^ | (Ni et al., 2016) |
|  | Lateral hypothalamus | ACh | PI/TB/EB | R | Rat | (Woolf and Butcher, 1986) |
|  | Fastigial nucleus | ACh | FG | R | Rat | (Ruggiero et al., 1997) |
|  |  |  | E-phys |  | Rat | (Vitale et al., 2016) |
|  | Interposed nucleus | ACh | E-phys |  | Rat | (Vitale et al., 2016) |
|  | Dentate nucleus | ACh | E-phys |  | Rat | (Vitale et al., 2016) |
|  | Cerebellar nuclei  *Not specified* | ACh | EB/FG/TB | R | Rat | (Woolf and Butcher, 1989) |
|  | Basal pons | ACh | WGA-HRP | R | Rat | (Mihailoff et al., 1989) |
|  | Kölliker-Fuse nucleus | ACh | PHA-L | A | Rat | (Lima et al., 2019) |
|  | Lateral parabrachial n. | ACh | Microbead | R | Cat | (Quattrochi et al., 1998) |
|  |  |  | PHA-L | A | Rat | (Lima et al., 2019) |

**Supplementary File 1a – Monosynaptic projections in the respiratory control system**

List of studies demonstrating the existence of monosynaptic projections between areas relevant for autonomic control of respiration. Connections that were identified as sparse by the authors of these studies are not listed in this table. Note that the list of neurotransmitters involved is not complete. ^1^ Tree shrew (*Tupaia belangeri chinensis*); ^2^ Rufous horseshoe bat (*Rhinolophus rouxi*); ^3^ Rhesus monkey (*Macaca mulatta*); ^4^ Crab-eating macaque (*Macaca fascicularis*); ^5^ Squirrel monkey (*Saimiri sciureus*). 5-HT = serotonin, A = anterograde, aa = amino acids, AAV = adeno associated virus, ACh = acetyl choline, BDA = biotinylated dextran amines, Biotinam = biotinamide, CRH = corticotropin-releasing hormone, CTb = cholera toxin B subunit, cVRG = caudal part of the ventral respiratory group, DA = dopamine, Degen = degeneration, DTR = dextran-Texas red, DY = diamidino yellow, EB = Evans blue, Exc = excitatory (not specified which neurotransmitter), E-phys = electrophysiology, FB = fast blue, FG = fluorogold, FR = fluoro-ruby, GABA = γ-aminobutyric acid, GFP = green fluorescent protein, Glu = glutamate, Gly = glycine, HSV = herpes simplex virus, HRP = horseradish peroxidase, Inh = inhibitory (not specified which neurotransmitter), m = muscle, MDJ = nuclei of the meso-diencephalic junction, n = nucleus or nuclei, NA = noradrenaline / norepinephrine, Nbiotin = neurobiotin, NMB = neuromedin B, NT = neurotransmitter, NTS = nucleus tractus solitarii, Optogen = optogenetic stimulation, OXT = oxytocin, PHA-L = *Phaseolus vulgaris* leucoagglutinin, PiCo = post-inspiration complex, PPTg = pedunculopontine tegmental nucleus, PI = propidium iodide, POMC = pro-opiomelanocortin, Pseudorab = Pseudorabies, R = retrograde, rVRG = rostral part of the ventral respiratory group, term = terminalis, TMR = tetramethylrhodamine, VP = vasopressin, WGA = wheat germ agglutinin.

| Disease or syndrome | System | Affected area | Study | Reference |
| --- | --- | --- | --- | --- |
| SCA2 | Motor areas | Thoracic spinal cord | Post mortem | (Estrada et al., 1999) |
|  |  | Facial nucleus | Post mortem | (Gierga et al., 2005) |
|  |  |  | Post mortem | (Rüb et al., 2006) |
|  |  | Hypoglossal nucleus | Post mortem | (Gierga et al., 2005) |
|  |  |  | Post mortem | (Rüb et al., 2006) |
|  |  | Trigeminal motor nucleus | Post mortem | (Gierga et al., 2005) |
|  |  |  | Post mortem | (Rüb et al., 2006) |
|  |  | Ambiguus nucleus | Post mortem | (Gierga et al., 2005) |
|  |  |  | Post mortem | (Rüb et al., 2006) |
|  | Central chemoreceptor areas | NTS | Post mortem | (Gierga et al., 2005) |
|  |  |  | Post mortem | (Rüb et al., 2006) |
|  |  | Locus coeruleus | Post mortem | (Estrada et al., 1999) |
|  | Other sensory areas | Clarke’s column | Post mortem | (Estrada et al., 1999) |
|  |  | Spinal trigeminal nucleus | Post mortem | (Gierga et al., 2005) |
|  |  |  | Post mortem | (Rüb et al., 2006) |
|  | Pre-motor areas | Reticular formation | Post mortem | (Rüb et al., 2005) |
|  |  |  | Post mortem | (Rüb et al., 2006) |
|  | Cerebellum-related areas | Basal pons | Post mortem | (Estrada et al., 1999) |
|  |  |  | Post mortem | (Estrada et al., 1999) |
|  |  |  | Post mortem | (Rüb et al., 2005) |
|  |  | Cerebellar cortex | MRI | (Bürk et al., 1996) |
|  |  |  | Post mortem | (Estrada et al., 1999) |
|  |  |  | MRI | (Della Nave et al., 2008) |
|  |  |  | Post mortem | (Scherzed et al., 2012) |
|  |  | Cerebellar nuclei | Post mortem | (Scherzed et al., 2012) |
|  |  | Inferior olive | Post mortem | (Estrada et al., 1999) |
|  |  |  | Post mortem | (Rüb et al., 2005) |
|  |  | Vestibular nuclei | Post mortem | (Gierga et al., 2005) |
|  |  |  | Post mortem | (Rüb et al., 2005) |
|  |  |  |  |  |
| SCA3 | Motor areas | Facial nucleus | Post mortem | (Rüb et al., 2006) |
|  |  | Hypoglossal nucleus | Post mortem | (Rüb et al., 2006) |
|  |  | Trigeminal motor nucleus | Post mortem | (Rüb et al., 2006) |
|  |  | Ambiguus nucleus | Post mortem | (Rüb et al., 2006) |
|  |  |  | Post mortem | (Schwarzacher et al., 2011) |
|  | Other sensory areas | Clarke’s column | Post mortem | (Dürr et al., 1996) |
|  |  | Spinal trigeminal nucleus | Post mortem | (Rüb et al., 2006) |
|  | Pre-motor areas | Cervical spinal cord | MRI | (Lukas et al., 2008) |
|  |  | Reticular formation | Post mortem | (Rüb et al., 2005) |
|  |  |  | Post mortem | (Rüb et al., 2006) |
|  | Cerebellum-related areas | Basal pons | Post mortem | (Dürr et al., 1996) |
|  |  |  | MRI | (Bürk et al., 1996) |
|  |  |  | Post mortem | (Rüb et al., 2005) |
|  |  | Cerebellar cortex | MRI | (Bürk et al., 1996) |
|  |  |  | MRI | (Murata et al., 1998b) |
|  |  |  | Post mortem | (Estrada et al., 1999) |
|  |  |  | Post mortem | (Scherzed et al., 2012) |
|  |  | Cerebellar nuclei | Post mortem | (Dürr et al., 1996) |
|  |  |  | Post mortem | (Scherzed et al., 2012) |
|  |  |  | Post mortem | (Koeppen, 2018) |
|  |  | Inferior olive | Post mortem | (Rüb et al., 2005) |
|  |  | Vestibular nuclei | Post mortem | (Rüb et al., 2005) |
|  |  |  |  |  |
| SCA6 | Cerebellum-related areas | Cerebellar cortex | Post mortem | (Gomez et al., 1997) |
|  |  |  | MRI | (Murata et al., 1998a) |
|  |  |  | Post mortem | (Ishikawa et al., 1999) |
|  |  | Cerebellar nuclei | Post mortem | (Gomez et al., 1997) |
|  |  |  |  |  |
| SCA7 | Motor areas | Hypoglossal nucleus | Post mortem | (Horton et al., 2013) |
|  | Cerebellum-related areas | Cerebellar cortex | MRI | (Horton et al., 2013) |
|  |  |  | Post mortem | (Horton et al., 2013) |
|  |  |  | MRI | (Hernandez-Castillo et al., 2016) |
|  |  |  | MRI | (Chirino et al., 2018) |
|  |  | Cerebellar nuclei | Post mortem | (Horton et al., 2013) |
|  |  | Basal pons | MRI | (Bang et al., 2004) |
|  |  |  | MRI | (Horton et al., 2013) |
|  |  |  | Post mortem | (Horton et al., 2013) |
|  |  | Inferior olive | MRI | (Horton et al., 2013) |
|  |  |  | Post mortem | (Horton et al., 2013) |
|  |  |  |  |  |
| CANVAS | Cerebellum-related areas | Cerebellar cortex | Post mortem | (Szmulewicz et al., 2014) |
|  |  |  |  |  |
| SIDS | Central chemoreceptor areas | Caudal raphe | Post mortem | (Paterson et al., 2006) |
|  | Cerebellum-related areas | Cerebellar cortex | Post mortem | (Lavezzi et al., 2013) |
|  |  |  | Post mortem | (Matschke et al., 2020) |

**Supplementary File 1b – Respiratory control areas affected by pathology**

Summary of brain regions involved in respiratory control and affected by selected diseases or syndromes. An area is listed in this table if a study reported damage to that area in a majority of subjects included in that study. Case studies with a single patient are not included in this table.

**Supplementary references**

Anton F, Peppel P (1991) Central projections of trigeminal primary afferents innervating the nasal mucosa: a horseradish peroxidase study in the rat. Neuroscience 41:617-628.

Arima Y, Yokota S, Fujitani M (2019) Lateral parabrachial neurons innervate orexin neurons projecting to brainstem arousal areas in the rat. Sci Rep 9:2830.

Bácskai T, Székely G, Matesz C (2002) Ascending and descending projections of the lateral vestibular nucleus in the rat. Acta Biol Hung 53:7-21.

Bagnall MW, Zingg B, Sakatos A, Moghadam SH, Zeilhofer HU, du Lac S (2009) Glycinergic projection neurons of the cerebellum. J Neurosci 29:10104-10110.

Balaban CD, Beryozkin G (1994) Vestibular nucleus projections to nucleus tractus solitarius and the dorsal motor nucleus of the vagus nerve: potential substrates for vestibulo-autonomic interactions. Exp Brain Res 98:200-212.

Bang OY, Lee PH, Kim SY, Kim HJ, Huh K (2004) Pontine atrophy precedes cerebellar degeneration in spinocerebellar ataxia 7: MRI-based volumetric analysis. Journal of neurology, neurosurgery, and psychiatry 75:1452-1456.

Bang SJ, Commons KG (2012) Forebrain GABAergic projections from the dorsal raphe nucleus identified by using GAD67-GFP knock-in mice. J Comp Neurol 520:4157-4167.

Barbier M, Gonzalez JA, Houdayer C, Burdakov D, Risold PY, Croizier S (2021) Projections from the dorsomedial division of the bed nucleus of the stria terminalis to hypothalamic nuclei in the mouse. J Comp Neurol 529:929-956.

Barmack NH, Baughman RW, Eckenstein FP, Shojaku H (1992) Secondary vestibular cholinergic projection to the cerebellum of rabbit and rat as revealed by choline acetyltransferase immunohistochemistry, retrograde and orthograde tracers. J Comp Neurol 317:250-270.

Batini C, Buisseret-Delmas C, Corvisier J, Hardy O, Jassik-Gerschenfeld D (1978) Brain stem nuclei giving fibers to lobules VI and VII of the cerebellar vermis. Brain Res 153:241-261.

Batton RR, 3rd, Jayaraman A, Ruggiero D, Carpenter MB (1977) Fastigial efferent projections in the monkey: an autoradiographic study. J Comp Neurol 174:281-305.

Beckstead RM, Morse JR, Norgren R (1980) The nucleus of the solitary tract in the monkey: projections to the thalamus and brain stem nuclei. J Comp Neurol 190:259-282.

Beitz AJ (1989) Possible origin of glutamatergic projections to the midbrain periaqueductal gray and deep layer of the superior colliculus of the rat. Brain Res Bull 23:25-35.

Biancardi V, Saini J, Pageni A, Prashaad MH, Funk GD, Pagliardini S (2021) Mapping of the excitatory, inhibitory, and modulatory afferent projections to the anatomically defined active expiratory oscillator in adult male rats. J Comp Neurol 529:853-884.

Bianchi R, Corsetti G, Rodella L, Tredici G, Gioia M (1998) Supraspinal connections and termination patterns of the parabrachial complex determined by the biocytin anterograde tract-tracing technique in the rat. J Anat 193 ( Pt 3):417-430.

Bienkowski MS, Rinaman L (2013) Common and distinct neural inputs to the medial central nucleus of the amygdala and anterior ventrolateral bed nucleus of stria terminalis in rats. Brain structure & function 218:187-208.

Bishop GA, Ho RH (1985) The distribution and origin of serotonin immunoreactivity in the rat cerebellum. Brain Res 331:195-207.

Bochorishvili G, Stornetta RL, Coates MB, Guyenet PG (2012) Pre-Bötzinger complex receives glutamatergic innervation from galaninergic and other retrotrapezoid nucleus neurons. J Comp Neurol 520:1047-1061.

Boers J, Kirkwood PA, de Weerd H, Holstege G (2006) Ultrastructural evidence for direct excitatory retroambiguus projections to cutaneous trunci and abdominal external oblique muscle motoneurons in the cat. Brain Res Bull 68:249-256.

Borodovitsyna O, Duffy BC, Pickering AE, Chandler DJ (2020) Anatomically and functionally distinct locus coeruleus efferents mediate opposing effects on anxiety-like behavior. Neurobiol Stress 13:100284.

Boulenguez P, Gauthier P, Kastner A (2007) Respiratory neuron subpopulations and pathways potentially involved in the reactivation of phrenic motoneurons after C2 hemisection. Brain Res 1148:96-104.

Brown JT, Chan-Palay V, Palay SL (1977) A study of afferent input to the inferior olivary complex in the rat by retrograde axonal transport of horseradish peroxidase. J Comp Neurol 176:1-22.

Bruinstroop E, Cano G, Vanderhorst VGJM, Cavalcante JC, Wirth J, Sena-Esteves M, Saper CB (2012) Spinal projections of the A5, A6 (locus coeruleus), and A7 noradrenergic cell groups in rats. J Comp Neurol 520:1985-2001.

Brust RD, Corcoran AE, Richerson GB, Nattie E, Dymecki SM (2014) Functional and developmental identification of a molecular subtype of brain serotonergic neuron specialized to regulate breathing dynamics. Cell Rep 9:2152-2165.

Bryant TH, Yoshida S, de Castro D, Lipski J (1993) Expiratory neurons of the Bötzinger Complex in the rat: a morphological study following intracellular labeling with biocytin. J Comp Neurol 335:267-282.

Buisseret-Delmas C, Compoint C, Delfini C, Buisseret P (1999) Organisation of reciprocal connections between trigeminal and vestibular nuclei in the rat. J Comp Neurol 409:153-168.

Bürk K, Abele M, Fetter M, Dichgans J, Skalej M, Laccone F, Didierjean O, Brice A, Klockgether T (1996) Autosomal dominant cerebellar ataxia type I clinical features and MRI in families with SCA1, SCA2 and SCA3. Brain 119:1497-1505.

Caous CA, de Sousa Buck H, Lindsey CJ (2001) Neuronal connections of the paratrigeminal nucleus: a topographic analysis of neurons projecting to bulbar, pontine and thalamic nuclei related to cardiovascular, respiratory and sensory functions. Auton Neurosci 94:14-24.

Carleton SC, Carpenter MB (1984) Distribution of primary vestibular fibers in the brainstem and cerebellum of the monkey. Brain Res 294:281-298.

Çavdar S, Özgur M, Kuvvet Y, Bay HH (2018) The cerebello-hypothalamic and hypothalamo-cerebellar pathways via superior and middle cerebellar peduncle in the rat. Cerebellum 17:517-524.

Cerritelli S, Hirschberg S, Hill R, Balthasar N, Pickering AE (2016) Activation of brainstem pro-opiomelanocortin neurons produces opioidergic analgesia, bradycardia and bradypnoea. PLoS One 11:e0153187.

Chamberlin NL, Eikermann M, Fassbender P, White DP, Malhotra A (2007) Genioglossus premotoneurons and the negative pressure reflex in rats. J Physiol 579:515-526.

Chaumont J, Guyon N, Valera AM, Dugué GP, Popa D, Marcaggi P, Gautheron V, Reibel-Foisset S, Dieudonné S, Stephan A, Barrot M, Cassel JC, Dupont JL, Doussau F, Poulain B, Selimi F, Léna C, Isope P (2013) Clusters of cerebellar Purkinje cells control their afferent climbing fiber discharge. Proc Natl Acad Sci U S A 110:16223-16228.

Chen Z, Lin MT, Zhan C, Zhong NS, Mu D, Lai KF, Liu MJ (2022) A descending pathway emanating from the periaqueductal gray mediates the development of cough-like hypersensitivity. iScience 25:103641.

Chirino A, Hernandez-Castillo CR, Galvez V, Contreras A, Diaz R, Beltran-Parrazal L, Fernandez-Ruiz J (2018) Motor and cognitive impairments in spinocerebellar ataxia type 7 and its correlations with cortical volumes. Eur J Neurosci 48:3199-3211.

Ciriello J, Caverson MM (2014) Hypothalamic orexin-A (hypocretin-1) neuronal projections to the vestibular complex and cerebellum in the rat. Brain Res 1579:20-34.

Claps A, Torrealba F (1988) The carotid body connections: a WGA-HRP study in the cat. Brain Res 455:123-133.

Cunningham ET, Jr., Sawchenko PE (1988) Anatomical specificity of noradrenergic inputs to the paraventricular and supraoptic nuclei of the rat hypothalamus. J Comp Neurol 274:60-76.

Dautan D, Hacioglu Bay H, Bolam JP, Gerdjikov TV, Mena-Segovia J (2016) Extrinsic sources of cholinergic innervation of the striatal complex: A whole-brain mapping analysis. Front Neuroanat 10:1.

Dauvergne C, Pinganaud G, Buisseret P, Buisseret-Delmas C, Zerari-Mailly F (2001) Reticular premotor neurons projecting to both facial and hypoglossal nuclei receive trigeminal afferents in rats. Neurosci Lett 311:109-112.

de Sousa Buck H, Caous CA, Lindsey CJ (2001) Projections of the paratrigeminal nucleus to the ambiguus, rostroventrolateral and lateral reticular nuclei, and the solitary tract. Auton Neurosci 87:187-200.

De Zeeuw CI, Berrebi AS (1995) Postsynaptic targets of Purkinje cell terminals in the cerebellar and vestibular nuclei of the rat. Eur J Neurosci 7:2322-2333.

Della Nave R, Ginestroni A, Tessa C, Cosottini M, Giannelli M, Salvatore E, Sartucci F, De Michele G, Dotti MT, Piacentini S, Mascalchi M (2008) Brain structural damage in spinocerebellar ataxia type 2. A voxel-based morphometry study. Mov Disord 23:899-903.

Depuy SD, Kanbar R, Coates MB, Stornetta RL, Guyenet PG (2011) Control of breathing by raphe obscurus serotonergic neurons in mice. J Neurosci 31:1981-1990.

Dietrichs E (1984) Cerebellar autonomic function: direct hypothalamocerebellar pathway. Science 223:591-593.

Dietrichs E (1988) Cerebellar cortical and nuclear afferents from the feline locus coeruleus complex. Neuroscience 27:77-91.

Do J, Chang Z, Sekerkova G, McCrimmon DR, Martina M (2020) A leptin-mediated neural mechanism linking breathing to metabolism. Cell Rep 33:108358.

Dobbins EG, Feldman JL (1994) Brainstem network controlling descending drive to phrenic motoneurons in rat. J Comp Neurol 347:64-86.

Dong HW, Petrovich GD, Watts AG, Swanson LW (2001) Basic organization of projections from the oval and fusiform nuclei of the bed nuclei of the stria terminalis in adult rat brain. J Comp Neurol 436:430-455.

Driessen AK, Farrell MJ, Dutschmann M, Stanic D, McGovern AE, Mazzone SB (2018) Reflex regulation of breathing by the paratrigeminal nucleus via multiple bulbar circuits. Brain structure & function 223:4005-4022.

Dürr A, Stevanin G, Cancel G, Duyckaerts C, Abbas N, Didierjean O, Chneiweiss H, Benomar A, Lyon-Caen O, Julien J, Serdaru M, Penet C, Agid Y, Brice A (1996) Spinocerebellar ataxia 3 and Machado-Joseph disease: clinical, molecular, and neuropathological features. Ann Neurol 39:490-499.

Elbaz M, Callado Perez A, Demers M, Zhao S, Foo C, Kleinfeld D, Deschenes M (2022) A vibrissa pathway that activates the limbic system. Elife 11.

Ellenberger HH, Feldman JL (1988) Monosynaptic transmission of respiratory drive to phrenic motoneurons from brainstem bulbospinal neurons in rats. J Comp Neurol 269:47-57.

Ellenberger HH, Feldman JL (1990) Brainstem connections of the rostral ventral respiratory group of the rat. Brain Res 513:35-42.

Ellenberger HH, Feldman JL, Goshgarian HG (1990a) Ventral respiratory group projections to phrenic motoneurons: electron microscopic evidence for monosynaptic connections. J Comp Neurol 302:707-714.

Ellenberger HH, Vera PL, Haselton JR, Haselton CL, Schneiderman N (1990b) Brainstem projections to the phrenic nucleus: an anterograde and retrograde HRP study in the rabbit. Brain Res Bull 24:163-174.

Estrada R, Galarraga J, Orozco G, Nodarse A, Auburger G (1999) Spinocerebellar ataxia 2 (SCA2): morphometric analyses in 11 autopsies. Acta neuropathologica 97:306-310.

Ezure K, Tanaka I (1996) Pump neurons of the nucleus of the solitary tract project widely to the medulla. Neurosci Lett 215:123-126.

Ezure K, Tanaka I, Saito Y (2003) Brainstem and spinal projections of augmenting expiratory neurons in the rat. Neurosci Res 45:41-51.

Fedorko L, Merrill EG (1984) Axonal projections from the rostral expiratory neurones of the Botzinger complex to medulla and spinal cord in the cat. J Physiol 350:487-496.

Fedorko L, Merrill EG, Lipski J (1983) Two descending medullary inspiratory pathways to phrenic motoneurones. Neurosci Lett 43:285-291.

Finley JCW, Katz DM (1992) The central organization of carotid body afferent projections to the brainstem of the rat. Brain Res 572:108-116.

Frontera JL, Baba Aissa H, Sala RW, Mailhes-Hamon C, Georgescu IA, Léna C, Popa D (2020) Bidirectional control of fear memories by cerebellar neurons projecting to the ventrolateral periaqueductal grey. Nat Commun 11:5207.

Fu JY, Yu XD, Zhu Y, Xie SZ, Tang MY, Yu B, Li XM (2020) Whole-brain map of long-range monosynaptic inputs to different cell types in the amygdala of the mouse. Neurosci Bull 36:1381-1394.

Fu Y, Tvrdik P, Makki N, Paxinos G, Watson C (2011) Precerebellar cell groups in the hindbrain of the mouse defined by retrograde tracing and correlated with cumulative Wnt1-cre genetic labeling. Cerebellum 10:570-584.

Fujita H, Kodama T, du Lac S (2020) Modular output circuits of the fastigial nucleus for diverse motor and nonmotor functions of the cerebellar vermis. Elife 9:e58613.

Fulwiler CE, Saper CB (1984) Subnuclear organization of the efferent connections of the parabrachial nucleus in the rat. Brain Res 319:229-259.

Fung SJ, Yamuy J, Sampogna S, Morales FR, Chase MH (2001) Hypocretin (orexin) input to trigeminal and hypoglossal motoneurons in the cat: a double-labeling immunohistochemical study. Brain Res 903:257-262.

Gacek RR (1969) The course and central termination of first order neurons supplying vestibular endorgans in the cat. Acta Otolaryngol Suppl 254:1-66.

Gang S, Sato Y, Kohama I, Aoki M (1995) Afferent projections to the Bötzinger complex from the upper cervical cord and other respiratory related structures in the brainstem in cats: retrograde WGA-HRP tracing. J Auton Nerv Syst 56:1-7.

Gao Z, Proietti-Onori M, Lin Z, Ten Brinke MM, Boele HJ, Potters JW, Ruigrok TJH, Hoebeek FE, De Zeeuw CI (2016) Excitatory cerebellar nucleocortical circuit provides internal amplification during associative conditioning. Neuron 89:645-657.

Gasparini S, Howland JM, Thatcher AJ, Geerling JC (2020) Central afferents to the nucleus of the solitary tract in rats and mice. J Comp Neurol 528:2708-2728.

Gaytán SP, Pásaro R (1998) Connections of the rostral ventral respiratory neuronal cell group: an anterograde and retrograde tracing study in the rat. Brain Res Bull 47:625-642.

Geerling JC, Shin JW, Chimenti PC, Loewy AD (2010) Paraventricular hypothalamic nucleus: axonal projections to the brainstem. J Comp Neurol 518:1460-1499.

Geerling JC, Yokota S, Rukhadze I, Roe D, Chamberlin NL (2017) Kölliker-Fuse GABAergic and glutamatergic neurons project to distinct targets. J Comp Neurol 525:1844-1860.

Gerrits NM, Voogd J, Magras IN (1985) Vestibular afferents of the inferior olive and the vestibulo-olivo-cerebellar climbing fiber pathway to the flocculus in the cat. Brain Res 332:325-336.

Gerrits PO, Holstege G (1996) Pontine and medullary projections to the nucleus retroambiguus: a wheat germ agglutinin-horseradish peroxidase and autoradiographic tracing study in the cat. J Comp Neurol 373:173-185.

Gierga K, Bürk K, Bauer M, Orozco Diaz G, Auburger G, Schultz C, Vuksic M, Schöls L, de Vos RAI, Braak H, Deller T, Rüb U (2005) Involvement of the cranial nerves and their nuclei in spinocerebellar ataxia type 2 (SCA2). Acta neuropathologica 109:617-631.

Gomez CM, Thompson RM, Gammack JT, Perlman SL, Dobyns WB, Truwit CL, Zee DS, Clark HB, Anderson JH (1997) Spinocerebellar ataxia type 6: gaze-evoked and vertical nystagmus, Purkinje cell degeneration, and variable age of onset. Ann Neurol 42:933-950.

Gould BB, Graybiel AM (1976) Afferents to the cerebellar cortex in the cat: evidence for an intrinsic pathway leading from the deep nuclei to the cortex. Brain Res 110:601-611.

Guo H, Yuan XS, Zhou JC, Chen H, Li SQ, Qu WM, Huang ZL (2020) Whole-brain monosynaptic inputs to hypoglossal motor neurons in mice. Neurosci Bull 36:585-597.

Halberstadt AL, Balaban CD (2006) Serotonergic and nonserotonergic neurons in the dorsal raphe nucleus send collateralized projections to both the vestibular nuclei and the central amygdaloid nucleus. Neuroscience 140:1067-1077.

Hashimoto M, Yamanaka A, Kato S, Tanifuji M, Kobayashi K, Yaginuma H (2018) Anatomical evidence for a direct projection from Purkinje cells in the mouse cerebellar vermis to medial parabrachial nucleus. Frontiers in neural circuits 12:6.

Hayakawa T, Takanaga A, Maeda S, Ito H, Seki M (2000) Monosynaptic inputs from the nucleus tractus solitarii to the laryngeal motoneurons in the nucleus ambiguus of the rat. Anat Embryol (Berl) 202:411-420.

Hennessy ML, Corcoran AE, Brust RD, Chang Y, Nattie EE, Dymecki SM (2017) Activity of *Tachykinin1*-expressing *Pet1* raphe neurons modulates the respiratory chemoreflex. J Neurosci 37:1807-1819.

Henschke JU, Pakan JM (2020) Disynaptic cerebrocerebellar pathways originating from multiple functionally distinct cortical areas. Elife 9.

Herbert H, Saper CB (1992) Organization of medullary adrenergic and noradrenergic projections to the periaqueductal gray matter in the rat. J Comp Neurol 315:34-52.

Herbert H, Moga MM, Saper CB (1990) Connections of the parabrachial nucleus with the nucleus of the solitary tract and the medullary reticular formation in the rat. J Comp Neurol 293:540-580.

Hermann DM, Luppi PH, Peyron C, Hinckel P, Jouvet M (1997) Afferent projections to the rat nuclei raphe magnus, raphe pallidus and reticularis gigantocellularis pars a demonstrated by iontophoretic application of choleratoxin (subunit b). Journal of Chemical Neuroanatomy 13:1-21.

Hernandez-Castillo CR, Galvez V, Diaz R, Fernandez-Ruiz J (2016) Specific cerebellar and cortical degeneration correlates with ataxia severity in spinocerebellar ataxia type 7. Brain Imaging Behav 10:252-257.

Holstege G (1989) Anatomical study of the final common pathway for vocalization in the cat. J Comp Neurol 284:242-252.

Horton LC, Frosch MP, Vangel MG, Weigel-DiFranco C, Berson EL, Schmahmann JD (2013) Spinocerebellar ataxia type 7: clinical course, phenotype-genotype correlations, and neuropathology. Cerebellum 12:176-193.

Huang CC, Sugino K, Shima Y, Guo C, Bai S, Mensh BD, Nelson SB, Hantman AW (2013) Convergence of pontine and proprioceptive streams onto multimodal cerebellar granule cells. Elife 2:e00400.

Huerta MF, Hashikawa T, Gayoso MJ, Harting JK (1985) The trigemino-olivary projection in the cat: contributions of individual subnuclei. J Comp Neurol 241:180-190.

Ishikawa K, Watanabe M, Yoshizawa K, Fujita T, Iwamoto H, Yoshizawa T, Harada K, Nakamagoe K, Komatsuzaki Y, Satoh A, Doi M, Ogata T, Kanazawa I, Shoji S, Mizusawa H (1999) Clinical, neuropathological, and molecular study in two families with spinocerebellar ataxia type 6 (SCA6). Journal of neurology, neurosurgery, and psychiatry 67:86-89.

Jian BJ, Acernese AW, Lorenzo J, Card JP, Yates BJ (2005) Afferent pathways to the region of the vestibular nuclei that participates in cardiovascular and respiratory control. Brain Res 1044:241-250.

Jiang C, Lipski J (1990) Extensive monosynaptic inhibition of ventral respiratory group neurons by augmenting neurons in the Bötzinger complex in the cat. Exp Brain Res 81:639-648.

Jones BE, Moore RY (1977) Ascending projections of the locus coeruleus in the rat. II. Autoradiographic study. Brain Res 127:25-53.

Jones BE, Yang TZ (1985) The efferent projections from the reticular formation and the locus coeruleus studied by anterograde and retrograde axonal transport in the rat. J Comp Neurol 242:56-92.

Jones SE, Stanic D, Dutschmann M (2016) Dorsal and ventral aspects of the most caudal medullary reticular formation have differential roles in modulation and formation of the respiratory motor pattern in rat. Brain structure & function 221:4353-4368.

Judd EN, Lewis SM, Person AL (2021) Diverse inhibitory projections from the cerebellar interposed nucleus. Elife 10.

Kaur S, De Luca R, Khanday MA, Bandaru SS, Thomas RC, Broadhurst RY, Venner A, Todd WD, Fuller PM, Arrigoni E, Saper CB (2020) Role of serotonergic dorsal raphe neurons in hypercapnia-induced arousals. Nat Commun 11:2769.

Kawai Y (2018) Differential ascending projections from the male rat caudal nucleus of the tractus solitarius: An interface between local microcircuits and global macrocircuits. Front Neuroanat 12:63.

Kc P, Haxhiu MA, Trouth CO, Balan KV, Anderson WA, Mack SO (2002) CO_2_-induced c-Fos expression in hypothalamic vasopressin containing neurons. Respiration physiology 129:289-296.

Keay KA, Feil K, Gordon BD, Herbert H, Bandler R (1997) Spinal afferents to functionally distinct periaqueductal gray columns in the rat: an anterograde and retrograde tracing study. J Comp Neurol 385:207-229.

King TL, Heesch CM, Clark CG, Kline DD, Hasser EM (2012) Hypoxia activates nucleus tractus solitarii neurons projecting to the paraventricular nucleus of the hypothalamus. Am J Physiol Regul Integr Comp Physiol 302:R1219-1232.

Koeppen AH (2018) The neuropathology of spinocerebellar ataxia type 3/Machado-Joseph disease. Advances in Experimental Medicine and Biology 1049:233-241.

Koshiya N, Oku Y, Yokota S, Oyamada Y, Yasui Y, Okada Y (2014) Anatomical and functional pathways of rhythmogenic inspiratory premotor information flow originating in the pre-Bötzinger complex in the rat medulla. Neuroscience 268:194-211.

Kubo R, Aiba A, Hashimoto K (2018) The anatomical pathway from the mesodiencephalic junction to the inferior olive relays perioral sensory signals to the cerebellum in the mouse. J Physiol 596:3775-3791.

Lane MA, White TE, Coutts MA, Jones AL, Sandhu MS, Bloom DC, Bolser DC, Yates BJ, Fuller DD, Reier PJ (2008) Cervical prephrenic interneurons in the normal and lesioned spinal cord of the adult rat. J Comp Neurol 511:692-709.

Lavezzi AM, Corna MF, Repetti ML, Matturri L (2013) Cerebellar Purkinje cell vulnerability to prenatal nicotine exposure in sudden unexplained perinatal death. Folia Neuropathol 51:290-301.

Lee HS, Kim MA, Valentino RJ, Waterhouse BD (2003) Glutamatergic afferent projections to the dorsal raphe nucleus of the rat. Brain Res 963:57-71.

Lee HS, Park SH, Song WC, Waterhouse BD (2005) Retrograde study of hypocretin-1 (orexin-A) projections to subdivisions of the dorsal raphe nucleus in the rat. Brain Res 1059:35-45.

Li F, Jiang H, Shen X, Yang W, Guo C, Wang Z, Xiao M, Cui L, Luo W, Kim BS, Chen Z, Huang AJW, Liu Q (2021) Sneezing reflex is mediated by a peptidergic pathway from nose to brainstem. Cell 184:3762-3773 e3710.

Li P, Janczewski WA, Yackle K, Kam K, Pagliardini S, Krasnow MA, Feldman JL (2016) The peptidergic control circuit for sighing. Nature 530:293-297.

Li Q, Song G (2001) Afferent projection to the retrotrapezoid nucleus from respiratory related structures in the brainstem of rabbit--a retrograde CB-HRP tracing study. Sheng Li Xue Bao 53:401-404.

Li YQ, Takada M, Mizuno N (1993) The sites of origin of serotoninergic afferent fibers in the trigeminal motor, facial, and hypoglossal nuclei in the rat. Neurosci Res 17:307-313.

Lima JD, Sobrinho CR, Falquetto B, Santos LK, Takakura AC, Mulkey DK, Moreira TS (2019) Cholinergic neurons in the pedunculopontine tegmental nucleus modulate breathing in rats by direct projections to the retrotrapezoid nucleus. J Physiol 597:1919-1934.

Lindeman S, Hong S, Kros L, Mejias JF, Romano V, Oostenveld R, Negrello M, Bosman LWJ, De Zeeuw CI (2021) Cerebellar Purkinje cells can differentially modulate coherence between sensory and motor cortex depending on region and behavior. Proc Natl Acad Sci U S A 118:e2015292118.

Lipski J, Zhang X, Kruszewska B, Kanjhan R (1994) Morphological study of long axonal projections of ventral medullary inspiratory neurons in the rat. Brain Research 640:171-184.

Liu H, Mihailoff GA (1999) Hypothalamopontine projections in the rat: anterograde axonal transport studies utilizing light and electron microscopy. Anat Rec 255:428-451.

Liu J, Hu T, Zhang MQ, Xu CY, Yuan MY, Li RX (2021a) Differential efferent projections of GABAergic neurons in the basolateral and central nucleus of amygdala in mice. Neurosci Lett 745:135621.

Liu N, Fu C, Yu H, Wang Y, Shi L, Hao Y, Yuan F, Zhang X, Wang S (2021b) Respiratory control by Phox2b-expressing neurons in a locus coeruleus-preBötzinger complex circuit. Neurosci Bull 37:31-44.

Livingston CA, Berger AJ (1989) Immunocytochemical localization of GABA in neurons projecting to the ventrolateral nucleus of the solitary tract. Brain Res 494:143-150.

Loewy AD, Burton H (1978) Nuclei of the solitary tract: efferent projections to the lower brain stem and spinal cord of the cat. J Comp Neurol 181:421-449.

Lois JH, Rice CD, Yates BJ (2009) Neural circuits controlling diaphragm function in the cat revealed by transneuronal tracing. J Appl Physiol (1985) 106:138-152.

Loughlin SE, Foote SL, Bloom FE (1986) Efferent projections of nucleus locus coeruleus: topographic organization of cells of origin demonstrated by three-dimensional reconstruction. Neuroscience 18:291-306.

Lu JH, Wang XQ, Huang Y, Qiu YH, Peng YP (2015) GABAergic neurons in cerebellar interposed nucleus modulate cellular and humoral immunity via hypothalamic and sympathetic pathways. Journal of Neuroimmunology 283:30-38.

Lucier GE, Egizii R (1986) Central projections of the ethmoidal nerve of the cat as determined by the horseradish peroxidase tracer technique. J Comp Neurol 247:123-132.

Lukas C, Hahn HK, Bellenberg B, Hellwig K, Globas C, Schimrigk SK, Köster O, Schöls L (2008) Spinal cord atrophy in spinocerebellar ataxia type 3 and 6 : impact on clinical disability. Journal of neurology 255:1244-1249.

Luppi PH, Aston-Jones G, Akaoka H, Chouvet G, Jouvet M (1995) Afferent projections to the rat locus coeruleus demonstrated by retrograde and anterograde tracing with cholera-toxin B subunit and *Phaseolus vulgaris* leucoagglutinin. Neuroscience 65:119-160.

Luskin AT, Bhatti DL, Mulvey B, Pedersen CE, Girven KS, Oden-Brunson H, Kimbell K, Blackburn T, Sawyer A, Gereau RWt, Dougherty JD, Bruchas MR (2021) Extended amygdala-parabrachial circuits alter threat assessment and regulate feeding. Sci Adv 7.

Mack SO, Wu M, Kc P, Haxhiu MA (2007) Stimulation of the hypothalamic paraventricular nucleus modulates cardiorespiratory responses via oxytocinergic innervation of neurons in pre-Botzinger complex. J Appl Physiol (1985) 102:189-199.

Mack SO, Kc P, Wu M, Coleman BR, Tolentino-Silva FP, Haxhiu MA (2002) Paraventricular oxytocin neurons are involved in neural modulation of breathing. J Appl Physiol (1985) 92:826-834.

Mannen H, Sasaki S, Ishizuka N (1982) Trajectory of primary vestibular fibers originating from the lateral, anterior, and posterior semicircular canals in the cat. Proceedings of the Japan Academy, Series B 58:237-242.

Matschke J, Sperhake JP, Wilke N, Püschel K, Glatzel M (2020) Cerebellar heterotopia of infancy in sudden infant death syndrome: an observational neuropathological study of four cases. Int J Legal Med 134:2143-2147.

Matsushita M, Hosoya Y (1979) Cells of origin of the spinocerebellar tract in the rat, studied with the method of retrograde transport of horseradish peroxidase. Brain Res 173:185-200.

Matsushita M, Hosoya Y, Ikeda M (1979) Anatomical organization of the spinocerebellar system in the cat, as studied by retrograde transport of horseradish peroxidase. J Comp Neurol 184:81-106.

Matsushita M, Gao X, Yaginuma H (1995) Spinovestibular projections in the rat, with particular reference to projections from the central cervical nucleus to the lateral vestibular nucleus. J Comp Neurol 361:334-334.

McGovern AE, Driessen AK, Simmons DG, Powell J, Davis-Poynter N, Farrell MJ, Mazzone SB (2015) Distinct brainstem and forebrain circuits receiving tracheal sensory neuron inputs revealed using a novel conditional anterograde transsynaptic viral tracing system. J Neurosci 35:7041-7055.

Menétrey D, Leah J, de Pommery J (1987) Efferent projections of the paratrigeminal nucleus in the rat. Neurosci Lett 73:48-52.

Merrill EG, Fedorko L (1984) Monosynaptic inhibition of phrenic motoneurons: a long descending projection from Bötzinger neurons. J Neurosci 4:2350-2353.

Merrill EG, Lipski J, Kubin L, Fedorko L (1983) Origin of the expiratory inhibition of nucleus tractus solitarius inspiratory neurones. Brain Res 263:43-50.

Mihailoff GA (1993) Cerebellar nuclear projections from the basilar pontine nuclei and nucleus reticularis tegmenti pontis as demonstrated with PHA-L tracing in the rat. J Comp Neurol 330:130-146.

Mihailoff GA, Kosinski RJ, Azizi SA, Border BG (1989) Survey of noncortical afferent projections to the basilar pontine nuclei: a retrograde tracing study in the rat. J Comp Neurol 282:617-643.

Moga MM, Herbert H, Hurley KM, Yasui Y, Gray TS, Saper CB (1990) Organization of cortical, basal forebrain, and hypothalamic afferents to the parabrachial nucleus in the rat. J Comp Neurol 295:624-661.

Molinari HH, Schultze KE, Strominger NL (1996) Gracile, cuneate, and spinal trigeminal projections to inferior olive in rat and monkey. J Comp Neurol 375:467-480.

Moore JD, Deschênes M, Furuta T, Huber D, Smear MC, Demers M, Kleinfeld D (2013) Hierarchy of orofacial rhythms revealed through whisking and breathing. Nature 497:205-210.

Murata Y, Kawakami H, Yamaguchi S, Nishimura M, Kohriyama T, Ishizaki F, Matsuyama Z, Mimori Y, Nakamura S (1998a) Characteristic magnetic resonance imaging findings in spinocerebellar ataxia 6. Arch Neurol 55:1348-1352.

Murata Y, Yamaguchi S, Kawakami H, Imon Y, Maruyama H, Sakai T, Kazuta T, Ohtake T, Nishimura M, Saida T, Chiba S, Oh-i T, Nakamura S (1998b) Characteristic magnetic resonance imaging findings in Machado-Joseph disease. Arch Neurol 55:33-37.

Nagai T, Satoh K, Imamoto K, Maeda T (1981) Divergent projections of catecholamine neurons of the locus coeruleus as revealed by fluorescent retrograde double labeling technique. Neurosci Lett 23:117-123.

Nakayama K, Niwa M, Sasaki SI, Ichikawa T, Hirai N (1998) Morphology of single primary spindle afferents of the intercostal muscles in the cat. J Comp Neurol 398:459-472.

Ni RJ, Luo PH, Shu YM, Chen JT, Zhou JN (2016) Whole-brain mapping of afferent projections to the bed nucleus of the stria terminalis in tree shrews. Neuroscience 333:162-180.

Norgren R (1978) Projections from the nucleus of the solitary tract in the rat. Neuroscience 3:207-218.

Núñez-Abades PA, Portillo F, Pásaro R (1990) Characterisation of afferent projections to the nucleus ambiguus of the rat by means of fluorescent double labelling. J Anat 172:1-15.

Ogawa SK, Cohen JY, Hwang D, Uchida N, Watabe-Uchida M (2014) Organization of monosynaptic inputs to the serotonin and dopamine neuromodulatory systems. Cell Rep 8:1105-1118.

Oka T, Tsumori T, Yokota S, Yasui Y (2008) Neuroanatomical and neurochemical organization of projections from the central amygdaloid nucleus to the nucleus retroambiguus via the periaqueductal gray in the rat. Neurosci Res 62:286-298.

Oliveira LM, Takakura AC, Moreira TS (2021) Forebrain and hindbrain projecting-neurons target the post-inspiratory complex cholinergic neurons. Neuroscience 476:102-115.

Olson L, Fuxe K (1971) On the projections from the locus coeruleus noradrealine neurons: the cerebellar innervation. Brain Res 28:165-171.

Onodera S (1984) Olivary projections from the mesodiencephalic structures in the cat studied by means of axonal transport of horseradish peroxidase and tritiated amino acids. J Comp Neurol 227:37-49.

Panneton WM, Gan Q, Juric R (2006) Brainstem projections from recipient zones of the anterior ethmoidal nerve in the medullary dorsal horn. Neuroscience 141:889-906.

Paterson DS, Trachtenberg FL, Thompson EG, Belliveau RA, Beggs AH, Darnall R, Chadwick AE, Krous HF, Kinney HC (2006) Multiple serotonergic brainstem abnormalities in sudden infant death syndrome. JAMA 296:2124-2132.

Petrov T, Krukoff TL, Jhamandas JH (1992) The hypothalamic paraventricular and lateral parabrachial nuclei receive collaterals from raphe nucleus neurons: a combined double retrograde and immunocytochemical study. J Comp Neurol 318:18-26.

Peyron C, Rampon C, Petit JM, Luppi PH (2018) Sub-regions of the dorsal raphé nucleus receive different inputs from the brainstem. Sleep Med 49:53-63.

Peyron C, Petit JM, Rampon C, Jouvet M, Luppi PH (1998) Forebrain afferents to the rat dorsal raphe nucleus demonstrated by retrograde and anterograde tracing methods. Neuroscience 82:443-468.

Pierce ET, Hoddevik GH, Walberg F (1977) The cerebellar projection from the raphe nuclei in the cat as studied with the method of retrograde transport of horseradish peroxidase. Anat Embryol (Berl) 152:73-87.

Pijpers A, Ruigrok TJH (2006) Organization of pontocerebellar projections to identified climbing fiber zones in the rat. J Comp Neurol 496:513-528.

Pijpers A, Voogd J, Ruigrok TJH (2005) Topography of olivo-cortico-nuclear modules in the intermediate cerebellum of the rat. J Comp Neurol 492:193-213.

Pollak Dorocic I, Fürth D, Xuan Y, Johansson Y, Pozzi L, Silberberg G, Carlén M, Meletis K (2014) A whole-brain atlas of inputs to serotonergic neurons of the dorsal and median raphe nuclei. Neuron 83:663-678.

Pop IV, Espinosa F, Blevins CJ, Okafor PC, Ogujiofor OW, Goyal M, Mona B, Landy MA, Dean KM, Gurumurthy CB, Lai HC (2022) Structure of long-range direct and indirect spinocerebellar pathways as well as local spinal circuits mediating proprioception. J Neurosci 42:581-600.

Quattrochi J, Datta S, Hobson JA (1998) Cholinergic and non-cholinergic afferents of the caudolateral parabrachial nucleus: a role in the long-term enhancement of rapid eye movement sleep. Neuroscience 83:1123-1136.

Ricardo JA, Koh ET (1978) Anatomical evidence of direct projections from the nucleus of the solitary tract to the hypothalamus, amygdala, and other forebrain structures in the rat. Brain Res 153:1-26.

Rikard-Bell GC, Bystrzycka EK, Nail BS (1984) Brainstem projections to the phrenic nucleus: a HRP study in the cat. Brain Res Bull 12:469-477.

Rinaman L (1998) Oxytocinergic inputs to the nucleus of the solitary tract and dorsal motor nucleus of the vagus in neonatal rats. J Comp Neurol 399:101-109.

Rizvi TA, Ennis M, Behbehani MM, Shipley MT (1991) Connections between the central nucleus of the amygdala and the midbrain periaqueductal gray: topography and reciprocity. J Comp Neurol 303:121-131.

Robertson SD, Plummer NW, de Marchena J, Jensen P (2013) Developmental origins of central norepinephrine neuron diversity. Nat Neurosci 16:1016-1023.

Room P, Postema F, Korf J (1981) Divergent axon collaterals of rat locus coeruleus neurons: demonstration by a fluorescent double labeling technique. Brain Res 221:219-230.

Rose PK, Wainwright K, Neuber-Hess M (1992) Connections from the lateral vestibular nucleus to the upper cervical spinal cord of the cat: a study with the anterograde tracer PHA-L. J Comp Neurol 321:312-324.

Rosin DL, Chang DA, Guyenet PG (2006) Afferent and efferent connections of the rat retrotrapezoid nucleus. J Comp Neurol 499:64-89.

Rüb U, Brunt ER, Petrasch-Parwez E, Schöls L, Theegarten D, Auburger G, Seidel K, Schultz C, Gierga K, Paulson H, van Broeckhoven C, Deller T, de Vos RAI (2006) Degeneration of ingestion-related brainstem nuclei in spinocerebellar ataxia type 2, 3, 6 and 7. Neuropathol Appl Neurobiol 32:635-649.

Rüb U, Gierga K, Brunt ER, de Vos RAI, Bauer M, Schöls L, Bürk K, Auburger G, Bohl J, Schultz C, Vuksic M, Burbach GJ, Braak H, Deller T (2005) Spinocerebellar ataxias types 2 and 3: degeneration of the pre-cerebellar nuclei isolates the three phylogenetically defined regions of the cerebellum. J Neural Transm (Vienna) 112:1523-1545.

Rübsamen R, Schweizer H (1986) Control of echolocation pulses by neurons of the nucleus ambiguus in the rufous horseshoe bat, *Rhinolophus rouxi*. II. Afferent and efferent connections of the motor nucleus of the laryngeal nerves. J Comp Physiol A 159:689-699.

Ruggiero D, Batton RR, 3rd, Jayaraman A, Carpenter MB (1977) Brain stem afferents to the fastigial nucleus in the cat demonstrated by transport of horseradish peroxidase. J Comp Neurol 172:189-209.

Ruggiero DA, Anwar M, Golanov EV, Reis DJ (1997) The pedunculopontine tegmental nucleus issues collaterals to the fastigial nucleus and rostral ventrolateral reticular nucleus in the rat. Brain Res 760:272-276.

Ruigrok TJH, Cella F, Voogd J (1995) Connections of the lateral reticular nucleus to the lateral vestibular nucleus in the rat. An anterograde tracing study with *Phaseolus vulgaris* leucoagglutinin. Eur J Neurosci 7:1410-1413.

Ruyle BC, Klutho PJ, Baines CP, Heesch CM, Hasser EM (2018) Hypoxia activates a neuropeptidergic pathway from the paraventricular nucleus of the hypothalamus to the nucleus tractus solitarii. Am J Physiol Regul Integr Comp Physiol 315:R1167-R1182.

Ruyle BC, Martinez D, Heesch CM, Kline DD, Hasser EM (2019) The PVN enhances cardiorespiratory responses to acute hypoxia via input to the nTS. Am J Physiol Regul Integr Comp Physiol 317:R818-R833.

Sadakane K, Kondo M, Nisimaru N (2000) Direct projection from the cardiovascular control region of the cerebellar cortex, the lateral nodulus-uvula, to the brainstem in rabbits. Neurosci Res 36:15-26.

Saigal RP, Karamanlidis AN, Voogd J, Mangana O, Michaloudi H (1980a) Secondary trigeminocerebellar projections in sheep studied with the horseradish peroxidase tracing method. J Comp Neurol 189:537-553.

Saigal RP, Karamanlidis AN, Voogd J, Michaloudi H, Mangana O (1980b) Cerebellar afferents from motor nuclei of cranial nerves, the nucleus of the solitary tract, and nuclei coeruleus and parabrachialis in sheep, demonstrated with retrograde transport of horseradish peroxidase. Brain Res 197:200-206.

Saper CB, Loewy AD (1980) Efferent connections of the parabrachial nucleus in the rat. Brain Res 197:291-317.

Saxon DW, Hopkins DA (1998) Efferent and collateral organization of paratrigeminal nucleus projections: an anterograde and retrograde fluorescent tracer study in the rat. J Comp Neurol 402:93-110.

Scherzed W, Brunt ER, Heinsen H, de Vos RA, Seidel K, Burk K, Schöls L, Auburger G, Del Turco D, Deller T, Korf HW, den Dunnen WF, Rüb U (2012) Pathoanatomy of cerebellar degeneration in spinocerebellar ataxia type 2 (SCA2) and type 3 (SCA3). Cerebellum 11:749-760.

Schwarz LA, Miyamichi K, Gao XJ, Beier KT, Weissbourd B, DeLoach KE, Ren J, Ibanes S, Malenka RC, Kremer EJ, Luo L (2015) Viral-genetic tracing of the input-output organization of a central noradrenaline circuit. Nature 524:88-92.

Schwarzacher SW, Rüb U, Deller T (2011) Neuroanatomical characteristics of the human pre-Bötzinger complex and its involvement in neurodegenerative brainstem diseases. Brain 134:24-35.

Semba K, Fibiger HC (1992) Afferent connections of the laterodorsal and the pedunculopontine tegmental nuclei in the rat: a retro- and antero-grade transport and immunohistochemical study. J Comp Neurol 323:387-410.

Shi X, Wei H, Chen Z, Wang J, Qu W, Huang Z, Dai C (2021) Whole-brain monosynaptic inputs and outputs of glutamatergic neurons of the vestibular nuclei complex in mice. Hear Res 401:108159.

Shin JW, Geerling JC, Loewy AD (2008) Inputs to the ventrolateral bed nucleus of the stria terminalis. J Comp Neurol 511:628-657.

Shinnar S, Maciewicz RJ, Shofer RJ (1975) A raphe projection to cat cerebellar cortex. Brain Res 97:139-143.

Shinoda Y, Sugiuchi Y, Futami T, Izawa R (1992) Axon collaterals of mossy fibers from the pontine nucleus in the cerebellar dentate nucleus. J Neurophysiol 67:547-560.

Silva JN, Tanabe FM, Moreira TS, Takakura AC (2016a) Neuroanatomical and physiological evidence that the retrotrapezoid nucleus/parafacial region regulates expiration in adult rats. Respir Physiol Neurobiol 227:9-22.

Silva JN, Oliveira LM, Souza FC, Moreira TS, Takakura AC (2019) Distinct pathways to the parafacial respiratory group to trigger active expiration in adult rats. Am J Physiol Lung Cell Mol Physiol 317:L402-L413.

Silva JN, Lucena EV, Silva TM, Damasceno RS, Takakura AC, Moreira TS (2016b) Inhibition of the pontine Kölliker-Fuse nucleus reduces genioglossal activity elicited by stimulation of the retrotrapezoid chemoreceptor neurons. Neuroscience 328:9-21.

Singh U, Jiang J, Saito K, Toth BA, Dickey JE, Rodeghiero SR, Deng Y, Deng G, Xue B, Zhu Z, Zingman LV, Geerling JC, Cui H (2022) Neuroanatomical organization and functional roles of PVN MC4R pathways in physiological and behavioral regulations. Mol Metab 55:101401.

Somana R, Walberg F (1979a) Cerebellar afferents from the nucleus of the solitary tract. Neurosci Lett 11:41-47.

Somana R, Walberg F (1979b) The cerebellar projection from the paratrigeminal nucleus in the cat. Neurosci Lett 15:49-54.

Song G, Wang H, Xu H, Poon CS (2012) Kölliker-Fuse neurons send collateral projections to multiple hypoxia-activated and nonactivated structures in rat brainstem and spinal cord. Brain structure & function 217:835-858.

Steininger TL, Rye DB, Wainer BH (1992) Afferent projections to the cholinergic pedunculopontine tegmental nucleus and adjacent midbrain extrapyramidal area in the albino rat. I. Retrograde tracing studies. J Comp Neurol 321:515-543.

Sugihara I, Shinoda Y (2007) Molecular, topographic, and functional organization of the cerebellar nuclei: analysis by three-dimensional mapping of the olivonuclear projection and aldolase C labeling. J Neurosci 27:9696-9710.

Sugihara I, Wu HS, Shinoda Y (2001) The entire trajectories of single olivocerebellar axons in the cerebellar cortex and their contribution to cerebellar compartmentalization. J Neurosci 21:7715-7723.

Sugihara I, Fujita H, Na J, Quy PN, Li BY, Ikeda D (2009) Projection of reconstructed single Purkinje cell axons in relation to the cortical and nuclear aldolase C compartments of the rat cerebellum. J Comp Neurol 512:282-304.

Swenson RS, Castro AJ (1983) The afferent connections of the inferior olivary complex in rats: a study using the retrograde transport of horseradish peroxidase. Am J Anat 166:329-341.

Szentágothai J, Rajkovits K (1959) Über den Ursprung der Kletterfasern des Kleinhirns. Z Anat Entwicklungsgesch 121:130-141.

Szmulewicz DJ, McLean CA, Rodriguez ML, Chancellor AM, Mossman S, Lamont D, Roberts L, Storey E, Halmagyi GM (2014) Dorsal root ganglionopathy is responsible for the sensory impairment in CANVAS. Neurology 82:1410-1415.

Takagi H, Senba E, Shiosaka S, Sakanaka M, Inagaki S, Takatsuki K, Tohyama M (1981) Ascending and cerebellar non-serotonergic projections from the nucleus raphe magnus of the rat. Brain Res 206:161-165.

Takakura AC, Moreira TS, West GH, Gwilt JM, Colombari E, Stornetta RL, Guyenet PG (2007) GABAergic pump cells of solitary tract nucleus innervate retrotrapezoid nucleus chemoreceptors. J Neurophysiol 98:374-381.

Takatoh J, Prevosto V, Thompson PM, Lu J, Chung L, Harrahill A, Li S, Zhao S, He Z, Golomb D, Kleinfeld D, Wang F (2022) The whisking oscillator circuit. Nature 609:560-568.

Tan W, Pagliardini S, Yang P, Janczewski WA, Feldman JL (2010) Projections of preBötzinger complex neurons in adult rats. J Comp Neurol 518:1862-1878.

Teune TM, van der Burg J, van der Moer J, Voogd J, Ruigrok TJ (2000) Topography of cerebellar nuclear projections to the brain stem in the rat. Progress in brain research 124:141-172.

Thompson RH, Swanson LW (1998) Organization of inputs to the dorsomedial nucleus of the hypothalamus: a reexamination with Fluorogold and PHAL in the rat. Brain Res Brain Res Rev 27:89-118.

Thompson RH, Canteras NS, Swanson LW (1996) Organization of projections from the dorsomedial nucleus of the hypothalamus: a PHA-L study in the rat. J Comp Neurol 376:143-173.

Tian GF, Duffin J (1996) Spinal connections of ventral-group bulbospinal inspiratory neurons studied with cross-correlation in the decerebrate rat. Exp Brain Res 111:178-186.

Tian GF, Peever JH, Duffin J (1998) Bötzinger-complex expiratory neurons monosynaptically inhibit phrenic motoneurons in the decerebrate rat. Exp Brain Res 122:149-156.

Tokita K, Inoue T, Boughter JD, Jr. (2010) Subnuclear organization of parabrachial efferents to the thalamus, amygdala and lateral hypothalamus in C57BL/6J mice: a quantitative retrograde double labeling study. Neuroscience 171:351-365.

Tolbert DL, Bantli H, Bloedel JR (1977) The intracerebellar nucleocortical projection in a primate. Exp Brain Res 30:425-434.

Trevizan-Baú P, Dhingra RR, Furuya WI, Stanic D, Mazzone SB, Dutschmann M (2021a) Forebrain projection neurons target functionally diverse respiratory control areas in the midbrain, pons, and medulla oblongata. J Comp Neurol 529:2243-2264.

Trevizan-Baú P, Furuya WI, Mazzone SB, Stanic D, Dhingra RR, Dutschmann M (2021b) Reciprocal connectivity of the periaqueductal gray with the ponto-medullary respiratory network in rat. Brain Res 1757:147255.

Uusisaari M, Knöpfel T (2010) GlyT2+ neurons in the lateral cerebellar nucleus. Cerebellum 9:42-55.

Van Ham JJ, Yeo CH (1992) Somatosensory trigeminal projections to the inferior olive, cerebellum and other precerebellar nuclei in rabbits. Eur J Neurosci 4:302-317.

Vanderhorst VG, Terasawa E, Ralston HJ, 3rd, Holstege G (2000) Monosynaptic projections from the lateral periaqueductal gray to the nucleus retroambiguus in the rhesus monkey: implications for vocalization and reproductive behavior. J Comp Neurol 424:251-268.

VanderHorst VGJM, Terasawa E, Ralston HJ, 3rd (2001) Monosynaptic projections from the nucleus retroambiguus region to laryngeal motoneurons in the rhesus monkey. Neuroscience 107:117-125.

Vertes RP (1991) A PHA-L analysis of ascending projections of the dorsal raphe nucleus in the rat. J Comp Neurol 313:643-668.

Vitale F, Mattei C, Capozzo A, Pietrantoni I, Mazzone P, Scarnati E (2016) Cholinergic excitation from the pedunculopontine tegmental nucleus to the dentate nucleus in the rat. Neuroscience 317:12-22.

Wang X, Novello M, Gao Z, Ruigrok TJH, De Zeeuw CI (2021) Input and output organization of the mesodiencephalic junction for cerebro-cerebellar communication. Journal of Neuroscience Research 100:620-637.

Weissbourd B, Ren J, DeLoach KE, Guenthner CJ, Miyamichi K, Luo L (2014) Presynaptic partners of dorsal raphe serotonergic and GABAergic neurons. Neuron 83:645-662.

Westlund KN, Coulter JD (1980) Descending projections of the locus coeruleus and subcoeruleus/medial parabrachial nuclei in monkey: axonal transport studies and dopamine-beta-hydroxylase immunocytochemistry. Brain Res 2:235-264.

Wiberg M, Westman J, Blomqvist A (1986) The projection to the mesencephalon from the sensory trigeminal nuclei. An anatomical study in the cat. Brain Res 399:51-68.

Witter L, Canto CB, Hoogland TM, de Gruijl JR, De Zeeuw CI (2013) Strength and timing of motor responses mediated by rebound firing in the cerebellar nuclei after Purkinje cell activation. Frontiers in neural circuits 7:133.

Woolf NJ, Butcher LL (1986) Cholinergic systems in the rat brain: III. Projections from the pontomesencephalic tegmentum to the thalamus, tectum, basal ganglia, and basal forebrain. Brain Res Bull 16:603-637.

Woolf NJ, Butcher LL (1989) Cholinergic systems in the rat brain: IV. Descending projections of the pontomesencephalic tegmentum. Brain Res Bull 23:519-540.

Wu J, Capelli P, Bouvier J, Goulding M, Arber S, Fortin G (2017) A V0 core neuronal circuit for inspiration. Nat Commun 8:544.

Yackle K, Schwarz LA, Kam K, Sorokin JM, Huguenard JR, Feldman JL, Luo L, Krasnow MA (2017) Breathing control center neurons that promote arousal in mice. Science 355:1411-1415.

Yamada H, Ezure K, Manabe M (1988) Efferent projections of inspiratory neurons of the ventral respiratory group. A dual labeling study in the rat. Brain Res 455:283-294.

Yang B, Sanches-Padilla J, Kondapalli J, Morison SL, Delpire E, Awatramani R, Surmeier DJ (2021) Locus coeruleus anchors a trisynaptic circuit controlling fear-induced suppression of feeding. Neuron 109:823-838 e826.

Yang CF, Feldman JL (2018) Efferent projections of excitatory and inhibitory preBötzinger complex neurons. J Comp Neurol 526:1389-1402.

Yang CF, Kim EJ, Callaway EM, Feldman JL (2020) Monosynaptic projections to excitatory and inhibitory preBötzinger complex neurons. Front Neuroanat 14:58.

Yates BJ, Grélot L, Kerman IA, Balaban CD, Jakus J, Miller AD (1994) Organization of vestibular inputs to nucleus tractus solitarius and adjacent structures in cat brain stem. Am J Physiol 267:R974-983.

Yatim N, Billig I, Compoint C, Buisseret P, Buisseret-Delmas C (1996) Trigeminocerebellar and trigemino-olivary projections in rats. Neurosci Res 25:267-283.

Yokota S, Tsumori T, Ono K, Yasui Y (2004) Glutamatergic pathways from the Kölliker-Fuse nucleus to the phrenic nucleus in the rat. Brain Res 995:118-130.

Yokota S, Oka T, Asano H, Yasui Y (2016) Orexinergic fibers are in contact with Kölliker-Fuse nucleus neurons projecting to the respiration-related nuclei in the medulla oblongata and spinal cord of the rat. Brain Res 1648:512-523.

Yokota S, Oka T, Tsumori T, Nakamura S, Yasui Y (2007) Glutamatergic neurons in the Kölliker-Fuse nucleus project to the rostral ventral respiratory group and phrenic nucleus: a combined retrograde tracing and *in situ* hybridization study in the rat. Neurosci Res 59:341-346.

Yokota S, Kaur S, VanderHorst VG, Saper CB, Chamberlin NL (2015) Respiratory-related outputs of glutamatergic, hypercapnia-responsive parabrachial neurons in mice. J Comp Neurol 523:907-920.

Yoshida K, McCormack S, España RA, Crocker A, Scammell TE (2006) Afferents to the orexin neurons of the rat brain. J Comp Neurol 494:845-861.

Young JK, Wu M, Manaye KF, Kc P, Allard JS, Mack SO, Haxhiu MA (2005) Orexin stimulates breathing via medullary and spinal pathways. J Appl Physiol (1985) 98:1387-1395.

Yu H, Shi L, Chen J, Jun S, Hao Y, Wang S, Fu C, Zhang X, Lu H, Wang S, Yuan F (2021) A neural circuit mechanism controlling breathing by leptin in the nucleus tractus solitarii. Neurosci Bull.

Yuengert R, Hori K, Kibodeaux EE, McClellan JX, Morales JE, Huang TP, Neul JL, Lai HC (2015) Origin of a non-Clarke's column division of the dorsal spinocerebellar tract and the role of caudal Proprioceptive neurons in motor function. Cell Rep 13:1258-1271.

Zhang CK, Li ZH, Qiao Y, Zhang T, Lu YC, Chen T, Dong YL, Li YQ, Li JL (2018) VGLUT1 or VGLUT2 mRNA-positive neurons in spinal trigeminal nucleus provide collateral projections to both the thalamus and the parabrachial nucleus in rats. Mol Brain 11:22.

Zheng JQ, Seki M, Hayakawa T, Ito H, Zyo K (1995) Descending projections from the paraventricular hypothalamic nucleus to the spinal cord: anterograde tracing study in the rat. Okajimas Folia Anat Jpn 72:119-135.

Zheng Y, Riche D, Rekling JC, Foutz AS, Denavit-Saubie M (1998) Brainstem neurons projecting to the rostral ventral respiratory group (VRG) in the medulla oblongata of the rat revealed by co-application of NMDA and biocytin. Brain Res 782:113-125.

Zoccal DB, Silva JN, Barnett WH, Lemes EV, Falquetto B, Colombari E, Molkov YI, Moreira TS, Takakura AC (2018) Interaction between the retrotrapezoid nucleus and the parafacial respiratory group to regulate active expiration and sympathetic activity in rats. Am J Physiol Lung Cell Mol Physiol 315:L891-L909.
